# Proteomimetic surface fragments distinguish targets by function†

**DOI:** 10.1039/d0sc03525d

**Published:** 2020-09-10

**Authors:** Attila Tököli, Beáta Mag, Éva Bartus, Edit Wéber, Gerda Szakonyi, Márton A. Simon, Ágnes Czibula, Éva Monostori, László Nyitray, Tamás A. Martinek

**Affiliations:** Department of Medical Chemistry, University of Szeged Dóm tér 8 H6720 Szeged Hungary martinek.tamas@med.u-szeged.hu; MTA-SZTE Biomimetic Systems Research Group, University of Szeged Dóm tér 8 H6720 Szeged Hungary; Institute of Pharmaceutical Analysis, University of Szeged Somogyi u. 4. H6720 Szeged Hungary; Department of Biochemistry, Eötvös Loránd University Pázmány Péter sétány 1/C H1077 Budapest Hungary nyitray@elte.hu; Lymphocyte Signal Transduction Laboratory, Institute of Genetics, Biological Research Centre Temesvári krt. 62 H6726 Szeged Hungary

## Abstract

The fragment-centric design promises a means to develop complex xenobiotic protein surface mimetics, but it is challenging to find locally biomimetic structures. To address this issue, foldameric local surface mimetic (LSM) libraries were constructed. Protein affinity patterns, ligand promiscuity and protein druggability were evaluated using pull-down data for targets with various interaction tendencies and levels of homology. LSM probes based on H14 helices exhibited sufficient binding affinities for the detection of both orthosteric and non-orthosteric spots, and overall binding tendencies correlated with the magnitude of the target interactome. Binding was driven by two proteinogenic side chains and LSM probes could distinguish structurally similar proteins with different functions, indicating limited promiscuity. Binding patterns displayed similar side chain enrichment values to those for native protein–protein interfaces implying locally biomimetic behavior. These analyses suggest that in a fragment-centric approach foldameric LSMs can serve as useful probes and building blocks for undruggable protein interfaces.

## Abstract 2

Foldameric local surface mimetics (LSMs) detect spots at protein surfaces and are promising building blocks in a fragment-centric design of xenobiotic structures and protein–protein interaction inhibitors.

## Graphical abstract

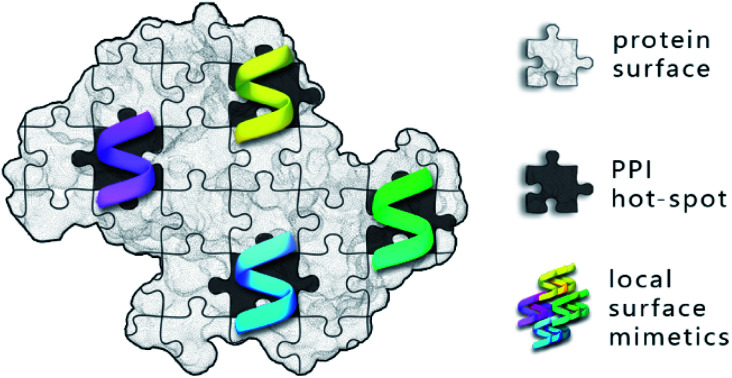

## Introduction

Protein surface mimetics are chemical tools that target protein–protein interactions (PPIs) that are undruggable with small molecules.^1–3^ Although pioneering studies demonstrated the possibility of ribosome-assisted coupling of amino acids with non-natural backbones,^4–7^*in vitro* directed evolutionary approaches^8^ are not routinely available for searching and optimization of fundamentally xenobiotic surface mimetic structures.^9–11^ Four major experimental chemical approaches and their combinations have been applied to address this problem: (i) screening of large surface mimetic libraries,^10,12,13^ (ii) top-down mutational design based on known ligands,^14–21^ (iii) bottom-up design and assembly starting from structural hypotheses,^3,22–31^ and (iv) the fragment-centric system chemistry approach leading to self-assembling ligands.^32^ Recent theoretical and experimental results strongly support that fragment-centric design built on recognition elements of reduced structural complexity is highly promising for the construction of surface mimetic ligands.^33–35^ It is also in accord with the fragment-centric approach that protein interfaces are highly degenerate and strongly interconnected, and interface geometries are not dominated locally by specific secondary structure types.^36,37^ To develop the fragment-centric concept further, the present study aimed to find minimal motifs that can serve as local surface mimetics (LSM) with sufficient affinity to probe otherwise undruggable protein surfaces. Dissociation constants below 100 μM, the fragment-based drug design limit,^38^ would facilitate observation of protein-specific binding patterns and their potential application as surface mimetic building blocks.

**Fig. 1:**
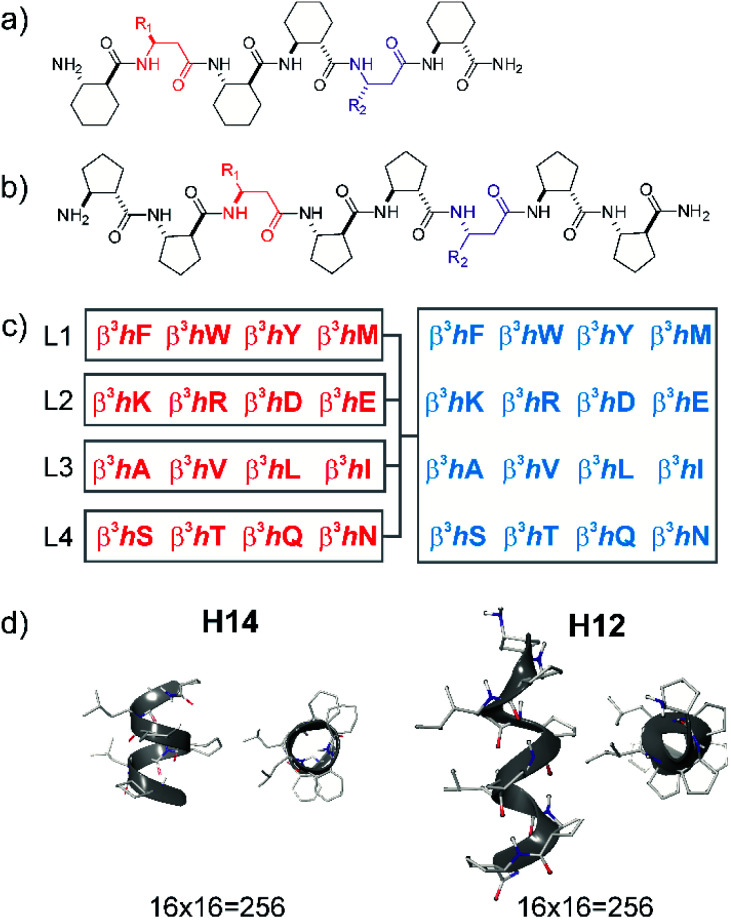
Design of local surface mimetic probe libraries using short foldamers. H14 scaffold (a), H12 scaffold (b), library composition showing sublibrary grouping (c), and structures of H14 and H12 helices (d).

Our hypothesis was that the shortest foldamer sequences with folding tendency in water can be used as LSM probes (Fig. 1). The shortest water-stable foldameric structures are β-peptidic hexamer helices stabilized by a number of cyclic side chains.^39–41^ These sequences display only two proteinogenic side chains on the same side that can be strongly shielded from the solvent by the bulky scaffold and the cyclic side chains. While longer foldameric sequences have been experimentally shown to recognize protein surfaces and inhibit protein–protein interactions (PPIs) for targets such as hDM2,^42,43^ the Bcl-xL family,^44^ gp-41,^45,46^ the VEGF-receptor,^47^ and β-amyloid oligomers,^22^ the general druggability of protein interfaces with foldameric secondary structures has not been established. This might cast doubt on the general compatibility with protein surfaces that unnatural backbone segments are often not tolerated at protein–foldamer interfaces for ligands derived from natural templates in a top-down design scenario.^48,49^ In contrast, compatibility was displayed toward some bottom-up designed foldameric sequences,^50^ which led to high-affinity ligands.

Besides sufficient affinity (druggability), ligand promiscuity is an equally important problem for the design of specific surface mimetic ligands.^51^ It is especially challenging to handle protein interface similarity and ligand promiscuity when exposed protein surfaces are targeted. The relevance of this point for foldamers is indicated by the fact that the chemical space of non-natural residues in bioactive sequences is focused on aliphatic and aromatic hydrophobic side chains that are primarily responsible for ligand promiscuity and non-specific binding.^52,53^ Literature examples exemplify the existence of this phenomenon for peptidic^54^ and aromatic oligoamide foldamers.^25,55^ For acceptable LSM probes, druggability and promiscuity properties should be in balance, mimicking the behaviour of natural proteins. The number of known interactions, and therefore, current knowledge, is limited for druggability–promiscuity properties of foldamers. This situation calls for a systematic approach that generates data on protein–foldamer interactions and facilitates an understanding of molecular recognition patterns depending on the side chain composition, secondary structures of foldamer segments, and target protein surfaces.

In this work, the druggability–promiscuity problem for foldameric LSMs is addressed with a bottom-up fragment-centric pull-down methodology. To test these parameters, target proteins that display different levels of interface similarity and promiscuity in their own PPI network were selected. After mapping LSM binding fingerprints, we found that proposed probes displayed biomimetic features on the protein test set, suggesting the suitability of short foldameric sequences to probe protein surfaces and serve as surface mimetic building blocks.

## Results

### Foldameric LSM probe libraries

Peptide sequences constructed from β-amino acid residues exhibit a strong tendency to fold into secondary structures at short chain lengths, and the specific geometry is controlled by the backbone stereochemistry pattern and topology of structure-inducing side chains.^41,56^ Here, helices H14 and H12 were utilized. The closely packed side chain distances in α-helices (C^α^_*i*_–C^α^_*i*+3_ ≈ 5 Å and C^α^_*i*_–C^α^_*i*+4_ ≈ 6 Å) and in β-sheets (C^α^_*i*_–C^α^_*i*+2_ ≈ 6 Å and “sideways” C^α^_*i*_–C^α^_*i*+*n*_≈ 5 Å) are comparable with the closest side chain distances in foldameric helices H14 (C^β^_*i*_–C^β^_*i*+3_ ≈ 5 Å) and H12 (C^β^_*i*_–C^β^_*i*+2_ ≈ 6 Å). Helix H14 exhibits a strong tendency to fold and its side chains are oriented in parallel. Helix H12 is conformationally less stable in aqueous medium, demonstrating an elongated geometry, and side chains are juxtaposed with a small tilt angle.^41^

These secondary structures can be stabilized by cyclic side chain residues so that they display folding at lengths of hexamers and octamers for H14 ((1*S*,2*S*)-2-aminocyclohexanecarboxylic acid, ACHC) and H12 ((1*S*,2*S*)-2-aminocyclopentanecarboxylic acid, ACPC), respectively. Such oligomers allow two proteinogenic side chains that point to the identical face of the helix to be incorporated into the sequence (Fig. 1). Sixteen different β^3^-amino acids were substituted at positions *R*^1^ and *R*^2^ generating altogether 512 local surface mimetics. LSM probes were synthesized and screened as sublibraries (L1–L4) (Fig. 1c).

This library includes a considerable fraction of peptide foldamers that fold at such a short chain length. These peptides can be considered as minimally folded building blocks in the peptide foldamer space.^32^ These structural units can display a surface area of *ca.* 500 Å^2^ toward a protein surface, which can provide the binding free energy for affinities in the range of 1–500 μM. Such affinity is sufficient for probing the surface and is useful for subsequent fragment-based design.

### Test protein set

**Fig. 2:**
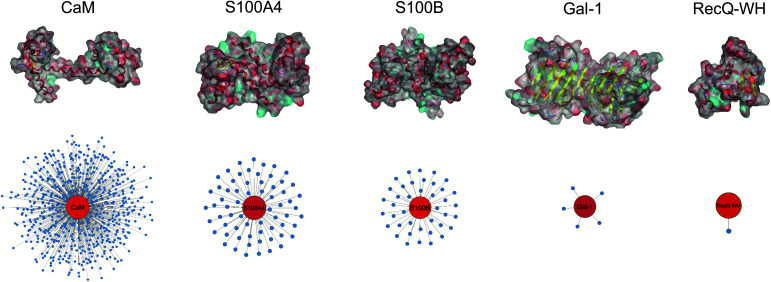
Surface representation and PPI network of selected proteins. PPI data were obtained from BioGRID,^61^ Wiki-Pi,^62^ GPS-Prot,^63^ IntAct^64^ and APID.^65^ For the lectin Gal-1, *N*_PPI_ is based on ref. 66.

Five proteins (Table 1) were selected based on numbers of their interacting protein partners (Fig. 2), levels of interprotein structural homology, and types of interactions (protein–protein, protein–carbohydrate). The reference protein in this set was CaM,^57^ a highly pleiotropic molecule that plays a vital role in every eukaryotic cell. It displays EF-hand motifs and mediates an extremely large number of signal pathways *via* control of enzyme and ion channel activities in a Ca^2+^-dependent manner. Two additional EF-hand proteins with high structural homology were selected from the S100 family: S100A4 and S100B,^58^ both important pharmacological targets for cancer treatment.^59^ S100A4 is involved in tumour progression and metastasis and has many interacting partners. S100B is expressed in melanocyte-derived tumours and mature astrocytes,^60^ where it plays an important role in neurite extension. Numbers of its known protein partners are significantly less than that for S100A4. Galectin-1 (Gal-1) and the winged helix domain of RecQ helicase (RecQ-WH) were used as target models with low tendency to form PPIs. Gal-1 has an immunosuppressive effect, promoting cancer progression and metastasis through recognition of β-galactoside motifs on cell surface glycoproteins.^66^ Gal-1 has a β-sandwich structure with a jelly roll topology,^67–69^ that may interact with proteins in a carbohydrate independent manner.^66,70–72^ RecQ is a prokaryotic intracellular ATP-dependent DNA helicase, which plays important roles in DNA damage response, DNA recombination, replication, and repair.^73,74^ RecQ interacts with the disordered C-terminal of the single-stranded DNA-binding protein (SSB) through its WH domain, which is the single PPI reported for RecQ-WH.^75^ All selected targets exhibit solvent exposed shallow binding sites; however, CaM is able to wrap around α-helices with special side chain motifs, thereby forming a hydrophobic pocket.^76,77^ For competitive binding studies, peptide motifs of PPI partners were selected from the following proteins: transient receptor potential cation channel subfamily V member 1 (TRPV1), non-muscle myosin IIA (NMIIA), ribosomal S6 kinase 1 (RSK1), and SSB, for CaM, S100A4, S100B, and RecQ-WH, respectively.

**Table 1:** Characteristics of applied protein probes

	CaM	S100A4	S100B	Gal-1	RecQ-WH
Helix content (%)	62	55	58	0	50
β-sheet content (%)	4	3	2	54	13
Number of PPIs^a^	645	67	41	5	1
Molecular weight (kDa)	16.8	11.7 × 2^b^	10.7 × 2^b^	14.6 × 2^b^	12.9
Isoelectric point^c^	4.09	5.85	4.52	5.30	10.18

a Average values from databases, BioGRID, Wiki-Pi, GPS-Prot, IntAct, and APID. For Gal-1, *N*_PPI_ is given based on the review of Camby *et al.*^66^

b Homodimers.

c Calculated values based on amino acid composition.

### LSM affinity patterns indicate secondary structure dependent structural compatibility

**Fig. 3:**
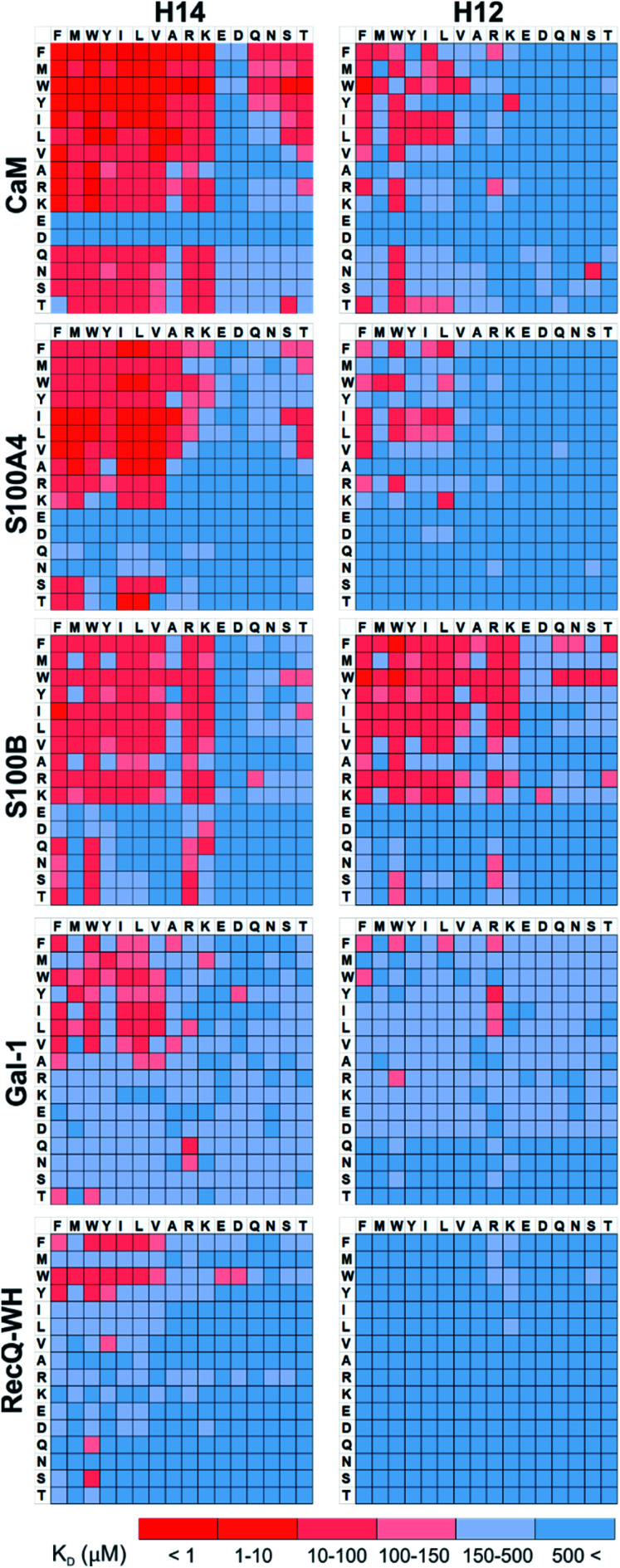
Binding patterns of foldameric LSM probes represented as heat maps in the *K*_D_ dimension (in μM). Apparent dissociation constants are given for H14- and H12-helical LSM libraries. One letter codes displayed at the headings of the rows and columns correspond to the proteinogenic side chains of the β^3^-homo-amino acids used in the *R*^1^ and *R*^2^ positions. (Exact *K*_D_ values are given in the ESI, Tables S1–S5.†)

Pull-down assays with immobilized test proteins and foldameric LSM libraries were carried out. Proteins were equilibrated with LSM probes, and equilibrium bound fractions (*F*_B_) were determined from the unbound fractions measured by HPLC-MS methods. Hits were corrected with background binding of the resin. The protocol allowed all binding events to equilibrate irrespective of affinity, specificity, and binding kinetics. This was essential to judge overall druggability and promiscuity. Importantly, the concentration of protein and the total concentration of foldameric LSMs were equimolar, which minimized competition between the probes. This experimental setup approximated independent binding of individual library members. Results were visualized as heat maps of dissociation constants (Fig. 3). Considering the experimental error of *F*_B_ (see ESI, Fig. S1†), the affinity limit was set at 150 μM to filter successful hits (Fig. 3, indicated in red), which is 50 μM above the 100 μM guideline normally applied in fragment-based drug design.^38^

**Fig. 4:**
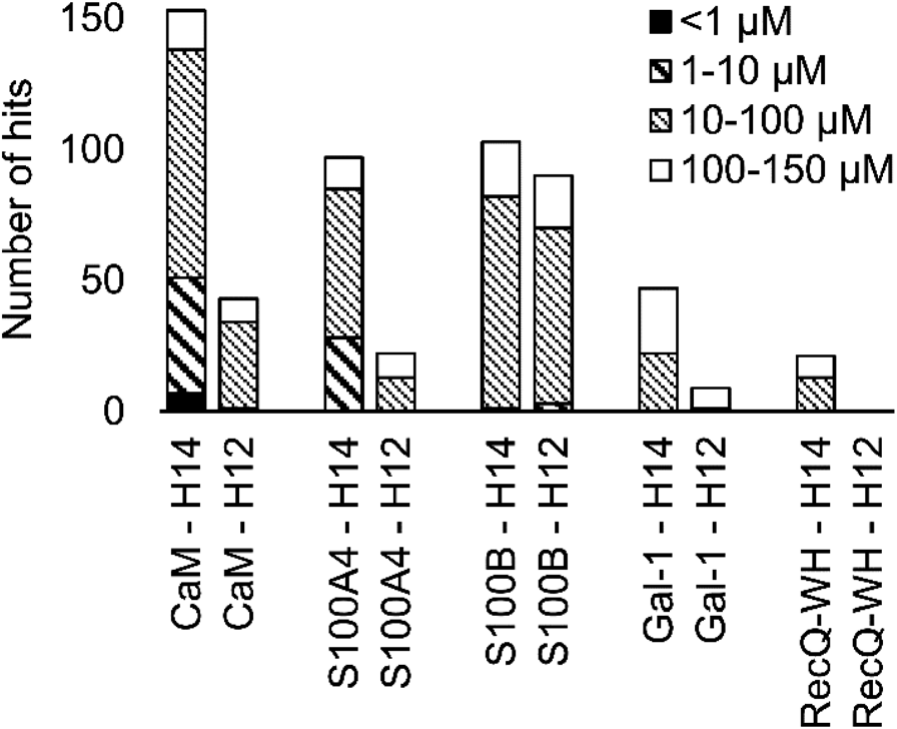
Number of hits obtained in the H14 and H12 LSM pull-down experiments and their affinity distributions.

A number of hits were obtained for all proteins studied, which supports the hypothesis that foldameric LSMs can display sufficient contact area toward target proteins (Fig. 4). Helical geometry had profound effects on binding tendency. Helix H12 yielded fewer hits in general than did H14. Moreover, binders having the H12 skeleton in the affinity range 1–10 μM could only be observed for protein S100B, while H14 displayed more uniform affinity distribution.

Overall, these findings indicated that H14 helical probes had an overall ability to adapt to surface features of the protein set studied. The stable, short, and bulky structure of H14 with its closely packed side chains is free to reorient to attain the optimum affinity as a local probe. Our conclusion was that the H14 library exhibits superior properties for probing protein surfaces. To validate protein binding of this library, selected H14 LSM probes were synthesized separately and tested for binding in the solution phase (see ESI for details, Fig. S2–6†).

### Foldameric LSMs detect orthosteric and non-orthosteric spots

**Fig. 5:**
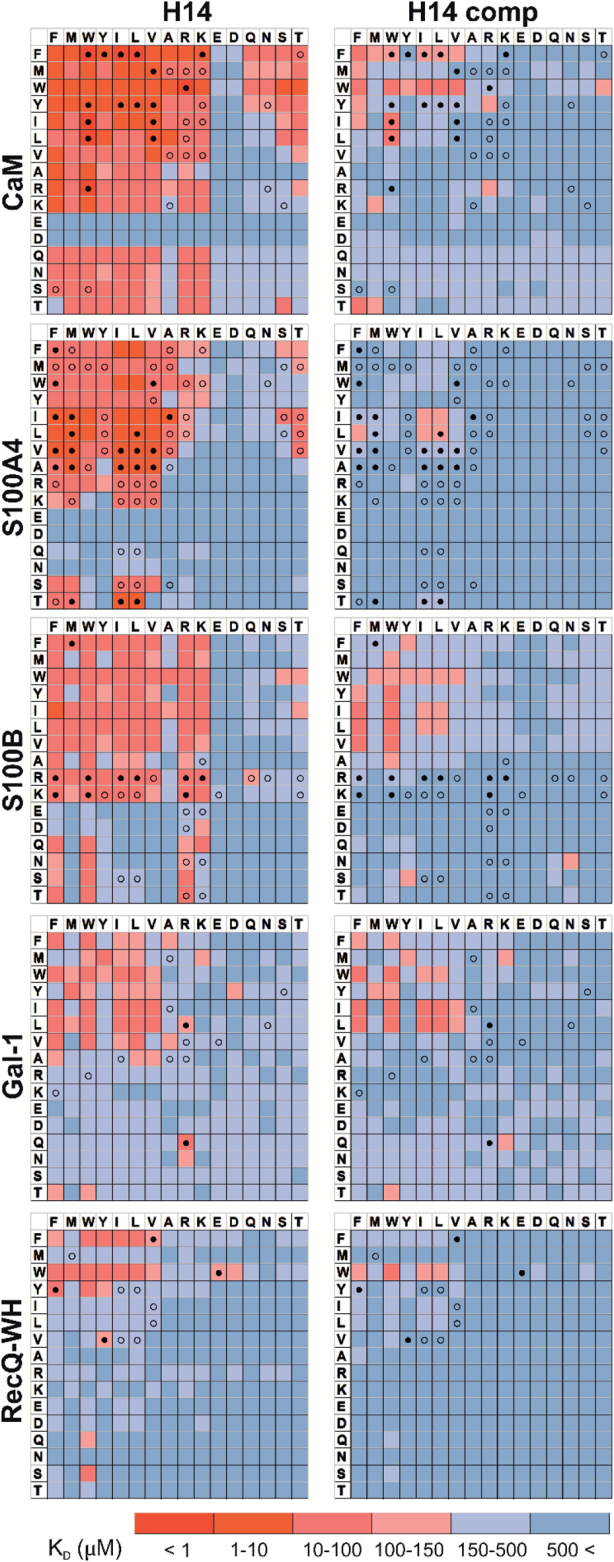
Competition pull-down experiments with the H14 LSM library. *K*_D_ heat maps show side chain preferences using H14 helices alone and in the presence of native ligands as competitors (left and right, respectively). Foldamers with significant replacement percentages (>80% for RecQ-WH, >90% for the other proteins) are marked with an open circle, while foldamer hits with significant *K*_D_ increase are marked with a filled circle. Exact *K*_D_ values are given in Tables S1–S5.†

Interactions between the H14 helical LSMs and the targets were abundant, but safe recognition of the native PPI interfaces on the targets is a criterion of druggability. To gain insight into the ability of the LSMs to detect orthosteric spots, we carried out pull-down assays in the presence of the native ligands (Table 2) of the targets (Fig. 5). For highlighting the effect of the competitor on the binding fingerprints, *K*_D_ ratios and the LSM replacement percentages were calculated and are represented as heat maps (see ESI, Fig. S7†). We found that native ligands radically changed the LSM affinity patterns for targets exhibiting many direct protein–protein contacts (CaM, S100A4, S100B, and RecQ-WH), and many of the LSM probes were displaced. Since these proteins exhibit geometrically a single surface region to form PPI interfaces, residual binders obviously interacted with the non-orthosteric spots. In contrast, only a few LSM probes displayed replacement for Gal-1. This could be explained by the glycan selective native recognition domain of the protein. We note that the replacement pattern for a single probe can, in theory, be complex. While orthosteric weak-binder probes could be completely replaced from interaction sites, high-affinity foldamer hits might bind to targets even in the presence of competitors, although significantly increased apparent *K*_D_ values clearly indicated orthosteric foldamer probes. Moreover, a single probe can bind to both orthosteric and non-orthosteric spots, which limits overall displacement levels. To avoid overlooking interesting orthosteric weak binders, direct replacement levels were assessed (see ESI, Fig. S7†).

**Table 2:** Sequences and dissociation constants of the native ligands applied as competitors

Protein	Competitor	*K*_D_
CaM	TRPV1-Ct_15_	30.9 ± 2.1 nM (ref. 32)
S100A4	NMIIA (1893–1923)	7 ± 1 nM (ref. 83)
S100B	RSK1 (689–735)	1.8 μM (ref. 83)
Gal-1	Lactose	409 μM (ref. 67)
RecQ-WH	SSB-Ct_8_	16.6 ± 0.8 μM (ref. 75)

### Affinity patterns of foldameric LSMs are characteristic of the target proteins

**Fig. 6:**
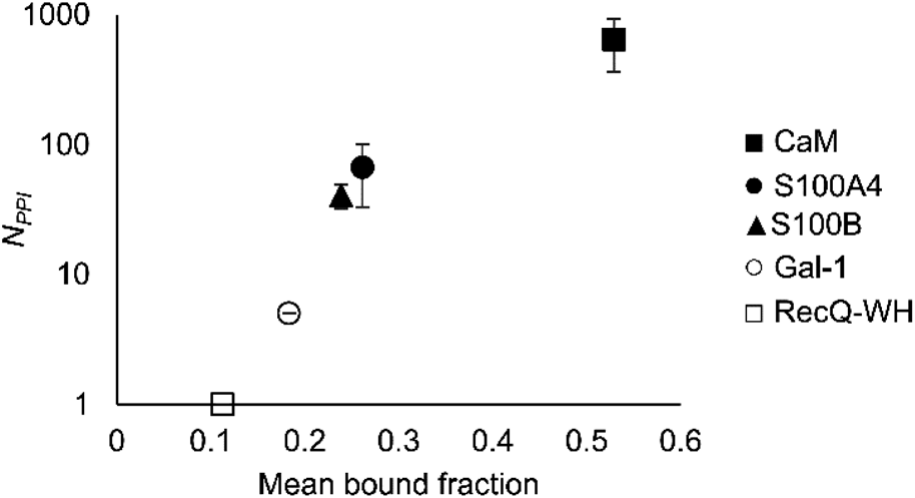
Average bound fractions for the H14 helical LSM library compared with the mean number of PPIs found in databases BioGRID, Wiki-Pi, GPS-Prot, IntAct, and APID. For the lectin, Gal-1, *N*_PPI_ is based on ref. 66.

An important aspect of this study was to test if foldameric LSM probes are able to distinguish proteins in terms of their PPI forming behaviour. First, different levels of PPI promiscuities were compared among test proteins using the overall ability to bind LSM probes. PPI promiscuity was measured with the number of known PPI partners in databases, BioGRID,^61^ Wiki-Pi,^62^ GPS-Prot,^63^ IntAct^64^ and APID^65^ (Fig. 2). False positive rates in proteomics range from 20–34% (ref. 78 and 79) and databases may not provide exact numbers of PPIs.^80,81^ Nevertheless, these data can be used to indicate the magnitude of promiscuity and protein moonlighting.^82^ The binding promiscuity in foldameric LSM space was estimated with the average bound fractions in the H14 library. A connection was found between the number of database PPI partners (*N*_PPI_) and the average bound fraction values obtained for the H14 helical LSM probe library (Fig. 6, and ESI, Fig. S8†). Logarithmic scaling is explained by exponential relationships between the fragment space of LSM probes and the chemical space of full proteins. This finding suggests that the overall affinity of the H14 LSM probes distinguishes among proteins with strongly different levels of promiscuity. With this behaviour, foldameric LSM probes displayed a biomimicking feature, an essential criterion for surface mimetic drug design.

The ability to distinguish between proteins can also be measured by similarity between the affinity patterns. Such similarity was calculated using pairwise covariance of *F*_B_ values scaled to the theoretical maximum value for the 256-membered library. Scaled covariance indicates maximum similarity with a value of 100%, while zero covariance leads to 0%.

**Fig. 7:**
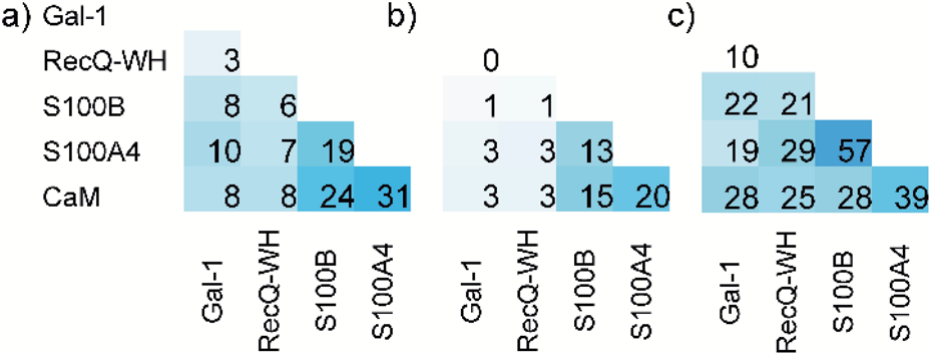
Pairwise scaled covariances (%, maximum similarity: 100%, zero covariance: 0%) of H14 LSM *F*_B_ patterns calculated for total (a) and the orthosteric (b) *F*_B_ values. %. The sequence homologies of proteins are given in panel (c).

Results of the calculations are displayed for total (Fig. 7a) and the orthosteric (Fig. 7b) binding fractions. Pairwise scaled covariances were uniformly lower for the orthosteric binders, showing that non-orthosteric binding obscured differences between proteins as detected by the affinity pattern of the LSM probes. This finding supports that the non-orthosteric binding for these proteins can be considered non-specific in nature and is a source of promiscuous behaviour for LSM probes. However, non-orthosteric spots can contribute to allosteric sites, which can be detected with the LSM probes. In this study, CaM, S100A4, and S100B were selected as proteins with high levels of structural and functional homology, albeit their specific roles and pathways in signal transduction are different and require different PPI interfaces. Scaled covariances for the orthosteric binders in the range of 13–20% were found among these proteins. This low level of interface similarity detected by LSM affinity patterns supports the conclusion that LSM probes are able to distinguish the proteins with different functions even with high levels of structural similarity (Fig. 7c).

### Side chain binding propensities are biomimetic

Features at the level of the side chains that are responsible for binding of LSM probes were also investigated. The analysis of the affinity patterns revealed that binding affinity did not depend on the dominant presence of highly hydrophobic cyclic β-amino acid residues (ACHC and ACPC). If the sheer hydrophobic surface of these residues drove interaction with the test proteins, higher uniform baseline binding would have been observed. Many sequences displayed no significant binding. Interestingly, certain levels of diagonal symmetry could be observed in the affinity maps (Fig. 3), suggesting that the direction of the order of the *R*^1^ and *R*^2^ proteinogenic side chains exhibits a limited effect on binding. These findings indicate that protein–LSM interactions are driven by proteinogenic side chains present on the compact scaffold.

Focusing on the native-like anchor points, normalized frequencies (*w*_j_) of the residues at protein-LSM contacts were analysed. The literature definition of *w*_j_^37^ is given in eqn (1):1
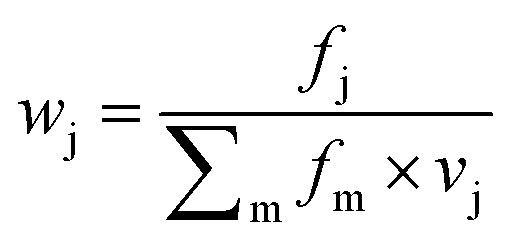
where *f*_j_ is the number of interface residues of type j, and normalization factor *v*_j_ is the overall prevalence of the amino acid of type j. Index m denotes the residue type. To approximate residue frequencies based on bound fractions for the H14 LSM library, the following formula was applied:2
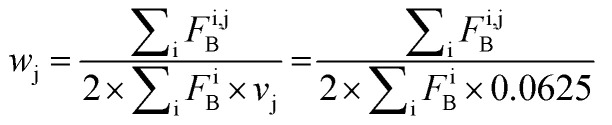


**Fig. 8:**
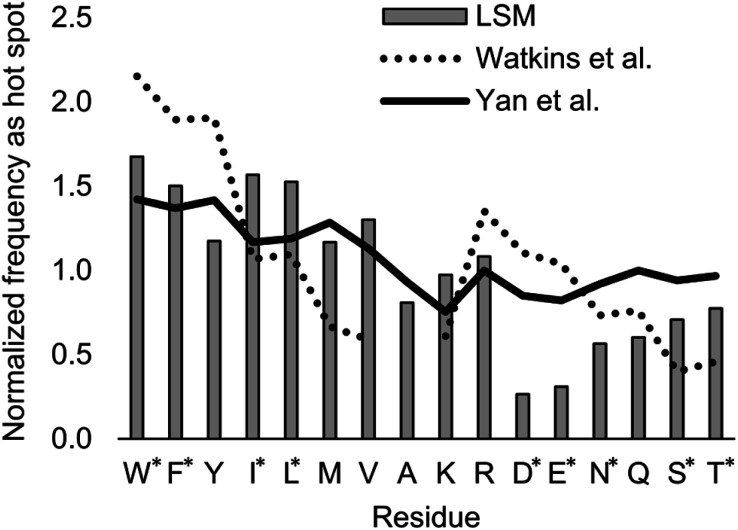
Normalized frequencies obtained from literature data and calculated for the H14 LSM library. Values from Watkins *et al.*^37^ and Yan *et al.*^84^ are based on computational Ala scanning and on PPI interface analysis, respectively. LSM data were calculated from experimentally determined *F*_B_ data using eqn (2). The enrichment and depletion of residues marked with asterisks (*) were found to be statistically significant (Z-score, *p* < 0.05).

Index i denotes the LSM sequence, and index j is the residue type. *F*_B_^i,j^ is the bound fraction of the LSM probe i, if it contains residue type j. *F*_B_^i,j^ is zero, if sequence i does not contain residue type j. This formula assumes that a binder LSM probe has both proteinogenic side chains in contact with the protein interface. The LSM library was uniform with respect to the 16 side chains, and therefore *v*_j_ = 1/16 = 0.0625 was applied as a uniform normalization factor. Normalized frequencies were compared with literature data dissecting large datasets from the Protein Data Bank: (i) computational Ala scanning categorized by secondary structure types^37^ and (ii) protein–protein interface analysis, defining interface residues with a maximum distance of 6 Å for the contacting residues^84^ (Fig. 8, calculation details in the ESI†).

The LSM library displayed enrichment for residues W, F, Y, I, L, M, V, and R and overall depletion for residues A, D, E, N, Q, S, and T was observed. Statistically significant (Z-score, *p* < 0.05) changes were observed for W, F, I, L, D, E, N, S and T (Table S6†). These results are qualitatively consistent with analyses of the PPI interface databases that demonstrate enrichment of aromatic^37,84^ and aliphatic side chains at PPI interfaces,^85^ along with the importance of ion pair interactions.^84,86^ Interestingly, engineered proteins exhibit a more dominant enrichment in aromatic and ionic residues.^87^ However, variations of the side chain frequencies have been observed across different structural databases.^37,84,85,88,89^

For the test protein set, side chain frequency levels were dependent on specific proteins (see ESI, Fig. S9†), and enrichment/depletion values can be associated with the selectivity of PPI interactions. Initially, foldameric LSM probes were hypothesized to respond to key side chain requirements of binding at orthosteric sites, especially sites involving electrostatic and H-bonding interactions.

For CaM, a high-affinity interaction is possible only if cationic residues are present in specific patterns on the mostly hydrophobic helical interface.^90^ Inspection of binding data (Fig. 3) clearly revealed that residues Lys and Arg were enriched in the binder LSM probes. S100A4 interacts with sequences containing Ser, Thr, and Met as key residues.^91^ The selection toward these side chains is evident in binding fingerprints of S100A4. Protein S100B displays different binding preferences compared with S100A4.^92^ In protein complexes of S100B, besides Leu and Ile, cationic side chains are also prevalent as anchoring points of interacting partners.^83,93–95^ NMR experiments reported that the binding surface of the S100B-p53 peptide complex is defined by Arg, Lys, Leu, and Ile residues of p53, forming salt bridges with Glu side chains and participating in hydrophobic contacts with a hydrophobic patch on S100B.^83,93–95^ As opposed to S100A4, the specific Arg–Lys block was observed in LSM probe binding fingerprints of S100B.

Gal-1 and the RecQ-WH domain were models for proteins with a low tendency to form direct protein–protein complexes. Gal-1 does not exhibit preference for specific hydrophilic side chains, and accordingly, its LSM affinity pattern did not show a tendency to bind other than hydrophobic side chains. The binding cleft of RecQ-WH includes a hydrophobic graft flanked by polar and positively charged residues^96^ that recognizes the intrinsically disordered and highly anionic C-terminal of SSB^97^ (DFDDDIPF). This segment fits into a narrow and slightly bent site on RecQ-WH. As expected, surface mapping of RecQ-WH with bulky LSM probes led to a low number of hits. Even the propensity of the protein to bind anionic sequences did not drive anionic LSM probes to the binding site, possibly because of steric incompatibility.

These findings suggest that the protein interfaces studied select preferred side chain chemistries from the LSM probe library in a manner similar to selection of natural partners. On this ground, we concluded that LSM probes exhibit biomimetic features in presenting proteinogenic side chains for their protein partners.

## Conclusions

Foldameric LSM probe libraries were constructed with H14 and H12 scaffolds and were tested with five proteins. Affinity patterns of 512 LSM probes were recorded, and the resulting 2560 protein–foldamer interactions were analysed using a fragment-centric approach. LSM affinities were secondary structure dependent, and a sufficient number of hits in the affinity range of 1–150 μM was obtained for the hexameric H14 library. Binding was driven by two proteinogenic side chains displayed. The hydrophobic foldamer scaffold served as a template and solvent shield. The better performance of H14 LSM probes may be explained by their ability to geometrically adapt their stable and bulky skeleton to the local environment at protein hot spots. Foldameric LSMs sensitively probed protein surfaces, and thus, detected both orthosteric and non-orthosteric hot spots, based on competition experiments. Orthosteric hits identified in this way along with their geometrically confined binding to the PPI interface offer the possibility of linking them to a high-affinity ligand. This concept was demonstrated earlier in a dynamic covalent system with CaM.^32^ Despite the limited structural complexity of LSM probes, affinity fingerprints were characteristic of target proteins. The overall tendency to bind LSM probes correlates with the number of PPI partners identified for the protein. Pairwise correlations between orthosteric binding patterns remained low even for proteins with high structural homology. LSM probes are thus sensitive to the function encoded by PPI interfaces. LSM probes achieve such biomimetic behaviour by presenting proteinogenic side chains in a manner similar to natural PPIs. This behaviour is reflected in protein-like enrichment and depletion values obtained for the side chain chemistries. Moreover, test proteins were able to select key residue types needed for specific PPI recognition patterns from the libraries.

In the early days of chemical biology, Linus Pauling proposed target-induced self-organization of biopolymers as a possible mechanism to generate high-affinity ligands against proteins.^98^ His chemistry-inspired concept did not explain the operation of the adaptive immune system, but Pauling's ingenious idea is still there mostly untapped to offer a route to synthetic biopolymers that mimic molecular recognition properties of natural antibodies. This work takes a step toward Pauling's concept, using self-organizing protein mimetic LSM probes as promising candidates for recognition segments. An additional understanding of dynamic covalent chemistry may eventually solve the problem of *in situ* coupling.

## Conflicts of interest

There are no conflicts to declare.

## Supplementary Material

SC-011-D0SC03525D-s001
