# Supplementary material for: Proteomimetic surface fragments distinguish targets by function
Source: Chem Sci. 2020 Sep 10;11(38):10390–8. doi: 10.1039/d0sc03525d (PMC8162404; doi:10.1039/d0sc03525d)
Supplement: SC-011-D0SC03525D-s001 [file SC-011-D0SC03525D-s001.pdf]

Electronic Supplementary Material (ESI) for Chemical Science.
This journal is © The Royal Society of Chemistry 2020

Electronic supplementary information

for

Proteomimetic surface fragments distinguish targets by function

Attila Tököli[a], Beáta Mag[a], Éva Bartus[a,b], Edit Wéber[a], Gerda Szakonyi[c], Márton A. Simon[d], Ágnes Czibula[e], Éva Monostori[e], László Nyitray[d,*], Tamás A. Martinek[a,b,*]

[a]Department of Medical Chemistry, University of Szeged, Dóm tér 8. H6720, Szeged, Hungary; [b]MTA-SZTE Biomimetic Systems Research Group, University of Szeged, Dóm tér 8. H6720, Szeged, Hungary; [c]Institute of Pharmaceutical Analysis, University of Szeged, Somogyi u. 4., H6720, Szeged, Hungary; [d]Department of Biochemistry, Eötvös Loránd University, Pázmány Péter sétány 1/C, H1077, Budapest, Hungary; [e]Lymphocyte Signal Transduction Laboratory, Institute of Genetics, Biological Research Center, Temesvári krt. 62., H6726 Szeged, Hungary

László Nyitray[d.*], Tamás A. Martinek[a,b,*]

Table of contents

Experimental Procedures 3
Peptide synthesis 3
Expression and purification of proteins 4
Pull-down assay and data analysis 5
Calculation of the discussed parameters 6
Binding tests with selected LSM probes..8
Results and Discussion..9
Validation of selected foldamer-protein interactions in solution phase. 9
Supplementary Figures and Tables 10
....10
Fig. S1. Useful affinity window of pull-down measurements considering experimental error. 10
Fig. S2. Structure of the selected H14 LSM probes tested with FP and NMR measurements. 11
Fig. S3. Independent binding tests for selected foldamer probes and S100A4 protein. ...12
Fig.S4. Independent binding tests for selected foldamer probes and S100AB protein. 13
Fig.S5. Independent binding test for compound **1a** and Gal-1 protein............................14
Fig.S6. ^{1}H NMR signal attenuation of **1b** and **12b** compounds in the presence of Gal-1 indicates binding of the foldamers to Gal-1..15
Fig.S7. Competition maps of the H14 libraries based on two different calculations.........16
Fig.S8. Average bound fractions for H14 helical LSM library compared with the number of the PPIs found in data-bases BioGRID, Wiki-Pi, GPS-Prot, IntAct and APID. 17
Fig.S9. Normalized residue frequencies as hot spots for different proteins obtained from experimental FB values for H14 LSM libraries. 18
Fig.S10. Estimation of equimolarity and purity of the 64-membered H12 sublibraries. ...19
Table S1. Surface mapping K_D results obtained for CaM. 20
Table S2. Surface mapping K_D results obtained for S100A4. 21
Table S3. Surface mapping K_D results obtained for S100B. 22
Table S4. Surface mapping K_D results obtained for RecQ-WH. 23
Table S5. Surface mapping K_D results obtained for Gal-1...24
Table S6. One-sample one-tailed Z-scores for the "normalized frequency as hot spot" values of the H14 LSM library side-chains. 25
Table S7. Characterization data of H12 aromatic sublibrary. 26
Table S8. Characterization data of H12 apolar sublibrary. 27
Table S9. Characterization data of H12 charged sublibrary. 28
Table S10. Characterization data of H12 polar sublibrary. 29
Peptide characterisation data 30
Table S11. Molecular mass and m/z data of separately synthetized foldamers...............30
Dataset S1. HPLC-MS data 31

Experimental Procedures

Peptide synthesis

Synthesis and purification of folded fragment libraries. Technically, each library was divided into four 64-membered sublibraries, which were synthetized, analyzed, and screened separately as previously described.[1] Foldamer libraries were synthesized using a CEM Liberty 1 microwave peptide synthesizer with a manual addition of amino acids. The four sublibraries consisted of aromatic and β^3-hMet (L1); charged (L2); non-polar (L3), and non-charged, polar (L4) amino acid sidechains. Rink Amide PS resin was used for solid support, and HATU (1-[bis(dimethylamino)methylene]-1*H*-1,2,3-triazolo[4,5-b]pyridinium 3-oxid hexafluorophosphate) was used as a coupling reagent. Fmoc-(1S,2S)-ACHC ((1S,2S)-Fmoc-2-aminocyclohexane carboxylic acid) or Fmoc-(1S,2S)-ACPC ((1S,2S)-Fmoc-2-aminocyclopentane carboxylic acid) was added in excess of three equivalents at 75°C for 30 min. β^3-amino acid mixtures were double coupled using 0.8 equivalents at 75°C for 45 min. Sixteen different β^3-amino acids were coupled in positions R^1 and R^2, yielding 64 different components in each sublibrary. The deprotection solution was 2% piperidine and 2% DBU (1,8- diazabicycloundec-7-ene) in DMF (*N, N*-dimethylformamide), and deprotection was carried out for 10 min at 75°C. The foldamer mixture was cleaved by 90% TFA (trifluoroacetic acid), 5% DTT (1,4-dithiothreitol), and 5% water. TFA was evaporated, and the resin was washed with acetic acid and water. The mixture was lyophilized. The library was purified by using RP-HPLC (Phenomenex Luna C18, 250x10 mm column). Fractions were analyzed by MS, and fractions containing library members were pooled. Library components were identified by HPLC-MS based on molecular weight and retention time estimated by hydrophobic properties. Purity analysis was based on quantification of total library members, and impurities were assessed by integration of the HPLC-MS chromatograms. Library members have free N- and amidated C-termini.

Synthesis of competitor peptides. Peptides NMIIA (1893 - 1923) and RSK1 (689-735) were produced recombinantly in Escherichia coli BL21 (DE3) cells (Novagen) with TEV-cleavable N-terminal GST-tag, and purified by GST affinity chromatography. The tag was cleaved by TEV protease. After cleavage, TEV protease and GST tag were eliminated by heat denaturation and centrifugation. The supernatant was purified by RP-HPLC using a Jupiter 300 Å C4 column (Phenomenex, Torrance, CA, USA) and the relevant fractions were lyophilized. Concentrations of recombinant peptides were measured by spectrophotometry using absorbance of the Tyr residue. Quality of expressed peptides was checked by mass spectrometry (Bruker Daltonics, Billerica MA, USA). α-peptides for competitive pulldown assay with CaM and RecQ were synthetized using standard Fmoc-based solid phase peptide synthesis methods. The following sequences were prepared: TRPV1-Ct15: GRHWKNFALVPLLRE-NH_2, non-muscle myosin IIA (1893-1923): YRKLQRELEDATETADAMNREVSSLKNKLRR-NH_2, RSK1 (689-735): QDLQLVKGAMAATYSALNSSKPTPQLKPIESSILAQRRVRKLPSTTL-NH_2 and SSB-Ct8: WMDFDDDIPF.

Synthesis and purification of pure foldamer sequences. Foldameric sequences were synthesized manually using standard solid-phase peptide synthesis with Fmoc chemistry. Tentagel R RAM resin was used as solid support and HATU as coupling reagent. Amino acids and coupling reagents were used in excess of 3 equivalents with shaking at room temperature for 3 h. Deprotection was carried out using DMF solution containing 2 % DBU and 2 % piperidine. Cleavage was performed with TFA/H_2O/DTT/TIS (triisopropylsilane) (90:5:2.5:2.5), which was followed by precipitation in ice-cold diethyl ether. Resin was washed with acetic acid and water, filtered, then lyophilized. Peptides were purified by RP-HPLC on a C18 column (Phenomenex Jupiter, 10x250 mm). HPLC eluents were 0.1% TFA in water and 0.1% TFA, 80% ACN in water. Purity was confirmed by analytical RP-HPLC and ESI MS measurements

Synthesis of 5(6)-Carboxyfluorescein (CFU) labeled peptides for fluorescence polarization (FP) experiments. CFU-labeled peptides were synthesized on a solid support with C-terminal 4-methyltrityl (Mtt)- protected lysine. Mtt protecting group was eliminated by washing the resin with TFA/TIS/DCM (1:1:8) for 15 times and the resin was neutralized with 5% DIEA/DMF for 15 min. Carboxyfluorescein was coupled to the ε-amino group of lysine in excess of 3 equivalents with HATU/DIEA activation. Crude peptides were cleaved from the resin with a mixture of TFA/H2O/DTT/TIS (90:5:2.5:2.5) and followed by precipitation in ice-cold diethyl ether. Resin was washed with ACN and water, then filtered and lyophilized. Crude peptide was purified by RP-HPLC on a C18 column (Phenomenex Luna, 250 x 10.00 mm).

Expression and purification of proteins

CaM. Calmodulin (CaM) (bovine) gene was cloned into pET28a vector. Sequenced plasmid was then transformed to competent E. Coli BL21 (DE3) cells for protein expression. Cells were grown on LB liquid media at 37°C until OD_{600} = 0.5, then expression of CaM was induced by adding 200 µM IPTG and was carried out overnight (~19 hours) at 22°C. After centrifugation, cell pellets were resuspended in Ni-NTA Lysis Buffer (50 mM NaH_2PO_4, 300 mM NaCl, 10 mM imidazole, pH 8.0) and were lysed by sonication with addition of 1 µM Leupeptin, 0.1 µg/ml Pepstatin A and 20 µM PMSF (phenylmethylsulfonyl fluoride). Cleared lysate was first purified using a Ni-NTA filled column according to the manufacturer's protocol. After equilibration of Ni-NTA column, lysate was added for a short incubation on ice (30 min), washed with Ni-NTA Wash Buffer (50 mM NaH2PO4, 300 mM NaCl, 20 mM imidazole, pH 8.0), and finally CaM was eluted with small volumes of Ni-NTA Elution buffer (50 mM NaH_2PO_4, 300 mM NaCl, 250 mM imidazole, pH 8.0). Clear fractions were concentrated using Amicon Ultra Filter Device (10K) and buffer was changed to 20 mM HEPES, pH 7.0, 150 mM NaCl. Purity and folding was assessed by HPLC-MS, native ESI-MS and NMR measurements.

S100A4 and S100B. S100A4 and S100B: S100 proteins were expressed and purified as described previously.(1) Briefly, S100 proteins were cloned into a modified pET15b expression vector and were expressed in Escherichia coli BL21(DE3) cells (Novagen, Kenilworth, NJ, USA) with an N-terminal His6-tag, and purified by Ni^{2+}-affinity chromatography. It was followed by hydrophobic interaction chromatography using phenyl sepharose column and applying standard conditions. Quality of the recombinant proteins was checked by SDS-PAGE analysis. Samples were dialyzed against the buffer containing 20 mM HEPES pH 7.5, 150 mM NaCl, 1 mM $CaCl_2$ and 500 µM TCEP overnight. Concentrations were determined by using the absorbance of Trp and Tyr residues.

RecQ-WH. Gene coding the RecQ-WH (coding for residues 408–523 of E. coli RecQ) was amplified in PCR using DH5 alpha genome as template and was cloned into pET28a expression vector. After transforming pET-WH into BL21(DE3) cells, an overnight starter culture was used to inoculate LB media (1:100 ratio), which was incubated at 37°C with vigorous shaking (200 rpm) until mid-log phase (OD_{600}=0.6). Protein expression was induced with 1 mM IPTG (isopropyl-β-D-thiogalactoside) and incubated for 5 hours in the same environment. Cells were harvested by centrifugation, resuspended in lysis buffer (20 mM TRIS, 300 mM NaCl, 1 mM β-mercaptoethanol (BME), 10% glycerol) and disrupted by sonication using cOmplete™ EDTA-free Protease Inhibitor Cocktail according to the manufacturers protocol. Cell debris were pelleted in centrifuge, supernatant was purified by using IMAC chromatography, and concentrated using Amicon Ultra Filter Device (10K). Purity was determined by HPLC-ESI-MS. For pull-down experiments, buffer was exchanged to 20 mM HEPES, 150 mM NaCl pH = 7.4 buffer.

Gal-1. cDNA of human Gal-1 was cloned into a pETHis vector. The recombinant Gal-1 with a His6-tag and three linker amino acids at its N-terminus (His-Gal-1) was expressed in E. coli BL21(DE3) strain. His-Gal-1 was affinity purified from cleared lysate using [illegible]-Lactose Separopore 6B-CL (Emelca) agarose beads. The elution was performed with lactose (50 mM Lactose, 50 mM Tris, pH 7.5; 4 mM BME) and the protein fractions were dialyzed against 10 mM ammonium-acetate and 4 mM BME. Following sterile filtration, concentration of His-Gal-1 was determined and purity was checked by SDS polyacrylamide gel electrophoresis. The lyophilized His-Gal-1 was stored at -80°C in single-use aliquots.

Pull-down assay and data analysis

Pull-down assay. LSM sublibraries were screened separately as previously described.(2) Experiments were performed in the following buffers: (i) 20 mM pH 7.4 HEPES, 150 mM NaCl, 5 mM $CaCl_2$ for CaM; (ii) 50 mM pH 7.4 HEPES, 150 mM NaCl, 1 mM TCEP, 1 mM $CaCl_2$ for S100 proteins; (iii) 20 mM pH 7.4 HEPES, 150 mM NaCl, 1 mM TCEP for Gal-1 and (iv) 20 mM pH 7.4 HEPES, 150 mM NaCl for RecQ-WH. Assays were performed in paper filter spin cups (Thermo Scientific) with sample volumes of 100 µl. Hexahistidine-tagged proteins were immobilized on previously washed Co-NTA resin (TALON, Takara Bio USA, Inc., Mountain View, CA) in 64 µM final concentration. LSM sublibrary was added, in which each of the 64 library members were used at a concentration of 1 µM. Samples were incubated at room temperature for 30 min with shaking at 100 rpm. Then, samples were centrifuged at 1000 rpm for 2 min and washed with 100 µl pull-down buffer to remove unbound compounds. The control experiments were performed using the same compounds and the resin but without the immobilized protein. In case of competitive assays, competitor peptides were added to LSM sublibrary samples in 200 µM final concentration. In the case of Gal-1, lactose (competitor) was used in a concentration of 10 mM. Experiments were performed at least twice. Mean standard deviation of bound fractions obtained from independent experiments were found to be 0.049.

LC-MS methods and parameters. HPLC/ESI-MS analysis was used to characterize the samples from the pull-down assay. LC-MS analysis was performed with a Thermo Scientific Dionex UltiMate 3000 HPLC system interfaced to an LTQ ion trap mass spectrometer (Thermo Electron Corp., San Jose, CA, USA). Samples were injected onto an Aeris Widepore XB-C18 (250 x 4.6 mm) analytical HPLC column using gradient elution 5-80 % solution B during 25 minutes. For pull-down samples, eluent composition was 0.1% acetic acid in distilled water (Solution A) and 0.1% acetic acid in acetonitrile (solution B). Mass spectra were acquired in full scan mode from 200 to 2000 m/z range. For overlapping peaks, selective reaction monitoring (SRM) was used.

MS data analysis. Thermo Xcalibur 2.2 software was used for peak identification and integration. The majority of the foldameric fragments could be resolved independently via HPLC-MS/MS measurements based on molecular weight, MS fragmentation pattern and retention time. Peaks of some foldamers with β^3-*h*Ile or β^3-*h*Leu in position R^2 could not be resolved, these were integrated and averaged. In the processing method, each sample component was associated to a chromatographic peak based on previously identified mass (m/z) and retention time (ref (2) and Table S7-10). Using ICIS peak detection algorithm, the general detection and integration criteria were: smoothing points: 5, baseline window: 80, area noise factor: 5, peak noise factor: 10. Using these processing setups, all raw data files were reprocessed together and analyzed. Errors in peak identification during the automatic processing were corrected manually.

Calculation of the discussed parameters

1. **Bound fractions (F_B)** were calculated from the HPLC-MS intensity loss of the LSM probes compared to the control where no protein was immobilized to the resin:

$$F_B^i = 1 - \frac{AUC_{protein}^i}{AUC_{control}^i}$$

where $AUC^i_{protein}$ and $AUC^i_{control}$ are obtained for compound i in the LSM library in the experiment with immobilized protein and in the control experiment without protein, respectively.

2. **Dissociation constant for the LSM probes (K_D).** As the stoichiometry was a priori not know, 1 : 1 binding was assumed for S100A4, S100B, Gal-1 and RecQ-WH proteins and apparent K_D values were calculated for each LSM probe using the standard formula:

$$K_D = \frac{c_{free\ ligand}^i \times c_{free\ protein}}{c_{complex}^i} = \frac{(1 - F_B^i) \times (64 - \sum_i^N F_B^i)}{F_B^i} \times 10^{-6}$$

In the case of CaM, ITC experiments revealed that CaM is able to bind 2 hexameric H14 foldamers with approximately identical K_D values. Thus, in the case of CaM, 1 : 2 stoichiometry with equivalent binding sites was supposed:

$$K_D = \frac{c_{free\ ligand}^i \times c_{free\ protein}}{c_{complex}^i} = \frac{(1 - F_B^i) \times (64 \times 2 - \sum_i^N F_B^i)}{F_B^i} \times 10^{-6}$$

3. **Replacement percentages.** Replacement percentages in Fig. S6 were obtained by the following formula:

$$Replacement\ percentage_i = \frac{F_{B\ (no\ comp)}^i - F_{B\ (comp)}^i}{F_{B\ (no\ comp)}^i} \times 100$$

with the following restrictions: (i) K_D > 150 µM compounds were excluded from the calculations and (ii) apparent dissociation constants in competition experiments were maximized in 500 µM.

4. **K_D ratios.** K_D ratios in Fig. S6 were calculated as follows:

$$K_{D\ ratio}^i = \frac{K_{D\ (comp)}^i}{K_{D\ (no\ comp)}^i}$$

K_D values were maximized in 1000 µM.

5. **PPI contact number N_{PPI}:**
PPI database information were calculated from the average number of PPI partners obtained from *BioGRID,(3) Wiki-Pi,(4) GPS-Prot(5) and IntAct.(6)* (Fig. 2. and Table 1.).

A considerable amount of Gal-1 binding proteins are glycoproteins and are known to interact with Gal-1 through their carbohydrate moiety. Thus, in order to demonstrate real PPIs through polypeptide chains, Gal-1 interaction records where partners obviously interact with Gal-1 through carbohydrates were not included in the N_{PPI} of Gal-1.

Graph representations in Fig. 2. were prepared by Cytoscape_3.7.2 (7) using N_{PPI} data.

6. **Average bound fractions:**

$$F_B^{mean} = \frac{\sum_i^N F_B^i}{N}$$

where F_B is the bound fraction, index *i* indicates the specific LSM probe sequence and *N* is the total number of the LSM probes in the library (N=256).

7. **Hot spot frequencies of the residues (w_j):**

Normalized hot spot frequencies (w_j) are defined by the frequencies of the residues regarded as hot spot /interface residues normalized with the amino acid distribution in the dataset (v_j).

$$w_j = \frac{f_j}{\sum_m f_m \times v_j}$$

where f_j is the number of interface residues of type j, and indices j and m denote the residue type. v_j is the overall prevalence of the amino acid of type j in the dataset (8).

Residue frequencies as hot spots in the H14 LSM library were calculated as follows:

$$w_j = \frac{\sum_i F_B^{i,j}}{2 \times \sum_i F_B^i \times v_j} = \frac{\sum_i F_B^{i,j}}{2 \times \sum_i F_B^i \times 0.0625}$$

Index i denotes the LSM sequence, index j stands for the residue type. $F_B^{i,j}$ is the bound fraction of the LSM sequence i, if it contains residue type j. $F_B^{i,j}$ is zero, if sequence i does not contain residue type j. As normalization factor, v_j = 1/16=0.0625 was used, which is the fraction of the individual proteinogenic residues in the LSM library.

8. **One-tailed Z-test of side chain normalized hot-spot frequencies:**

Z-score was calculated using the formula below, where the mean hypothesized value (μ_0) is 1, which corresponds to the "normalised frequency as hot spot" value for a residue which is populated to a portion of 1/16=0.0625 at the protein surface. Mean value ($\bar{x}$) is calculated for each side chain individually using averaged frequencies for all five proteins. σ is standard deviation of the hot spot frequencies calculated for each side chain. *n* is the number of proteins. Critical values were determined using $p \leq 0.05$.

$$Z = \frac{\bar{x} - \mu_0}{\sigma/\sqrt{n}}$$

Binding tests with selected LSM probes

Fluorescence polarization (FP) measurements. Direct FP experiments were measured in 384-well plates (Corning) using Synergy H4 multi-mode reader (BioTek). CFU-labeled peptides **1a-11a** were measured at 50 nm concentration in a buffer containing 20 mM HEPES pH 7.5, 150 mM NaCl, 1 mM $CaCl_2$, 500 µM TCEP and 0.01% Tween 20 and were mixed with increasing amounts of S100A4 or S100B. Experiments were executed in three technical repeats, and average FP signal was fitted by a quadratic binding equation using ProFit.(9) Due to the poor solubility of the peptides, performing competitive FP measurements were not possible.

NMR experiments. NMR spectra were acquired with a Bruker Avance 600 MHz spectrometer equipped with a 5 mm z-gradient CP-TCI triple-resonance cryoprobe. For transferred NOE NMR experiments of S100 proteins, foldamers (**1b**, **12b**) were dissolved in 20 mM, pH 7.0 d_{18}-HEPES (90% H_2O, 10% D_2O), 2 mM $CaCl_2$, 0.2 mM TCEP, containing 0.02% NaN_3. Spectra were acquired at 298 K and excitation sculpting pulse scheme was applied for solvent suppression. Foldamer concentrations varied between 100 µM and 200 µM, depending on the solubility of the peptide. Control 2D NOESY experiments were measured in the absence of the protein with 256 increments and a mixing time of 150 ms. Afterwards, protein was added to the samples and 2D NOESY spectra were measured again. 10-40 µM protein concentrations were used.

In the NMR experiments of **1b** and **12b**, 20 mM, pH 7.0 d_{18}-HEPES (90% H_2O, 10% D_2O), 0.2 mM TCEP, containing 0.02% NaN_3 was used as NMR buffer and spectra were acquired at 298 K. 1D ^1H NMR spectra with 256 scans were acquired for samples containing cca. 25 µM foldamer. Afterwards, Gal-1 was added to the samples in a solid form resulting in a protein concentration of cca. 25 µM, which was equimolar to the foldamers and spectra were measured again.

Results and Discussion

Validation of selected foldamer-protein interactions in solution phase.

In our previous work, the interaction of selected foldamers with CaM was characterized by several methods including tryptophan fluorescence blue-shift measurements, ITC and NMR.(2) The estimated dissociation constants in present work are in good accordance with our previous results corroborating that the method provides valid data for assessing the K_D range of the individual compounds in a foldamer library.

In order to further support the reliability of the pull-down results, selected foldamer peptides were synthetized separately and tested for binding with the proteins. As LSM hits for Gal-1 and RecQ-WH proved to be mainly more promiscuous hydrophobic probes, whose solubility is also limited, we focused on S100A4 and S100B binding helices. 11 compounds were selected, involving peptides from all H14 sublibraries (aromatic, apolar, charged and polar) and were synthetized in carboxyfluorescein-tagged form (compounds **1a-11a**, Fig. S2) and tested for binding to S100A4 and S100B by fluorescence polarization (FP) measurements. Besides LSM probes with good K_D values, peptides with medium affinity were also tested in order to show that they are not false positives. Non-binding LSM probes were also included in the experiments in order to test for false negatives. Moreover, we also aimed to highlight the different behavior of S100A4 and S100B (e.g. S100B binds RR and TW, but S100A4 does not; moreover, S100A4 binds TI and TM, but S100B does not). In general, micromolar binding of the foldamers could be confirmed by FP experiments, however, in several cases, K_D found by fitting of FP curves was higher as estimated by pull-down experiments (Fig. S3a, Fig. S4a). Considering that hexapeptides were tested as CFU-tagged compounds, we assume that CFU changed the size and the character of the compounds, thereby decreasing their affinity to S100A4 and S100B, whose binding partners usually do not abound in aromatic sidechains.

Discrepancy was found in case of two Thr-containing foldamers (**10a, 11a**) whose binding could not be confirmed by FP measurements, however computational modelling of these compounds strongly indicated that these compounds can mimick the recognition segments of NMIIA and p53 when binding to S100A4 and S100B, respectively. On the other hand, a steric clash due to CFU was presumptive. Therefore, non-tagged compounds **10b** and **11b** were synthetized and their binding to S100A4 and S100B were tested by trNOE NMR experiments. In 2D NOESY control spectra of the peptides, no crosspeaks could be detected. By the addition of small amounts of S100A4 or S100B to the samples, crosspeaks appeared in the spectra, which proved the binding of the compounds to the proteins (Fig. S3b, Fig. S4b).

Binding of compounds **1b** and **12b** to Gal-1 could not be depicted by trNOE NMR experiments, but small signal intensity loss of the peptides was observed in the presence of the protein. Therefore, 1D ^{1}H NMR spectra were recorded for the peptides alone and for samples containing the foldamers and Gal-1 in an equimolar ratio. The spectra clearly showed signal attenuation of **1b** and **12b** in the presence of Gal-1 (Fig. S5), which usually indicate a low micromolar to nanomolar binding. On the other hand, pull-down experiments estimated K_Ds of 30-40 µM for these compounds. The surface of Gal-1 is highly hydrophilic and hydrophobic patches, which can bind these compounds, are found at the dimerization interface or between the sheets or within some loops of the protein. Thus, a slow off-rate of the foldamers can be speculated, which can explain the NMR intensity loss and the absence of trNOE even in the case of micromolar binding.

Supplementary Figures and Tables

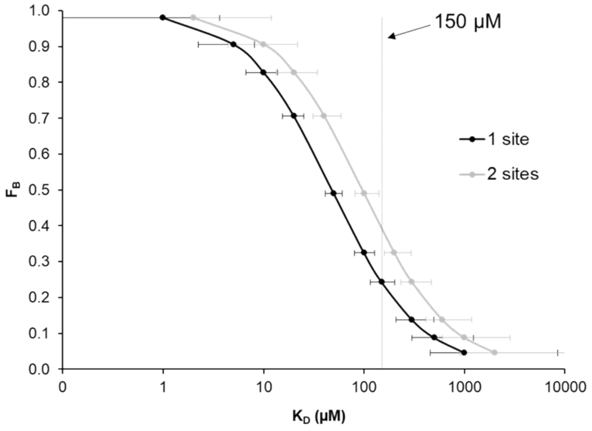

Fig. S1. Useful affinity window of pull-down measurements considering experimental error.

Calculated apparent dissociation constants in the case of 1 binding site (black) or 2 binding site (grey) per protein using $c_{protein}$=64 µM and assuming that 25 % of the compounds within the sublibrary (= 64µM * 0.25 = 16 µM) is bound to the protein, that is c_{free} protein site= 64 µM - 6 µM = 48 µM or 2 * 64µM - 16 µM = 112 µM in the case of 1:1 and 1:2 stoichiometry, respectively. Error bars represent K_D values calculated with F_B+0.05 and F_B-0.05 values as bound fractions, considering the experimental error.

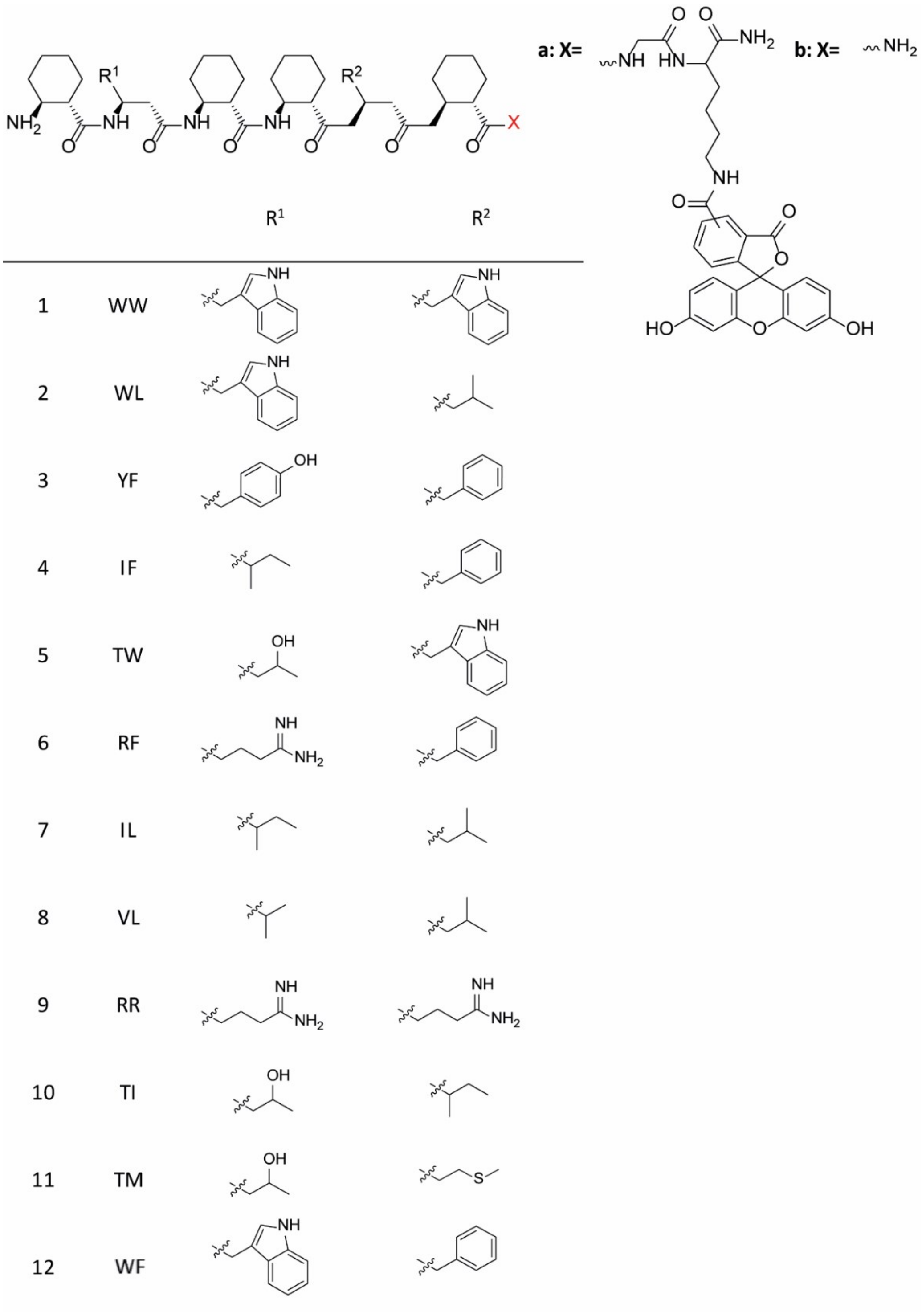

Fig. S2. Structure of the selected H14 LSM probes tested with FP and NMR measurements.

In FP measurements, CFU-tagged compounds **1a-11a** were tested with S100 proteins, while **1b** and **11b** were measured by NMR.

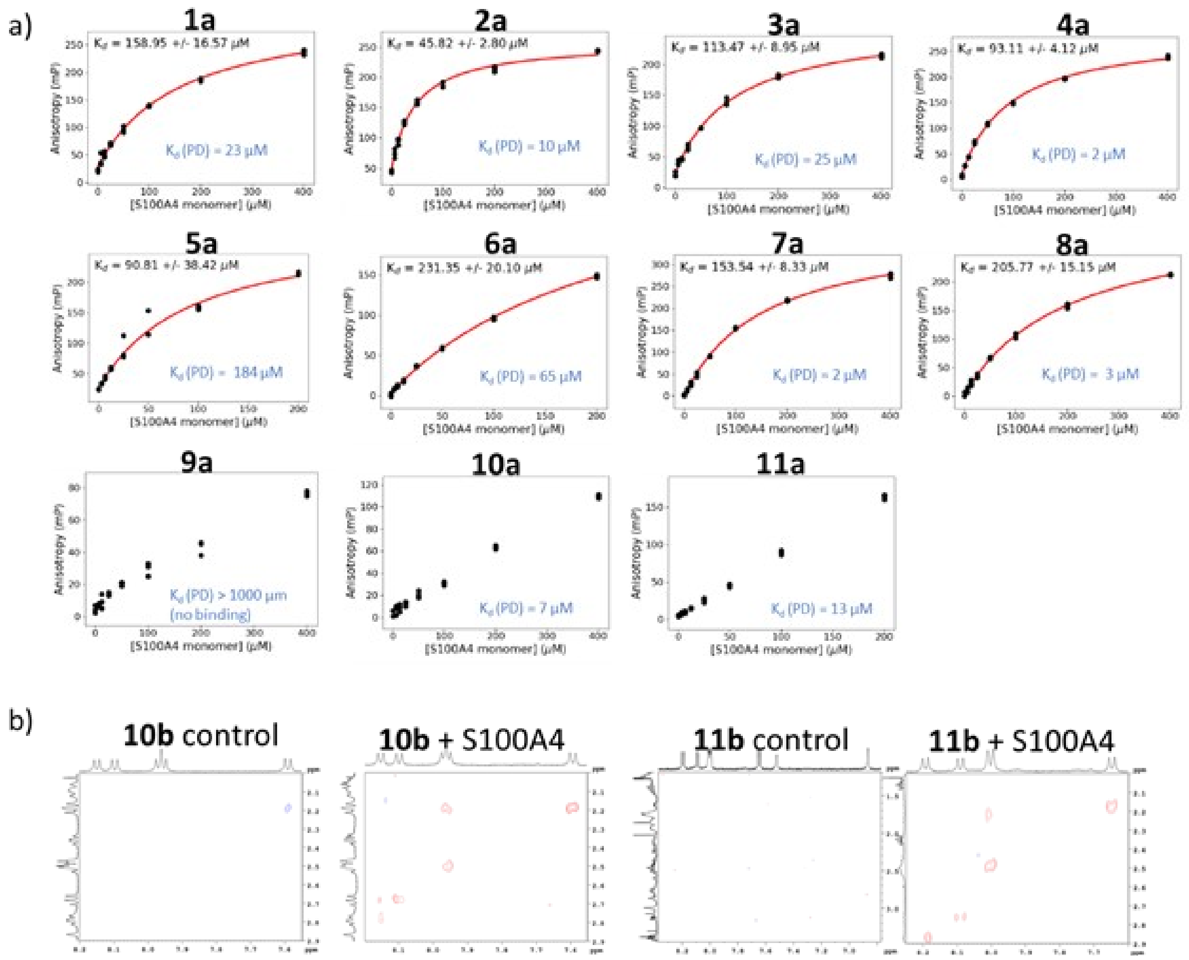

Fig. S3. Independent binding tests for selected foldamer probes and S100A4 protein.

a) FP measurements with CFU-tagged peptides. Upper left K_D values (black) demonstrate the dissociation constants fitted for the FP measurements, while K_D(PD) values (blue) indicate the apparent dissociation constants found in the pull-down experiments. b) TrNOE NMR measurements for compounds **10b** and **11b**. Crosspeaks absent in control spectra but appeared in 2D NOESY spectra in the presence of the protein indicate LSM probe binding to S100A4 protein. Blue and red crosspeaks correspond to negative and positive signals, respectively, relative to diagonal peaks.

Peptides selected for validation cover high (IL, IF, TI, TM, VL, WL), medium (RF, YF, WW) and low (TW, RR) affinity probes to show differences between S100A4 and S100B. Validation of medium and low affinity hits assists excluding false positive and negative hits, respectively.

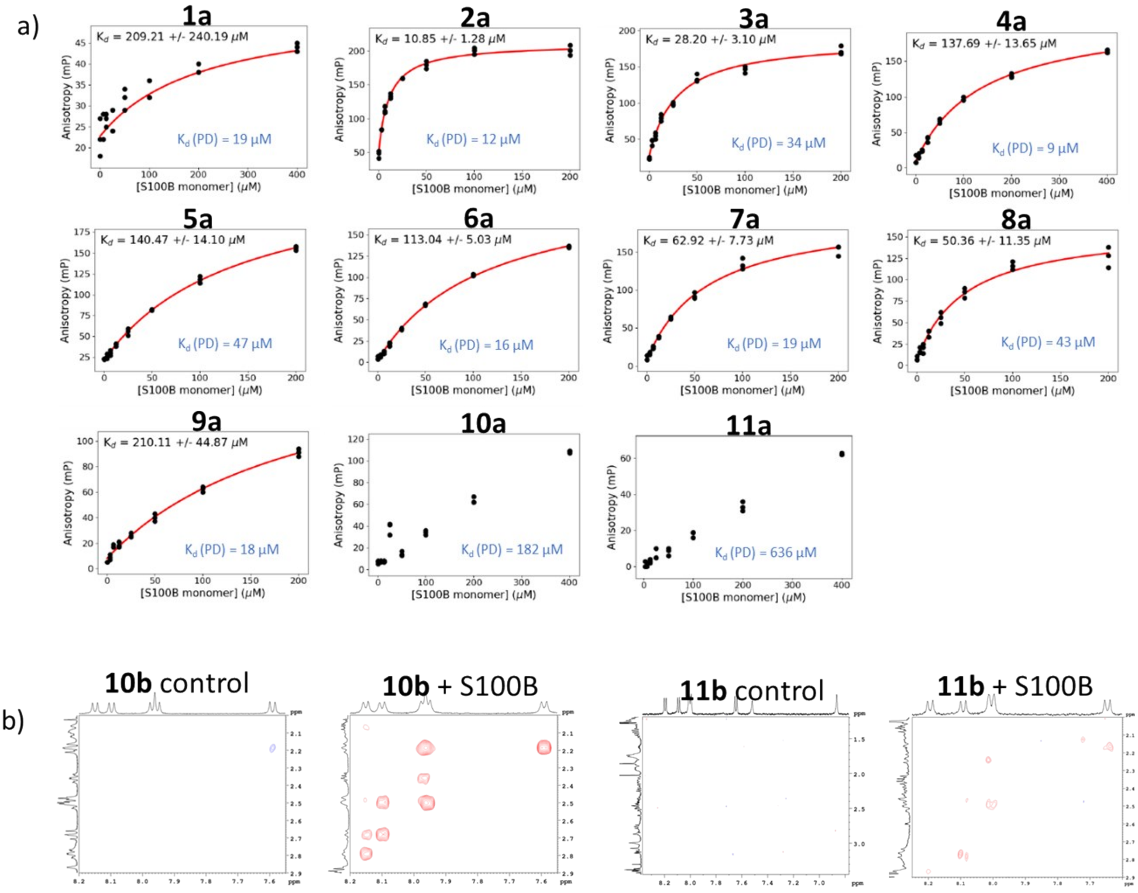

Fig.S4. Independent binding tests for selected foldamer probes and S100AB protein.

a) FP measurements with CFU-tagged peptides. Upper left K_D values (black) demonstrate the dissociation constants fitted for the FP measurements, while K_D(PD) values (blue) indicate the apparent dissociation constants found in the pull-down experiments. b) TrNOE NMR measurements for compounds **10b** and **11b**. Crosspeaks absent in control spectra but appeared in 2D NOESY spectra in the presence of the protein indicate LSM probe binding to S100B protein. Blue and red crosspeaks correspond to negative and positive signals, respectively, relative to diagonal peaks.

Peptides selected for validation cover high (IF, IL, RF, RR, WW, WL), medium (TW, VL, YF) and low (TI, TM) affinity probes to show differences between S100B and S100A4. Validation of medium and low affinity hits assists excluding false positive and negative hits, respectively.

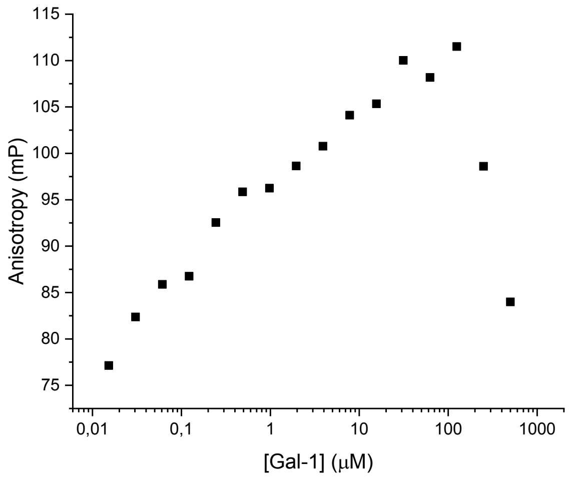

Fig.S5. Independent binding test for compound **1a** and Gal-1 protein.

FP measurement using CFU-tagged peptide **1a**. Full titration could not be achieved due to the known aggregation tendency of Gal-1, therefore, accurate K_D value could not be calculated, but the curve is in agreement with a weak binding.

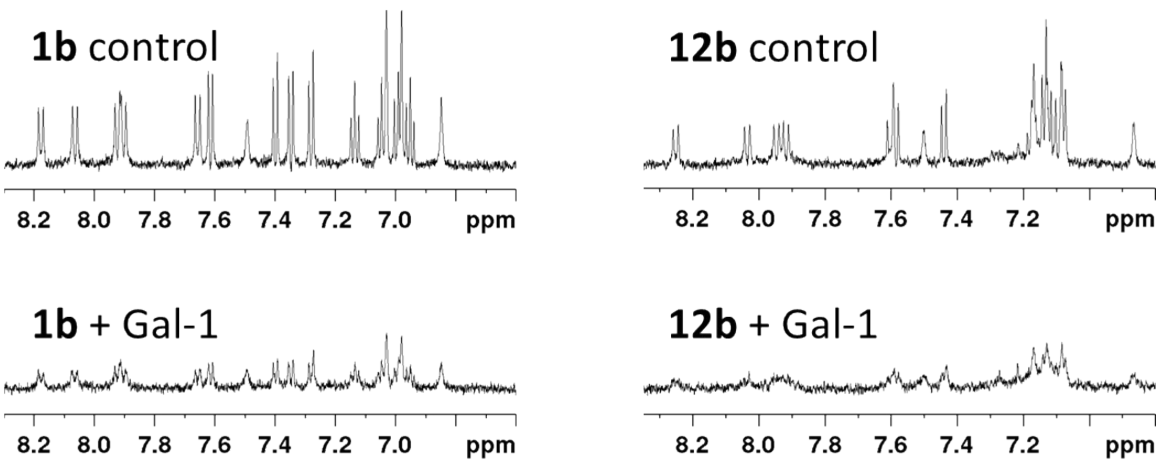

Fig.S6. ^{1}H NMR signal attenuation of **1b** and **12b** compounds in the presence of Gal-1 indicates binding of the foldamers to Gal-1.

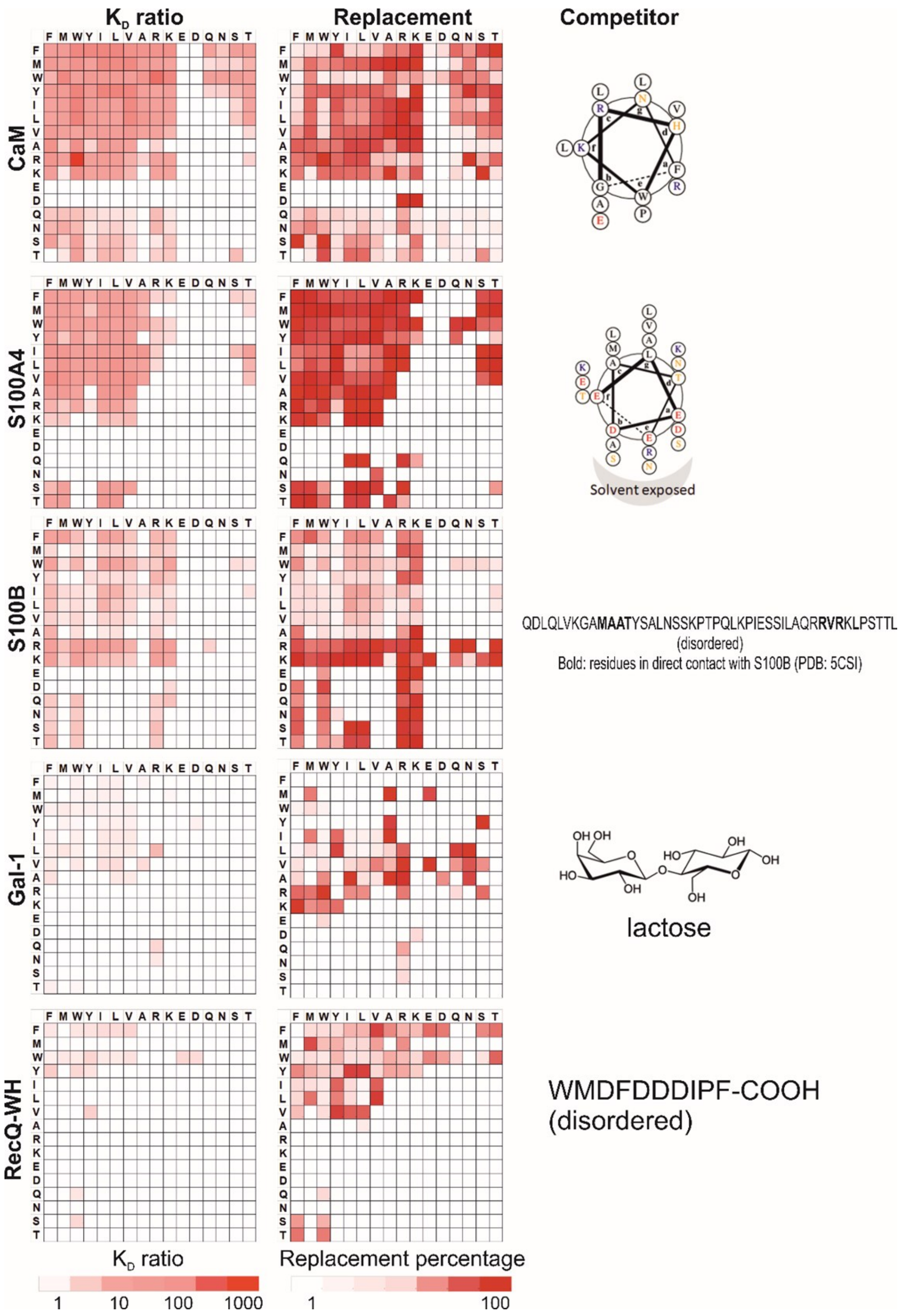

Fig.S7. Competition maps of the H14 libraries based on two different calculations.

Ratio of apparent K_D values obtained from simple H14 pulldowns and from competition experiments highlight the potential orthosteric foldamer hits (left). Percentage of replaced foldamers (middle) is given for LSM probes with F_B>0.1 (≈K_D of 500 µM). Competitor sequences or structures are displayed on the right.

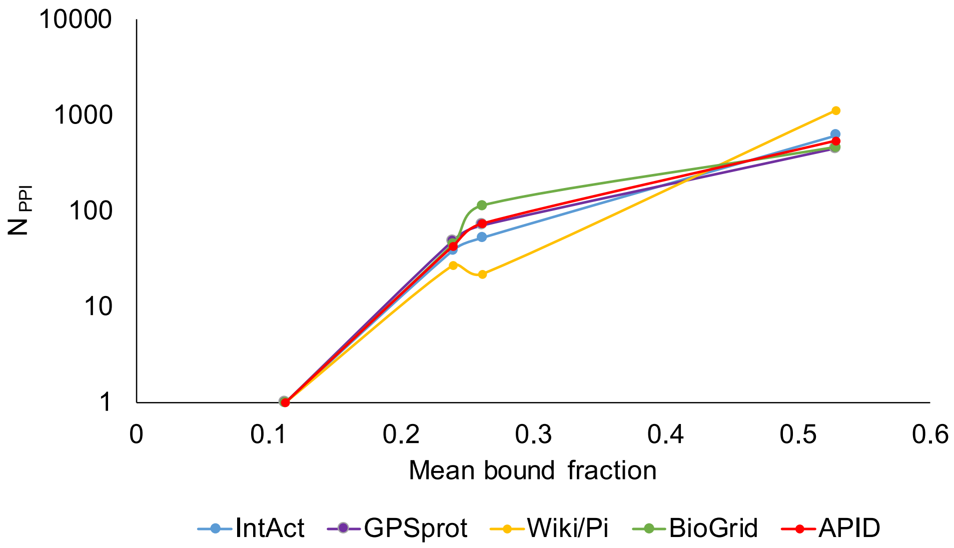

Fig.S8. Average bound fractions for H14 helical LSM library compared with the number of the PPIs found in databases BioGRID, Wiki-Pi, GPS-Prot, IntAct and APID.

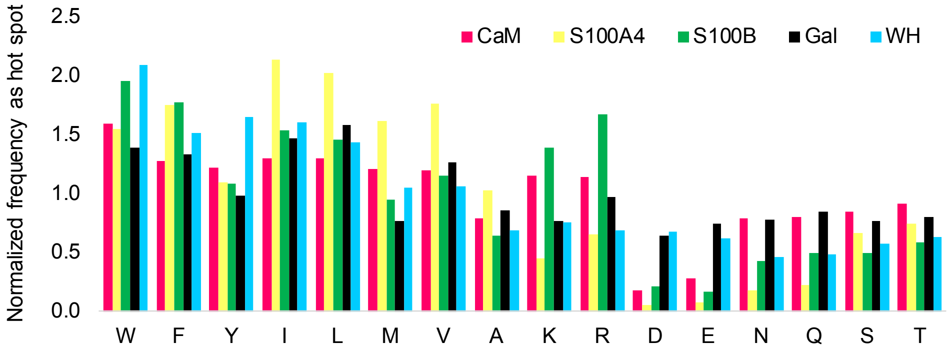

Fig.S9. Normalized residue frequencies as hot spots for different proteins obtained from experimental FB values for H14 LSM libraries.

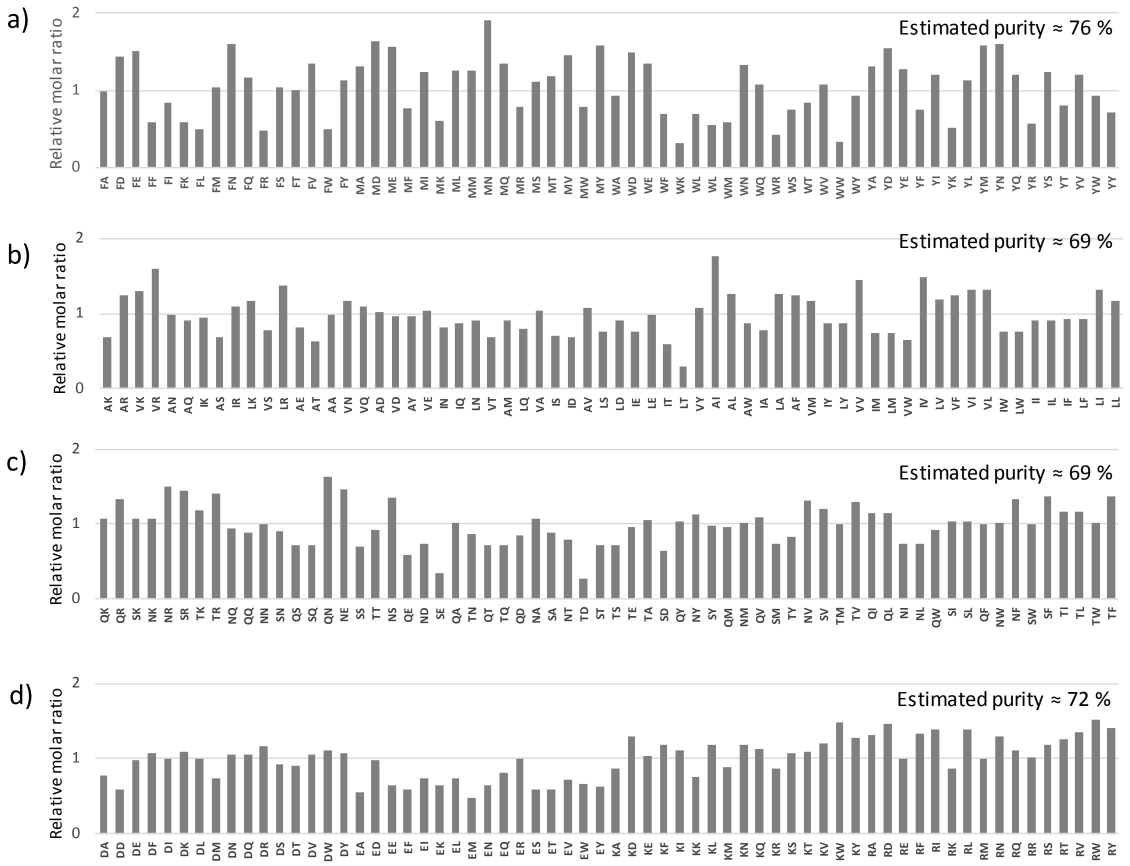

Fig.S10. Estimation of equimolarity and purity of the 64-membered H12 sublibraries.

a) aromatic, b) apolar c) polar d) charged. Estimations were based on peak area integrations of HPLC-MS measurements. Equimolarity was estimated using the following formula: $AUC_{compound}$ / (AUC_{total} /64). The relative value of 1 indicates equimolar concentration. Purity was estimated using the following formula: $AUC_{compound}$ / AUC_{total} *100. Single letter amino acid codes are corresponding to the homologous β^3 amino acid used in position 3 and 6 of the foldamers.

Table S1. Surface mapping K_D results obtained for CaM.

Hot-spot representation can be found in main text Fig. 3 and Fig. 5. Data are given in µM. K_D > 500 µM are marked with star.

H14 library (corresponds to Fig. 3 and Fig.5, CaM, left panel)

	F	M	W	Y	I	L	V	A	R	K	E	D	Q	N	S	T
**F**	1.2	2	0.7	3	0.6	0.6	1.2	3	3	4	475	489	49	90	15	11
**M**	2	16	2	15	3	3	3	16	19	33	*	*	145	122	106	63
**W**	4	1.4	1.2	2	1.0	1.0	2	3	0.5	1.1	191	308	16	14	9	6
**Y**	2	7	2	7	2	2	2	15	11	17	*	*	123	111	67	30
**I**	9	11	0.4	15	9	9	3	16	20	31	*	*	321	281	142	51
**L**	11	14	0.4	10	16	16	3	8	14	27	*	*	275	294	99	39
**V**	5	26	13	30	13	13	7	38	40	67	*	*	*	*	264	142
**A**	5	98	2	119	13	13	42	402	126	181	*	*	*	*	*	*
**R**	4	30	0.6	16	10	10	26	111	10	22	*	*	326	185	155	124
**K**	8	63	3	36	22	22	50	318	22	49	*	*	307	455	262	242
**E**	*	*	*	*	*	*	*	*	*	*	*	*	*	*	*	*
**D**	*	*	*	*	*	*	*	*	*	*	*	*	*	*	*	*
**Q**	39	51	27	92	82	87	94	174	43	96	221	231	186	177	187	211
**N**	45	50	38	109	80	76	115	185	58	82	254	211	190	162	184	187
**S**	30	43	23	90	75	64	101	384	75	91	236	225	176	184	172	215
**T**	163	46	16	84	69	69	113	244	75	88	285	314	156	176	71	253

H12 library (corresponds to Fig. 3, CaM, right panel)

	F	M	W	Y	I	L	V	A	R	K	E	D	Q	N	S	T
**F**	21	42	134	*	15	205	157	496	125	448	*	*	*	*	*	*
**M**	74	*	42	178	101	98	342	*	*	*	*	*	*	*	*	*
**W**	9	42	413	30	134	27	83	303	226	*	*	*	*	*	*	365
**Y**	30	178	30	443	*	*	*	*	*	42	*	*	*	*	*	*
**I**	28	211	19	28	67	67	270	*	236	*	*	*	*	*	*	*
**L**	28	211	19	28	67	67	270	*	236	*	*	*	*	*	*	*
**V**	128	*	56	247	164	164	*	*	*	*	*	*	*	*	*	*
**A**	*	*	234	*	*	*	*	*	*	*	*	*	*	*	*	*
**R**	96	464	19	381	252	252	*	*	123	330	*	*	*	*	*	*
**K**	239	*	51	*	314	324	*	*	330	*	*	*	*	*	*	*
**E**	*	*	*	*	*	*	*	*	*	*	*	*	*	*	*	*
**D**	*	*	*	*	*	*	*	*	*	*	*	*	*	*	*	*
**Q**	181	364	55	403	263	263	*	*	*	*	474	433	*	*	*	447
**N**	162	378	63	364	214	214	460	447	*	*	*	400	*	*	99	*
**S**	153	340	41	351	252	252	299	482	*	*	*	441	*	493	480	*
**T**	90	200	25	132	110	110	208	392	282	*	*	*	447	*	*	*

competition (corresponds to Fig. 5, CaM, right panel)

	F	M	W	Y	I	L	V	A	R	K	E	D	Q	N	S	T
**F**	47	109	118	*	121	121	141	309	290	*	*	*	*	*	*	*
**M**	133	448	208	402	435	435	*	*	*	*	*	278	489	*	431	*
**W**	29	251	35	119	55	55	85	307	167	201	*	*	495	294	307	135
**Y**	119	402	404	*	428	428	448	366	117	*	*	*	*	*	*	*
**I**	102	*	88	*	280	276	*	*	*	*	*	*	*	*	*	*
**L**	162	468	88	455	280	276	*	*	*	*	*	*	*	*	*	*
**V**	239	*	153	*	*	*	*	*	*	*	*	*	*	*	*	*
**A**	*	*	297	*	*	*	*	*	*	*	*	*	*	*	*	*
**R**	350	*	*	*	*	*	219	449	103	249	*	*	*	*	*	*
**K**	339	146	398	*	*	*	376	*	249	*	*	365	*	*	*	*
**E**	*	*	*	*	*	*	492	*	*	*	*	*	*	*	*	*
**D**	220	322	439	*	*	*	*	*	*	*	*	447	212	*	*	*
**Q**	211	317	168	345	241	241	229	211	291	208	246	349	295	304	336	266
**N**	339	452	269	270	283	345	261	399	434	254	414	471	222	290	279	336
**S**	*	210	*	244	471	436	398	362	174	*	232	*	292	279	236	307
**T**	94	113	*	226	*	*	376	398	338	485	281	232	269	292	487	416

Table S2. Surface mapping K_D results obtained for S100A4.

Hot-spot representation can be found in main text Fig. 3 and Fig. 5. Data are given in µM. $K_D > 500$ µM are marked with star.

H14 library (corresponds to Fig. 3 and Fig.5, S100A4, left panel)

	F	M	W	Y	I	L	V	A	R	K	E	D	Q	N	S	T
**F**	12	22	24	57	9	9	12	58	124	141	*	*	377	337	111	135
**M**	23	14	41	90	11	11	21	72	247	339	*	423	476	*	320	148
**W**	10	43	23	35	10	10	13	55	81	136	428	*	191	224	176	157
**Y**	25	90	25	70	19	19	23	217	117	146	371	*	227	435	346	222
**I**	2	3	8	20	2	2	3	7	103	307	*	*	319	321	79	19
**L**	3	4	8	26	2	1	5	34	138	312	427	*	304	433	195	66
**V**	3	5	11	50	3	3	3	35	284	*	*	*	*	*	263	79
**A**	13	10	42	221	7	7	6	205	*	*	*	*	*	*	*	356
**R**	65	94	56	112	51	51	72	*	*	*	*	*	*	*	*	*
**K**	102	95	184	*	85	85	71	*	*	*	*	*	*	*	*	*
**E**	*	*	*	*	*	*	*	*	*	*	*	*	*	*	*	*
**D**	*	*	*	*	*	*	*	*	*	*	*	*	*	*	*	*
**Q**	443	448	*	*	256	256	*	*	344	382	*	*	*	*	*	*
**N**	*	*	*	*	472	472	395	*	*	*	*	*	*	*	*	*
**S**	71	26	179	*	19	19	34	*	188	*	*	*	*	*	*	323
**T**	43	13	189	*	7	7	*	338	407	*	*	*	*	*	*	*

H12 library (corresponds to Fig. 3, S100A4, right panel)

	F	M	W	Y	I	L	V	A	R	K	E	D	Q	N	S	T
**F**	125	197	75	192	126	83	207	*	*	*	*	*	*	*	*	*
**M**	303	*	170	*	358	245	*	*	*	*	*	*	*	*	*	*
**W**	135	98	56	153	164	105	183	*	326	*	*	*	*	*	*	*
**Y**	239	*	153	*	283	180	*	*	*	*	*	*	*	*	*	*
**I**	32	381	28	100	79	79	373	*	*	*	*	*	*	*	*	*
**L**	32	381	28	100	143	131	*	*	345	*	*	*	*	*	*	*
**V**	95	*	184	392	190	190	*	*	*	*	*	*	497	*	*	*
**A**	*	*	*	*	*	*	*	*	*	*	*	*	*	*	*	*
**R**	140	335	95	400	245	245	422	*	210	*	*	*	*	*	*	*
**K**	416	*	456	*	*	89	*	*	*	*	*	*	*	*	*	*
**E**	*	*	*	*	*	*	*	*	*	*	*	*	*	*	*	*
**D**	*	*	*	*	449	449	*	*	*	*	*	*	*	*	*	*
**Q**	*	*	*	*	*	*	*	*	*	*	*	*	*	*	*	*
**N**	*	*	*	*	*	*	*	*	245	*	*	*	*	*	230	*
**S**	*	*	*	*	*	*	*	*	*	*	*	*	*	*	*	*
**T**	*	*	*	*	*	*	*	*	*	*	*	*	*	*	*	*

competition (corresponds to Fig. 5, S100A4, right panel)

	F	M	W	Y	I	L	V	A	R	K	E	D	Q	N	S	T
**F**	*	*	440	*	280	280	376	*	*	*	*	*	*	*	*	*
**M**	*	*	*	*	447	447	*	*	*	*	*	*	*	*	*	*
**W**	*	*	462	367	255	255	*	*	*	*	*	*	*	*	*	*
**Y**	*	*	391	*	*	*	*	*	*	*	*	*	*	*	*	*
**I**	162	217	244	*	101	101	187	*	*	*	*	*	*	*	*	*
**L**	140	318	244	*	101	117	192	*	*	*	*	*	*	*	*	*
**V**	474	*	*	*	246	246	356	*	*	*	*	*	*	*	*	*
**A**	*	*	*	*	*	*	*	*	*	*	*	*	*	*	*	*
**R**	*	*	*	324	*	*	*	*	*	*	*	*	*	*	*	*
**K**	*	*	*	*	*	*	*	*	*	*	*	*	*	*	*	*
**E**	*	*	*	*	*	*	*	*	*	*	*	*	*	*	*	*
**D**	*	*	*	*	*	*	*	*	*	*	*	*	*	*	*	*
**Q**	*	*	*	*	*	*	*	*	*	*	*	*	*	*	*	*
**N**	*	*	*	*	*	*	*	*	*	*	*	*	*	*	*	*
**S**	*	*	*	*	*	*	*	*	*	*	*	*	*	*	*	*
**T**	*	*	*	*	473	473	*	*	*	*	*	*	*	*	*	*

Table S3. Surface mapping K_D results obtained for S100B.

Hot-spot representation can be found in main text Fig. 3 and Fig. 5. Data are given in µM. K_D > 500 µM are marked with star.

H14 library (corresponds to Fig. 3 and Fig. 5, S100B, left panel)

	F	M	W	Y	I	L	V	A	R	K	E	D	Q	N	S	T
**F**	16	19	27	96	21	21	44	253	56	63	*	*	433	*	364	274
**M**	85	174	44	176	62	62	118	380	92	125	*	*	*	*	*	476
**W**	14	45	19	66	12	12	16	58	32	40	*	*	161	168	138	107
**Y**	34	176	42	121	62	62	112	*	67	89	*	*	480	*	*	416
**I**	9	47	21	74	19	19	43	108	37	84	*	*	497	*	382	142
**L**	11	67	21	85	19	28	56	170	82	82	*	*	435	409	379	162
**V**	20	93	16	110	43	43	73	299	77	138	*	*	*	*	*	382
**A**	45	256	42	192	108	108	153	*	126	180	*	*	*	*	*	*
**R**	16	79	13	40	31	31	63	188	18	22	*	*	116	212	413	324
**K**	26	119	28	63	80	80	102	319	22	53	324	*	173	195	217	194
**E**	476	*	305	*	*	*	*	*	349	324	*	*	*	*	*	*
**D**	339	*	200	461	*	*	*	*	193	144	*	*	*	*	*	*
**Q**	83	*	79	166	*	*	*	*	107	81	*	*	*	*	*	*
**N**	123	*	98	193	*	*	*	*	128	270	*	*	*	*	*	*
**S**	105	*	79	225	380	380	*	*	76	181	*	*	*	*	*	*
**T**	44	*	47	180	183	183	*	*	94	172	*	*	*	*	*	*

H12 library (corresponds to Fig. 3, S100B, right panel)

	F	M	W	Y	I	L	V	A	R	K	E	D	Q	N	S	T
**F**	14	39	10	59	13	14	46	116	28	37	391	371	104	109	310	86
**M**	32	166	26	102	54	81	127	224	59	78	417	471	192	177	215	162
**W**	7	20	8	28	18	21	38	60	15	26	229	199	53	55	54	56
**Y**	36	102	28	128	64	81	166	95	65	86	459	430	222	237	195	251
**I**	22	74	11	56	25	25	57	254	39	87	*	*	*	*	360	216
**L**	22	74	11	56	37	46	116	293	29	54	*	*	348	264	258	216
**V**	45	303	31	210	92	92	277	*	118	185	*	*	*	*	*	*
**A**	114	*	62	318	348	348	*	*	219	380	*	*	*	*	*	*
**R**	40	96	25	89	56	56	133	205	89	146	*	*	210	217	*	133
**K**	66	153	39	138	71	68	193	243	146	177	*	149	300	274	224	226
**E**	*	*	*	*	*	*	*	*	*	*	*	*	*	*	*	*
**D**	*	*	*	*	*	*	*	*	*	*	*	*	*	*	*	*
**Q**	461	*	170	*	*	*	*	*	321	*	*	*	*	*	*	*
**N**	368	*	155	*	*	*	*	*	106	*	*	*	*	*	*	*
**S**	189	*	114	*	419	419	*	*	123	401	*	*	*	*	*	*
**T**	185	*	113	*	*	*	*	*	286	253	*	*	*	*	*	*

competition (corresponds to Fig.5, S100B, right panel)

	F	M	W	Y	I	L	V	A	R	K	E	D	Q	N	S	T
**F**	180	352	177	146	202	202	253	330	406	391	*	*	494	*	429	333
**M**	316	276	131	330	269	269	360	382	*	*	*	*	394	449	391	337
**W**	177	125	138	104	123	123	116	220	166	412	*	*	*	*	434	242
**Y**	149	344	38	393	201	201	372	436	*	*	*	*	449	476	399	403
**I**	64	163	67	156	147	147	178	296	174	216	*	*	387	325	456	373
**L**	71	163	67	154	147	147	213	294	167	316	*	*	*	254	371	294
**V**	91	229	60	167	181	180	198	393	221	390	369	*	339	271	460	484
**A**	354	359	127	304	238	313	316	*	*	*	*	*	*	*	*	*
**R**	*	*	*	*	*	*	*	*	*	*	*	*	*	*	*	*
**K**	*	140	*	*	*	*	*	*	*	*	*	*	*	*	*	*
**E**	126	141	*	*	*	*	*	*	*	*	*	*	*	*	*	*
**D**	130	159	*	*	*	*	*	*	*	*	*	*	*	*	*	*
**Q**	425	*	473	188	*	*	*	*	*	*	*	*	*	*	*	*
**N**	*	*	*	444	*	*	*	*	*	*	*	*	478	120	*	*
**S**	*	*	458	109	*	*	*	*	*	*	*	*	445	*	*	*
**T**	294	*	258	*	*	*	*	*	*	*	*	*	*	445	*	*

Table S4. Surface mapping K_D results obtained for RecQ-WH.

Hot-spot representation can be found in main text Fig. 3 and Fig. 5. Data are given in µM. K_D > 500 µM are marked with star.

H14 library (corresponds to Fig. 3 and Fig. 5, RecQ-WH, left panel)

	F	M	W	Y	I	L	V	A	R	K	E	D	Q	N	S	T
**F**	134	225	63	92	79	79	129	282	447	424	328	193	*	*	303	218
**M**	304	455	197	263	226	226	294	*	457	*	*	*	*	*	*	*
**W**	62	81	40	50	68	68	102	293	272	287	137	145	473	*	209	165
**Y**	84	263	46	114	150	150	212	329	421	411	*	*	*	*	*	*
**I**	186	340	195	300	160	206	366	*	*	*	*	*	*	*	*	*
**L**	186	233	195	239	160	206	177	*	*	*	*	*	*	*	*	*
**V**	347	*	221	115	366	366	*	*	*	*	*	*	*	*	*	*
**A**	483	*	*	*	*	401	*	*	*	*	*	*	*	*	*	*
**R**	231	294	201	*	237	237	*	456	355	486	*	427	*	439	403	*
**K**	*	459	*	407	*	*	442	*	486	*	*	*	*	*	*	*
**E**	241	*	212	*	444	444	*	*	*	*	*	*	*	*	*	*
**D**	288	*	165	*	429	429	*	*	*	314	*	*	*	*	*	*
**Q**	*	*	143	*	*	*	*	*	*	*	*	*	*	*	*	*
**N**	*	*	*	*	*	*	*	*	*	*	*	*	*	*	*	*
**S**	182	*	73	*	*	*	*	*	*	*	*	*	*	*	*	*
**T**	407	*	288	*	*	*	*	*	*	*	*	*	*	*	*	*

H12 library (corresponds to Fig. 3, RecQ-WH, right panel)

	F	M	W	Y	I	L	V	A	R	K	E	D	Q	N	S	T
**F**	*	*	*	*	*	*	*	*	388	337	*	*	*	*	*	*
**M**	*	*	*	*	*	*	*	*	435	*	*	*	*	*	*	*
**W**	*	*	*	*	*	*	*	*	206	431	*	*	*	*	420	*
**Y**	*	*	*	*	*	*	*	*	*	280	*	*	*	*	*	*
**I**	*	*	*	*	*	*	*	*	*	*	*	*	*	*	*	*
**L**	*	*	*	*	*	*	*	*	*	434	*	*	*	*	*	*
**V**	*	*	*	*	*	*	*	*	*	*	*	*	*	*	*	*
**A**	*	*	*	*	*	*	*	*	*	*	*	*	*	*	*	*
**R**	*	*	*	*	*	*	*	*	*	*	*	*	*	*	*	*
**K**	*	*	*	*	*	*	*	*	*	*	*	*	*	*	*	*
**E**	*	*	*	*	*	*	*	*	*	*	*	*	*	*	*	*
**D**	*	*	*	*	*	*	*	*	*	*	*	*	*	*	*	*
**Q**	*	*	*	*	*	*	*	*	*	*	*	*	*	*	*	*
**N**	*	*	*	*	*	*	*	*	*	*	*	*	*	*	*	*
**S**	*	*	*	*	*	*	*	*	*	*	*	*	*	*	*	*
**T**	*	*	*	*	*	*	*	*	*	*	*	*	*	*	*	*

competition (corresponds to Fig.5, RecQ-WH, right panel)

	F	M	W	Y	I	L	V	A	R	K	E	D	Q	N	S	T
**F**	312	454	153	231	266	266	*	*	*	*	*	*	*	*	*	*
**M**	*	*	*	*	*	*	*	*	*	*	*	*	*	*	*	*
**W**	106	188	84	193	142	142	268	*	*	*	*	479	*	*	352	*
**Y**	400	*	164	298	*	*	472	*	*	*	*	*	*	*	*	*
**I**	439	*	315	*	364	266	*	*	*	*	*	*	*	*	*	*
**L**	439	*	315	*	355	253	*	*	*	*	*	*	*	*	*	*
**V**	*	*	452	*	*	*	*	*	*	*	*	*	*	*	*	*
**A**	454	*	*	*	*	*	*	*	*	*	*	*	*	*	*	*
**R**	*	*	*	*	*	*	*	*	*	*	*	*	*	*	*	*
**K**	*	*	*	*	*	*	*	*	*	*	*	*	*	*	*	*
**E**	*	*	*	*	*	*	*	*	*	*	*	*	*	*	*	*
**D**	*	*	*	*	*	*	*	*	*	*	*	*	*	*	*	*
**Q**	*	*	341	*	*	*	*	*	*	*	*	*	*	*	*	*
**N**	*	*	*	*	*	*	*	*	*	*	*	*	*	*	*	*
**S**	*	*	265	*	*	*	*	*	*	*	*	*	*	*	*	*
**T**	*	*	*	*	*	*	*	*	*	*	*	*	*	*	*	*

Table S5. Surface mapping K_D results obtained for Gal-1.

Hot-spot representation can be found in main text Fig. 3 and Fig. 5. Data are given in µM. K_D > 500 µM are marked with star.

H14 library (corresponds to Fig. 3 and Fig. 5, Gal-1, left panel)

	F	M	W	Y	I	L	V	A	R	K	E	D	Q	N	S	T
**F**	75	284	81	214	102	102	199	130	222	154	*	*	*	*	*	348
**M**	223	183	141	98	145	145	178	385	395	133	250	*	*	399	*	424
**W**	38	145	30	145	71	71	143	*	278	262	*	*	*	*	467	*
**Y**	162	98	115	375	148	148	140	342	377	*	*	119	238	313	336	396
**I**	33	238	38	258	24	24	57	247	*	*	*	*	*	*	266	185
**L**	25	138	38	231	24	17	68	160	116	*	463	*	343	314	498	336
**V**	93	*	98	318	105	66	244	114	289	296	313	*	161	302	190	347
**A**	121	307	203	353	248	127	100	326	348	*	*	168	*	303	*	443
**R**	*	*	*	*	*	*	*	*	*	*	*	*	*	*	*	*
**K**	*	*	*	*	*	*	*	*	*	*	*	*	*	*	*	*
**E**	*	*	*	*	*	*	*	*	*	*	*	*	*	*	*	*
**D**	*	*	*	*	*	*	*	*	*	*	*	*	*	*	*	*
**Q**	285	364	169	174	456	457	349	349	71	158	295	379	273	257	350	325
**N**	264	350	169	210	258	259	349	*	114	*	378	229	257	246	310	350
**S**	267	356	151	318	336	251	221	389	156	438	326	432	275	310	435	*
**T**	140	*	143	297	175	175	399	425	372	*	244	326	369	275	408	366

H12 library ((corresponds to Fig. 3, Gal-1, right panel)

	F	M	W	Y	I	L	V	A	R	K	E	D	Q	N	S	T
**F**	130	*	136	*	167	114	170	341	141	246	198	251	208	226	268	254
**M**	*	364	*	286	281	*	247	194	186	172	180	169	173	195	224	169
**W**	117	*	151	*	174	200	*	385	*	266	256	297	304	270	413	448
**Y**	*	286	*	243	319	402	364	222	61	371	198	205	176	181	194	300
**I**	376	378	368	347	395	395	463	469	120	*	*	462	446	401	485	*
**L**	376	378	368	347	415	415	468	438	120	*	335	470	446	423	*	*
**V**	388	459	346	413	469	469	439	*	234	430	469	480	436	497	408	432
**A**	428	*	355	422	436	436	491	*	*	303	388	438	456	330	307	*
**R**	202	201	126	198	194	194	226	485	181	231	*	250	*	*	242	213
**K**	292	282	161	227	250	316	221	*	231	230	212	289	293	*	298	*
**E**	245	251	235	260	244	244	316	340	*	200	349	238	236	*	289	372
**D**	254	218	240	205	249	249	270	248	*	173	238	372	244	226	199	398
**Q**	*	*	412	*	*	*	*	*	431	*	*	*	*	*	*	*
**N**	*	*	*	*	*	*	*	*	*	430	*	*	*	*	*	*
**S**	*	*	459	*	*	*	*	*	402	*	*	*	*	*	*	*
**T**	*	*	*	*	*	*	*	*	*	*	*	*	*	*	*	*

competition (corresponds to Fig.5, Gal-1, right panel)

	F	M	W	Y	I	L	V	A	R	K	E	D	Q	N	S	T
**F**	108	250	122	395	164	164	323	151	208	153	*	*	*	*	*	*
**M**	181	*	172	133	235	235	218	*	310	149	*	*	*	495	*	*
**W**	59	305	51	222	105	105	224	*	280	252	*	*	*	*	*	*
**Y**	234	133	112	*	256	256	223	*	309	*	*	177	439	271	*	*
**I**	53	*	65	*	50	50	114	*	*	*	*	*	*	*	*	333
**L**	43	334	65	*	50	32	128	425	*	*	*	*	*	*	*	*
**V**	193	*	211	*	293	220	*	375	*	*	*	*	*	*	*	*
**A**	333	487	*	*	*	272	201	*	*	*	*	*	*	*	*	416
**R**	*	*	*	*	*	*	418	*	*	323	*	*	421	*	*	*
**K**	*	*	*	*	*	*	*	465	323	298	*	290	*	278	*	370
**E**	430	*	458	*	*	*	300	*	*	*	353	318	443	453	282	*
**D**	388	397	*	*	408	408	316	*	*	405	318	*	*	*	415	282
**Q**	319	406	183	350	486	458	372	444	272	146	380	*	467	*	500	*
**N**	250	406	185	337	323	338	378	*	247	342	*	294	*	444	427	500
**S**	312	352	177	348	478	325	476	432	349	*	415	*	460	427	*	413
**T**	220	*	147	338	266	266	424	426	432	*	366	415	*	460	390	431

Table S6. One-sample one-tailed Z-scores for the "normalized frequency as hot spot" values of the H14 LSM library side-chains.

Z-scores marked with an asterisk represent statistically significant ($p<0.05$) depletion and enrichment, respectively, as revealed by left- and right-handed Z-tests. The critical values for one-tailed Z-test were calculated to be 4 and -4.

	W	F	Y	I	L	M	V	A	K	R	D	E	N	Q	S	T
CaM	1.59	1.28	1.22	1.30	1.29	1.21	1.20	0.79	1.16	1.14	0.18	0.28	0.79	0.80	0.85	0.91
S100A4	1.55	1.75	1.09	2.13	2.02	1.62	1.77	1.02	0.45	0.65	0.06	0.08	0.18	0.22	0.67	0.74
S100B	1.96	1.77	1.08	1.54	1.46	0.95	1.15	0.65	1.39	1.67	0.21	0.17	0.42	0.49	0.49	0.59
Gal	1.39	1.34	0.99	1.47	1.58	0.77	1.27	0.86	0.77	0.97	0.64	0.74	0.78	0.84	0.77	0.80
WH	2.09	1.52	1.65	1.61	1.43	1.05	1.06	0.69	0.76	0.68	0.67	0.62	0.47	0.48	0.58	0.63
$\bar{x}$	1.72	1.53	1.21	1.61	1.56	1.12	1.29	0.80	0.91	1.02	0.35	0.38	0.53	0.57	0.67	0.74
s	0.30	0.23	0.26	0.31	0.28	0.32	0.28	0.15	0.37	0.41	0.28	0.29	0.26	0.26	0.14	0.13
Z-score	5.44*	5.22*	1.76	4.37*	4.48*	0.83	2.32	-2.94	-0.58	0.12	-5.09*	-4.8*	-4.1*	-3.73	-5.16*	-4.53*

Table S7. Characterization data of H12 aromatic sublibrary.

β3-amino		Detected peaks			MS2 fragment ions			Pulldown assay	
3rd	6th	$[M+H^+]^{1+}$	$[M+2H^+]^{2+}$	Rt (min)	b5	y5	y6	Detected ion	Remark
F	A	930.66	466.33	12.89	606.40	547.42	708.50	$[M+H^+]^{1+}$	
F	D	974.65	488.33	12.39	606.40	591.41	752.49	$[M+H^+]^{1+}$	
F	E	988.66	495.33	12.48	606.40	605.42	766.50	$[M+H^+]^{1+}$	
F	F	1006.68	504.34	15.19	606.40	623.44	784.52	$[M+H^+]^{1+}$	
F	I	972.70	487.35	15.14	606.40	589.46	750.54	$[M+H^+]^{1+}$	
F	K	987.71	494.86	10.53	606.40	604.47	765.55	$[M+2H^+]^{2+}$	
F	L	972.70	487.35	15.32	606.40	589.46	750.54	$[M+H^+]^{1+}$	
F	M	990.66	496.33	14.19	606.40	607.42	768.50	$[M+H^+]^{1+}$	
F	N	973.66	487.83	12.02	606.40	590.42	751.50	$[M+H^+]^{1+}$	
F	Q	987.67	494.84	12.08	606.40	604.43	765.51	$[M+H^+]^{1+}$	
F	R	1015.72	508.86	10.60	606.40	632.48	793.56	$[M+2H^+]^{2+}$	
F	S	946.65	474.33	11.39	606.40	563.41	724.49	$[M+H^+]^{1+}$	
F	T	960.66	481.33	12.81	606.40	577.42	738.50	$[M+H^+]^{1+}$	
F	V	958.68	480.34	14.53	606.40	575.44	736.52	$[M+H^+]^{1+}$	
F	W	1045.69	523.85	15.04	606.40	662.45	823.53	$[M+H^+]^{1+}$	
F	Y	1022.68	512.34	13.58	606.40	639.44	800.52	$[M+H^+]^{1+}$	
M	A	914.64	458.32	11.83	590.38	547.42	692.48	$[M+H^+]^{1+}$	
M	D	958.63	480.32	11.43	590.38	591.41	736.47	$[M+H^+]^{1+}$	
M	E	972.64	487.32	11.57	590.38	605.42	750.48	$[M+H^+]^{1+}$	
M	F	990.66	496.33	14.10	590.38	623.44	768.50	$[M+H^+]^{1+}$	
M	I	956.68	479.34	14.07	590.38	589.46	734.52	$[M+H^+]^{1+}$	
M	K	971.69	486.85	9.76	590.38	604.47	749.53	$[M+2H^+]^{2+}$	
M	L	956.68	479.34	14.19	590.38	589.46	734.52	$[M+H^+]^{1+}$	
M	M	974.64	488.32	13.05	590.38	607.42	752.48	$[M+H^+]^{1+}$	
M	N	957.64	479.82	11.06	590.38	590.42	735.48	$[M+H^+]^{1+}$	
M	Q	971.65	486.83	11.16	590.38	604.43	749.49	$[M+H^+]^{1+}$	
M	R	999.70	500.85	9.83	590.38	632.48	777.54	$[M+2H^+]^{2+}$	
M	S	930.63	466.32	11.35	590.38	563.41	708.47	$[M+H^+]^{1+}$	
M	T	944.64	473.32	11.79	590.38	577.42	722.48	$[M+H^+]^{1+}$	
M	V	942.66	472.33	13.37	590.38	575.44	720.50	$[M+H^+]^{1+}$	
M	W	1029.67	515.84	13.90	590.38	662.45	807.51	$[M+H^+]^{1+}$	
M	Y	1006.66	504.33	12.50	590.38	639.44	784.50	$[M+H^+]^{1+}$	average integral of MY, YM
W	A	969.67	485.84	12.89	645.41	547.42	747.51	$[M+H^+]^{1+}$	
W	D	1013.66	507.83	12.44	645.41	591.41	791.50	$[M+H^+]^{1+}$	
W	E	1027.67	514.84	12.55	645.41	605.42	805.51	$[M+H^+]^{1+}$	
W	F	1045.69	523.85	14.85	645.41	623.44	823.53	$[M+H^+]^{1+}$	
W	I	1011.71	506.86	15.01	645.41	589.46	789.55	$[M+H^+]^{1+}$	
W	K	1026.72	514.36	10.63	645.41	604.47	804.56	$[M+2H^+]^{2+}$	
W	L	1011.71	506.86	15.14	645.41	589.46	789.55	$[M+H^+]^{1+}$	
W	M	1029.67	515.84	14.03	645.41	607.42	807.51	$[M+H^+]^{1+}$	
W	N	1012.67	507.34	12.13	645.41	590.42	790.51	$[M+H^+]^{1+}$	
W	Q	1026.68	514.34	12.17	645.41	604.43	804.52	$[M+H^+]^{1+}$	
W	R	1054.73	528.37	10.68	645.41	632.48	832.57	$[M+2H^+]^{2+}$	
W	S	985.66	493.83	12.39	645.41	563.41	763.50	$[M+H^+]^{1+}$	
W	T	999.67	500.84	12.81	645.41	577.42	777.51	$[M+H^+]^{1+}$	
W	V	997.69	499.85	14.40	645.41	575.44	775.53	$[M+H^+]^{1+}$	
W	W	1084.70	543.35	14.79	645.41	662.45	862.54	$[M+H^+]^{1+}$	
W	Y	1061.69	531.85	13.38	645.41	639.44	839.53	$[M+H^+]^{1+}$	average integral of WY, YW
Y	A	946.66	474.33	12.33	622.40	547.42	724.50	$[M+H^+]^{1+}$	
Y	D	990.65	496.33	11.02	622.40	591.41	768.49	$[M+H^+]^{1+}$	
Y	E	1004.66	503.33	11.14	622.40	605.42	782.50	$[M+H^+]^{1+}$	
Y	F	1022.68	512.34	13.42	622.40	623.44	800.52	$[M+H^+]^{1+}$	
Y	I	988.70	495.35	13.44	622.40	589.46	766.54	$[M+H^+]^{1+}$	
Y	K	1003.71	502.86	9.49	622.40	604.47	781.55	$[M+2H^+]^{2+}$	
Y	L	988.70	495.35	13.60	622.40	589.46	766.54	$[M+H^+]^{1+}$	
Y	M	1006.66	504.33	12.50	622.40	607.42	784.50	$[M+H^+]^{1+}$	average integral of MY, YM
Y	N	989.66	495.83	10.70	622.40	590.42	767.50	$[M+H^+]^{1+}$	
Y	Q	1003.67	502.84	10.77	622.40	604.43	781.51	$[M+H^+]^{1+}$	
Y	R	1031.72	516.86	9.57	622.40	632.48	809.56	$[M+2H^+]^{2+}$	
Y	S	962.65	482.33	10.94	622.40	563.41	740.49	$[M+H^+]^{1+}$	
Y	T	976.66	489.33	11.35	622.40	577.42	754.50	$[M+H^+]^{1+}$	
Y	V	974.68	488.34	12.86	622.40	575.44	752.52	$[M+H^+]^{1+}$	
Y	W	1061.69	531.85	13.38	622.40	662.45	839.53	$[M+H^+]^{1+}$	average integral of WY, YW
Y	Y	1038.68	520.34	12.05	622.40	639.44	816.52	$[M+H^+]^{1+}$	

Table S8. Characterization data of H12 apolar sublibrary.

β3-amino		Detected peaks			MS2 fragment ions			Pulldown assay	
3rd	6th	$[M+H^+]^{1+}$	$[M+2H^+]^{2+}$	Rt (min)	b5	y5	y6	Detected ion	Remark
A	A	854.64	428.32	10.82	530.38	547.42	632.48	$[M+H^+]^{1+}$	
A	D	898.63	450.315	11.45	530.38	591.41	676.47	$[M+H^+]^{1+}$	
A	E	912.64	457.32	10.66	530.38	605.42	690.48	$[M+H^+]^{1+}$	
A	F	930.66	466.33	13.14	530.38	623.44	708.5	$[M+H^+]^{1+}$	
A	I	896.68	449.34	12.8	530.38	589.46	674.52	$[M+H^+]^{1+}$	
A	K	911.69	456.845	9.01	530.38	604.47	689.53	$[M+2H^+]^{2+}$	
A	L	896.68	449.34	12.8	530.38	589.46	674.52	$[M+H^+]^{1+}$	
A	M	914.64	458.32	11.99	530.38	607.42	692.48	$[M+H^+]^{1+}$	
A	N	897.64	449.82	10.19	530.38	590.42	675.48	$[M+H^+]^{1+}$	
A	Q	911.65	456.825	10.28	530.38	604.43	689.49	$[M+H^+]^{1+}$	
A	R	939.7	470.85	9.08	530.38	632.48	717.54	$[M+2H^+]^{2+}$	
A	S	870.63	436.315	10.44	530.38	563.41	648.47	$[M+H^+]^{1+}$	
A	T	884.64	443.32	10.8	530.38	577.42	662.48	$[M+H^+]^{1+}$	
A	V	882.66	442.33	12.25	530.38	575.44	660.5	$[M+H^+]^{1+}$	
A	W	969.67	485.835	12.98	530.38	662.45	747.51	$[M+H^+]^{1+}$	
A	Y	946.66	474.33	11.58	530.38	639.44	724.5	$[M+H^+]^{1+}$	
I	A	896.68	449.34	12.98	572.42	547.42	674.52	$[M+H^+]^{1+}$	
I	D	940.67	471.335	12.22	572.42	591.41	718.51	$[M+H^+]^{1+}$	
I	E	954.68	478.34	12.38	572.42	605.42	732.52	$[M+H^+]^{1+}$	
I	F	972.7	487.35	15.3	572.42	623.44	750.54	$[M+H^+]^{1+}$	average integral of IF, LF
I	I	938.72	470.36	15.24	572.42	589.46	716.56	$[M+H^+]^{1+}$	average integral of II, IL
I	K	953.73	477.865	10.37	572.42	604.47	731.57	$[M+2H^+]^{2+}$	
I	L	938.72	470.36	15.24	572.42	589.46	716.56	$[M+H^+]^{1+}$	average integral of II, IL
I	M	956.68	479.34	14.13	572.42	607.42	734.52	$[M+H^+]^{1+}$	average integral of IM, LM
I	N	939.68	470.84	11.82	572.42	590.42	717.52	$[M+H^+]^{1+}$	
I	Q	953.69	477.845	11.88	572.42	604.43	731.53	$[M+H^+]^{1+}$	
I	R	981.74	491.87	10.45	572.42	632.48	759.58	$[M+2H^+]^{2+}$	
I	S	912.67	457.335	12.14	572.42	563.41	690.51	$[M+H^+]^{1+}$	
I	T	926.68	464.34	12.72	572.42	577.42	704.52	$[M+H^+]^{1+}$	average integral of IT, LT
I	V	924.7	463.35	14.43	572.42	575.44	702.54	$[M+H^+]^{1+}$	
I	W	1011.71	506.855	14.87	572.42	662.45	789.55	$[M+H^+]^{1+}$	average integral of IW, LW
I	Y	988.7	495.35	13.45	572.42	639.44	766.54	$[M+H^+]^{1+}$	average integral of IY, LY
L	A	896.68	449.34	13.05	572.42	547.42	674.52	$[M+H^+]^{1+}$	
L	D	940.67	471.335	12.33	572.42	591.41	718.51	$[M+H^+]^{1+}$	
L	E	954.68	478.34	12.48	572.42	605.42	732.52	$[M+H^+]^{1+}$	
L	F	972.7	487.35	15.3	572.42	623.44	750.54	$[M+H^+]^{1+}$	average integral of IF, LF
L	I	938.72	470.36	15.35	572.42	589.46	716.56	$[M+H^+]^{1+}$	
L	K	953.73	477.865	10.51	572.42	604.47	731.57	$[M+2H^+]^{2+}$	
L	L	938.72	470.36	15.5	572.42	589.46	716.56	$[M+H^+]^{1+}$	
L	M	956.68	479.34	14.13	572.42	607.42	734.52	$[M+H^+]^{1+}$	average integral of IM, LM
L	N	939.68	470.84	11.95	572.42	590.42	717.52	$[M+H^+]^{1+}$	
L	Q	953.69	477.845	12.03	572.42	604.43	731.53	$[M+H^+]^{1+}$	
L	R	981.74	491.87	10.59	572.42	632.48	759.58	$[M+2H^+]^{2+}$	
L	S	912.67	457.335	12.29	572.42	563.41	690.51	$[M+H^+]^{1+}$	
L	T	926.68	464.34	12.77	572.42	577.42	704.52	$[M+H^+]^{1+}$	average integral of IT, LT
L	V	924.7	463.35	14.52	572.42	575.44	702.54	$[M+H^+]^{1+}$	
L	W	1011.71	506.855	14.87	572.42	662.45	789.55	$[M+H^+]^{1+}$	average integral of IW, LW
L	Y	988.7	495.35	13.45	572.42	639.44	766.54	$[M+H^+]^{1+}$	average integral of IY, LY
V	A	882.66	442.33	12.03	558.4	547.42	660.5	$[M+H^+]^{1+}$	
V	D	926.65	464.325	11.54	558.4	591.41	704.49	$[M+H^+]^{1+}$	
V	E	940.66	471.33	11.69	558.4	605.42	718.5	$[M+H^+]^{1+}$	
V	F	958.68	480.34	14.57	558.4	623.44	736.52	$[M+H^+]^{1+}$	
V	I	924.7	463.35	14.65	558.4	589.46	702.54	$[M+H^+]^{1+}$	average integral of VI, VL
V	K	939.71	470.855	9.81	558.4	604.47	717.55	$[M+2H^+]^{2+}$	
V	L	924.7	463.35	14.65	558.4	589.46	702.54	$[M+H^+]^{1+}$	average integral of VI, VL
V	M	942.66	472.33	13.33	558.4	607.42	720.5	$[M+H^+]^{1+}$	
V	N	925.66	463.83	11.15	558.4	590.42	703.5	$[M+H^+]^{1+}$	
V	Q	939.67	470.835	11.23	558.4	604.43	717.51	$[M+H^+]^{1+}$	
V	R	967.72	484.86	9.89	558.4	632.48	745.56	$[M+2H^+]^{2+}$	
V	S	898.65	450.325	10.52	558.4	563.41	676.49	$[M+H^+]^{1+}$	
V	T	912.66	457.33	11.97	558.4	577.42	690.5	$[M+H^+]^{1+}$	
V	V	910.68	456.34	13.82	558.4	575.44	688.52	$[M+H^+]^{1+}$	
V	W	997.69	499.845	14.19	558.4	662.45	775.53	$[M+H^+]^{1+}$	
V	Y	974.68	488.34	12.75	558.4	639.44	752.52	$[M+H^+]^{1+}$	

Table S9. Characterization data of H12 charged sublibrary.

β3-amino		Detected peaks			MS2 fragment ions			Pulldown assay	
3rd	6th	$[M+H^+]^{1+}$	$[M+2H^+]^{2+}$	Rt (min)	b5	y5	y6	Detected ion	Remark
D	A	898.63	450.315	10.89	574.37	547.42	676.47	$[M+H^+]^{1+}$	
D	D	942.62	472.31	10.66	574.37	591.41	720.46	$[M+H^+]^{1+}$	average integral of DD, ET
D	E	956.63	479.315	10.83	574.37	605.42	734.47	$[M+H^+]^{1+}$	average integral of DE, ED
D	F	974.65	488.325	12.99	574.37	623.44	752.49	$[M+H^+]^{1+}$	
D	I	940.67	471.335	12.87	574.37	589.46	718.51	$[M+H^+]^{1+}$	average integral of DI, DL
D	K	955.68	478.84	8.85	574.37	604.47	733.52	$[M+2H^+]^{2+}$	
D	L	940.67	471.335	12.87	574.37	589.46	718.51	$[M+H^+]^{1+}$	average integral of DI, DL
D	M	958.63	480.315	11.99	574.37	607.42	736.47	$[M+H^+]^{1+}$	
D	N	941.63	471.815	10.41	574.37	590.42	719.47	$[M+H^+]^{1+}$	
D	Q	955.64	478.82	10.12	574.37	604.43	733.48	$[M+H^+]^{1+}$	
D	R	983.69	492.845	9.04	574.37	632.48	761.53	$[M+2H^+]^{2+}$	
D	S	914.62	458.31	10.66	574.37	563.41	692.46	$[M+H^+]^{1+}$	
D	T	928.63	465.315	10.35	574.37	577.42	706.47	$[M+H^+]^{1+}$	
D	V	926.65	464.325	12.12	574.37	575.44	704.49	$[M+H^+]^{1+}$	
D	W	1013.66	507.83	12.96	574.37	662.45	791.5	$[M+H^+]^{1+}$	
D	Y	990.65	496.325	11.61	574.37	639.44	768.49	$[M+H^+]^{1+}$	
E	A	912.64	457.32	10.66	588.38	547.42	690.48	$[M+H^+]^{1+}$	
E	D	956.63	479.315	10.83	588.38	591.41	734.47	$[M+H^+]^{1+}$	average integral of DE, ED
E	E	970.64	486.32	10.55	588.38	605.42	748.48	$[M+H^+]^{1+}$	
E	F	988.66	495.33	12.86	588.38	623.44	766.5	$[M+H^+]^{1+}$	
E	I	954.68	478.34	12.65	588.38	589.46	732.52	$[M+H^+]^{1+}$	average integral of EI, EL
E	K	969.69	485.845	9	588.38	604.47	747.53	$[M+2H^+]^{2+}$	
E	L	954.68	478.34	12.7	588.38	589.46	732.52	$[M+H^+]^{1+}$	average integral of EI, EL
E	M	972.64	487.32	11.77	588.38	607.42	750.48	$[M+H^+]^{1+}$	
E	N	955.64	478.82	10.12	588.38	590.42	733.48	$[M+H^+]^{1+}$	
E	Q	969.65	485.825	10.21	588.38	604.43	747.49	$[M+H^+]^{1+}$	
E	R	997.7	499.85	9.1	588.38	632.48	775.54	$[M+2H^+]^{2+}$	average integral of ER, RE
E	S	928.63	465.315	10.35	588.38	563.41	706.47	$[M+H^+]^{1+}$	
E	T	942.64	472.32	10.66	588.38	577.42	720.48	$[M+H^+]^{1+}$	average integral of DD, ET
E	V	940.66	471.33	11.96	588.38	575.44	718.5	$[M+H^+]^{1+}$	
E	W	1027.67	514.835	12.71	588.38	662.45	805.51	$[M+H^+]^{1+}$	
E	Y	1004.66	503.33	11.43	588.38	639.44	782.5	$[M+H^+]^{1+}$	
K	A	911.69	456.845	8.95	587.43	547.42	689.53	$[M+2H^+]^{2+}$	
K	D	955.68	478.84	8.85	587.43	591.41	733.52	$[M+2H^+]^{2+}$	
K	E	969.69	485.845	9	587.43	605.42	747.53	$[M+2H^+]^{2+}$	
K	F	987.71	494.855	10.88	587.43	623.44	765.55	$[M+2H^+]^{2+}$	
K	I	953.73	477.865	10.6	587.43	589.46	731.57	$[M+2H^+]^{2+}$	
K	K	968.74	485.37	7.52	587.43	604.47	746.58	$[M+2H^+]^{2+}$	
K	L	953.73	477.865	10.67	587.43	589.46	731.57	$[M+2H^+]^{2+}$	
K	M	971.69	486.845	9.89	587.43	607.42	749.53	$[M+2H^+]^{2+}$	
K	N	954.69	478.345	8.67	587.43	590.42	732.53	$[M+2H^+]^{2+}$	
K	Q	968.7	485.35	8.68	587.43	604.43	746.54	$[M+2H^+]^{2+}$	
K	R	996.75	499.375	7.57	587.43	632.48	774.59	$[M+2H^+]^{2+}$	average integral of KR, RK
K	S	927.68	464.84	8.79	587.43	563.41	705.52	$[M+2H^+]^{2+}$	
K	T	941.69	471.845	9.07	587.43	577.42	719.53	$[M+2H^+]^{2+}$	
K	V	939.71	470.855	9.03	587.43	575.44	717.55	$[M+2H^+]^{2+}$	
K	W	1026.72	514.36	10.77	587.43	662.45	804.56	$[M+2H^+]^{2+}$	
K	Y	1003.71	502.855	9.74	587.43	639.44	781.55	$[M+2H^+]^{2+}$	
R	A	939.7	470.85	9.03	615.44	547.42	717.54	$[M+2H^+]^{2+}$	
R	D	983.69	492.845	9.04	615.44	591.41	761.53	$[M+2H^+]^{2+}$	
R	E	997.7	499.85	9.1	615.44	605.42	775.54	$[M+2H^+]^{2+}$	average integral of ER, RE
R	F	1015.72	508.86	11	615.44	623.44	793.56	$[M+2H^+]^{2+}$	
R	I	981.74	491.87	10.71	615.44	589.46	759.58	$[M+2H^+]^{2+}$	average integral of RI, RL
R	K	996.75	499.375	7.57	615.44	604.47	774.59	$[M+2H^+]^{2+}$	average integral of KR, RK
R	L	981.74	491.87	10.76	615.44	589.46	759.58	$[M+2H^+]^{2+}$	average integral of RI, RL
R	M	999.7	500.85	9.98	615.44	607.42	777.54	$[M+2H^+]^{2+}$	
R	N	982.7	492.35	8.74	615.44	590.42	760.54	$[M+2H^+]^{2+}$	
R	Q	996.71	499.355	8.75	615.44	604.43	774.55	$[M+2H^+]^{2+}$	
R	R	1024.76	513.38	7.64	615.44	632.48	802.6	$[M+2H^+]^{2+}$	
R	S	955.69	478.845	9.52	615.44	563.41	733.53	$[M+2H^+]^{2+}$	
R	T	969.7	485.85	9	615.44	577.42	747.54	$[M+2H^+]^{2+}$	
R	V	967.72	484.86	10.16	615.44	575.44	745.56	$[M+2H^+]^{2+}$	
R	W	1054.73	528.365	10.86	615.44	662.45	832.57	$[M+2H^+]^{2+}$	
R	Y	1031.72	516.86	9.8	615.44	639.44	809.56	$[M+2H^+]^{2+}$	

Table S10. Characterization data of H12 polar sublibrary.

β3-amino		Detected peaks			MS2 fragment ions			Pulldown assay	
3rd	6th	$[M+H^+]^{1+}$	$[M+2H^+]^{2+}$	Rt (min)	b5	y5	y6	Detected ion	Remark
N	A	897.64	449.82	10.44	573.38	547.42	675.48	$[M+H^+]^{1+}$	
N	D	941.63	471.815	10.28	573.38	591.41	719.47	$[M+H^+]^{1+}$	
N	E	955.64	478.82	10.14	573.38	605.42	733.48	$[M+H^+]^{1+}$	
N	F	973.66	487.83	12.48	573.38	623.44	751.5	$[M+H^+]^{1+}$	
N	I	939.68	470.84	12.3	573.38	589.46	717.52	$[M+H^+]^{1+}$	average integral of NI, NL
N	K	954.69	478.345	8.91	573.38	604.47	732.53	$[M+2H^+]^{2+}$	
N	L	939.68	470.84	12.3	573.38	589.46	717.52	$[M+H^+]^{1+}$	average integral of NI, NL
N	M	957.64	479.82	11.48	573.38	607.42	735.48	$[M+H^+]^{1+}$	
N	N	940.64	471.32	10.02	573.38	590.42	718.48	$[M+H^+]^{1+}$	
N	Q	954.65	478.325	9.79	573.38	604.43	732.49	$[M+H^+]^{1+}$	
N	R	982.7	492.35	8.94	573.38	632.48	760.54	$[M+2H^+]^{2+}$	
N	S	913.63	457.815	10.26	573.38	563.41	691.47	$[M+H^+]^{1+}$	
N	T	927.64	464.82	10.5	573.38	577.42	705.48	$[M+H^+]^{1+}$	
N	V	925.66	463.83	11.57	573.38	575.44	703.5	$[M+H^+]^{1+}$	
N	W	1012.67	507.335	12.45	573.38	662.45	790.51	$[M+H^+]^{1+}$	
N	Y	989.66	495.83	11.14	573.38	639.44	767.5	$[M+H^+]^{1+}$	
Q	A	911.65	456.825	10.32	587.39	547.42	689.49	$[M+H^+]^{1+}$	
Q	D	955.64	478.82	10.42	587.39	591.41	733.48	$[M+H^+]^{1+}$	
Q	E	969.65	485.825	10.27	587.39	605.42	747.49	$[M+H^+]^{1+}$	
Q	F	987.67	494.835	12.42	587.39	623.44	765.51	$[M+H^+]^{1+}$	
Q	I	953.69	477.845	12.23	587.39	589.46	731.53	$[M+H^+]^{1+}$	average integral of QI, QL
Q	K	968.7	485.35	8.79	587.39	604.47	746.54	$[M+2H^+]^{2+}$	
Q	L	953.69	477.845	12.23	587.39	589.46	731.53	$[M+H^+]^{1+}$	average integral of QI, QL
Q	M	971.65	486.825	11.38	587.39	607.42	749.49	$[M+H^+]^{1+}$	
Q	N	954.65	478.325	10.04	587.39	590.42	732.49	$[M+H^+]^{1+}$	
Q	Q	968.66	485.33	9.94	587.39	604.43	746.5	$[M+H^+]^{1+}$	
Q	R	996.71	499.355	8.84	587.39	632.48	774.55	$[M+2H^+]^{2+}$	
Q	S	927.64	464.82	10.07	587.39	563.41	705.48	$[M+H^+]^{1+}$	average integral of QS, SQ
Q	T	941.65	471.825	10.36	587.39	577.42	719.49	$[M+H^+]^{1+}$	average integral of QT, TQ
Q	V	939.67	470.835	11.52	587.39	575.44	717.51	$[M+H^+]^{1+}$	
Q	W	1026.68	514.34	12.34	587.39	662.45	804.52	$[M+H^+]^{1+}$	
Q	Y	1003.67	502.835	11.07	587.39	639.44	781.51	$[M+H^+]^{1+}$	
S	A	870.63	436.315	10.46	546.37	547.42	648.47	$[M+H^+]^{1+}$	
S	D	914.62	458.31	10.87	546.37	591.41	692.46	$[M+H^+]^{1+}$	
S	E	928.63	465.315	10.31	546.37	605.42	706.47	$[M+H^+]^{1+}$	
S	F	946.65	474.325	12.58	546.37	623.44	724.49	$[M+H^+]^{1+}$	
S	I	912.67	457.335	12.38	546.37	589.46	690.51	$[M+H^+]^{1+}$	average integral of SI, SL
S	K	927.68	464.84	8.89	546.37	604.47	705.52	$[M+2H^+]^{2+}$	
S	L	912.67	457.335	12.38	546.37	589.46	690.51	$[M+H^+]^{1+}$	average integral of SI, SL
S	M	930.63	466.315	11.53	546.37	607.42	708.47	$[M+H^+]^{1+}$	
S	N	913.63	457.815	10.02	546.37	590.42	691.47	$[M+H^+]^{1+}$	
S	Q	927.64	464.82	10.07	546.37	604.43	705.48	$[M+H^+]^{1+}$	average integral of QS, SQ
S	R	955.69	478.845	8.97	546.37	632.48	733.53	$[M+2H^+]^{2+}$	
S	S	886.62	444.31	10.22	546.37	563.41	664.46	$[M+H^+]^{1+}$	
S	T	900.63	451.315	10.56	546.37	577.42	678.47	$[M+H^+]^{1+}$	average integral of ST, TS
S	V	898.65	450.325	11.68	546.37	575.44	676.49	$[M+H^+]^{1+}$	
S	W	985.66	493.83	12.52	546.37	662.45	763.5	$[M+H^+]^{1+}$	
S	Y	962.65	482.325	11.17	546.37	639.44	740.49	$[M+H^+]^{1+}$	
T	A	884.64	443.32	10.84	560.38	547.42	662.48	$[M+H^+]^{1+}$	
T	D	928.63	465.315	10.51	560.38	591.41	706.47	$[M+H^+]^{1+}$	
T	E	942.64	472.32	10.74	560.38	605.42	720.48	$[M+H^+]^{1+}$	
T	F	960.66	481.33	13.04	560.38	623.44	738.5	$[M+H^+]^{1+}$	
T	I	926.68	464.34	12.86	560.38	589.46	704.52	$[M+H^+]^{1+}$	
T	K	941.69	471.845	9.16	560.38	604.47	719.53	$[M+2H^+]^{2+}$	
T	L	926.68	464.34	12.86	560.38	589.46	704.52	$[M+H^+]^{1+}$	
T	M	944.64	473.32	11.97	560.38	607.42	722.48	$[M+H^+]^{1+}$	
T	N	927.64	464.82	10.32	560.38	590.42	705.48	$[M+H^+]^{1+}$	
T	Q	941.65	471.825	10.36	560.38	604.43	719.49	$[M+H^+]^{1+}$	average integral of QT, TQ
T	R	969.7	485.85	9.23	560.38	632.48	747.54	$[M+2H^+]^{2+}$	
T	S	900.63	451.315	10.56	560.38	563.41	678.47	$[M+H^+]^{1+}$	average integral of ST, TS
T	T	914.64	458.32	10.24	560.38	577.42	692.48	$[M+H^+]^{1+}$	
T	V	912.66	457.33	12.15	560.38	575.44	690.5	$[M+H^+]^{1+}$	
T	W	999.67	500.835	12.92	560.38	662.45	777.51	$[M+H^+]^{1+}$	
T	Y	976.66	489.33	11.55	560.38	639.44	754.5	$[M+H^+]^{1+}$	

Peptide characterisation data

Table S11. Molecular mass and m/z data of separately synthetized foldamers.

	Calculated MW	$[M+H]^+$	$[M+2H]^{2+}$
**1a**	1461.06	1462.06	721.53
**1b**	918.54	919.54	460.27
**2a**	1388.07	1389.07	695.04
**3a**	1399.04	1400.04	700.52
**4a**	1349.06	1350.06	675.53
**5a**	1376.03	1377.03	689.02
**6a**	1392.08	1393.08	697.04
**7a**	1315.08	1316.08	658.54
**8a**	1301.06	1302.06	651.53
**9a**	1401.12	1402.12	701.56
**10a**	1303.04	1304.04	652.52
**10b**	760.52	761.52	381.26
**11a**	1321.00	1322.00	661.50
**11b**	778.48	779.48	390.24
**12b**	879.53	880.53	440.77

Dataset S1. HPLC-MS data

Compound **1a**

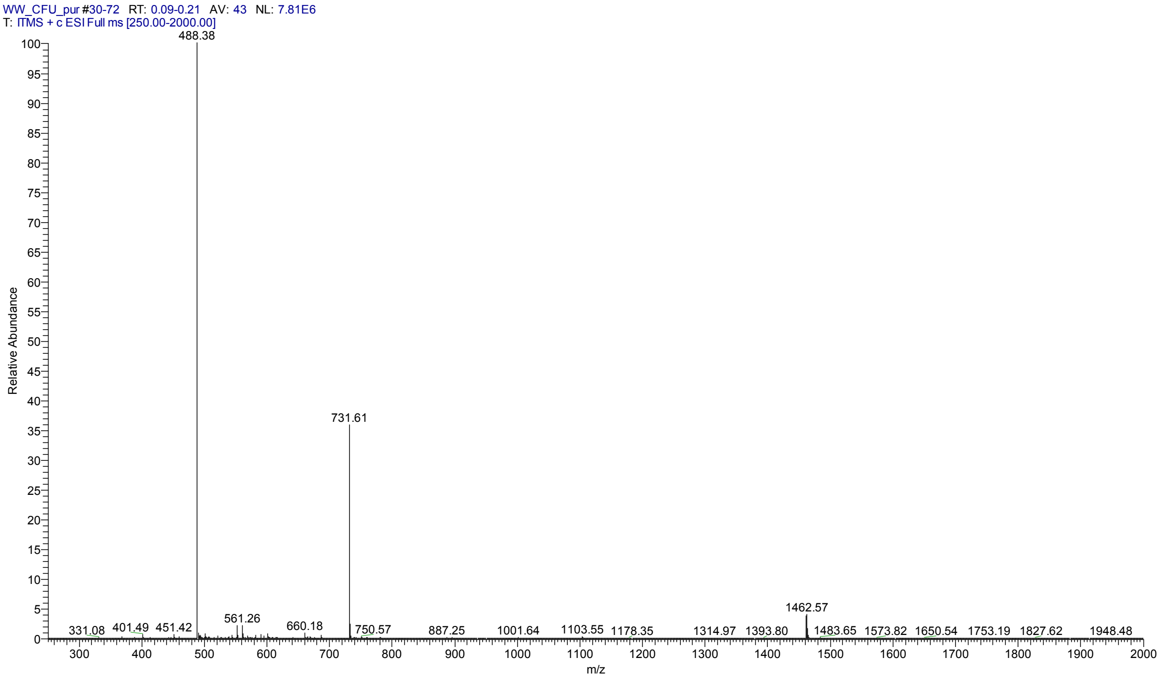

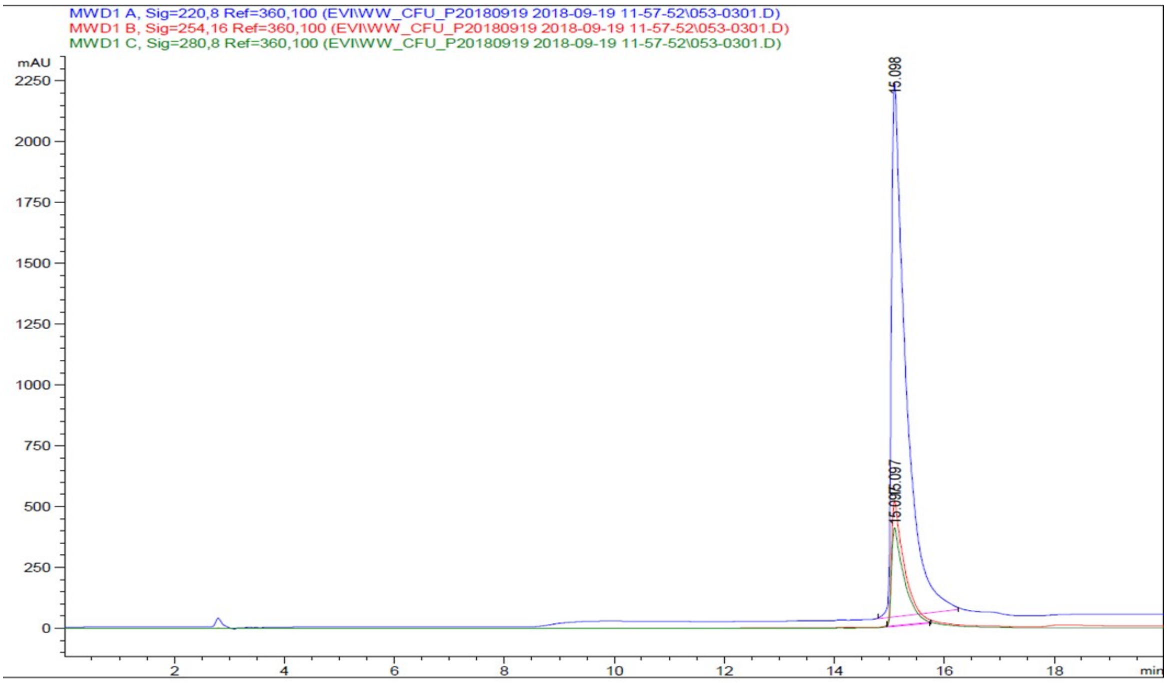

Column: Phenomenex Luna C18 (250 x 4.6 mm, particle size: 5 micron, pore size: 100Å); Gradient: 5-80% 20min 1.2 mL min^{-1}

Compound **1b**

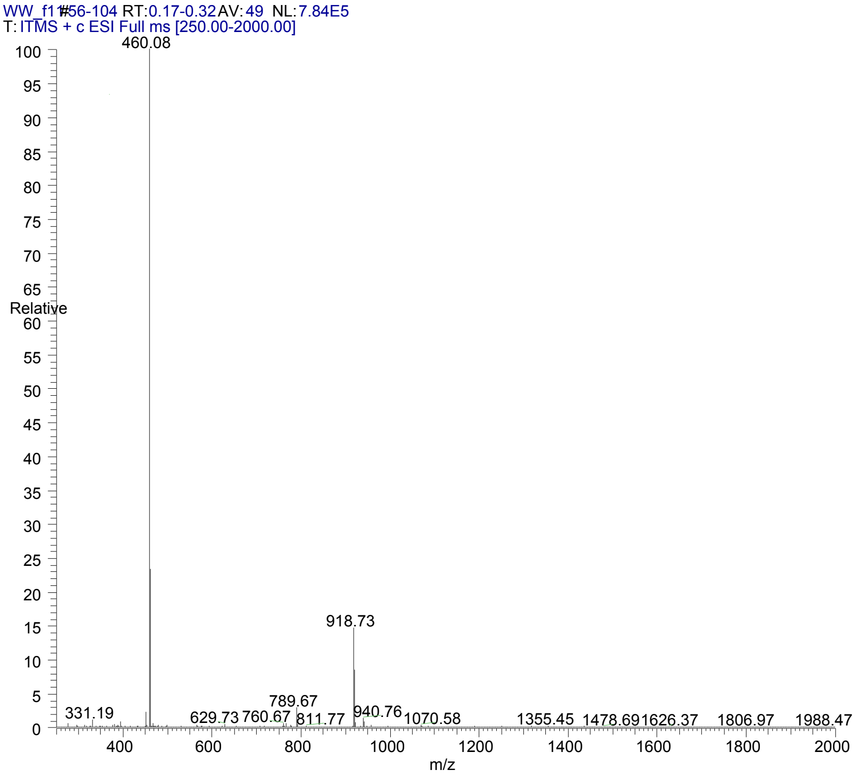

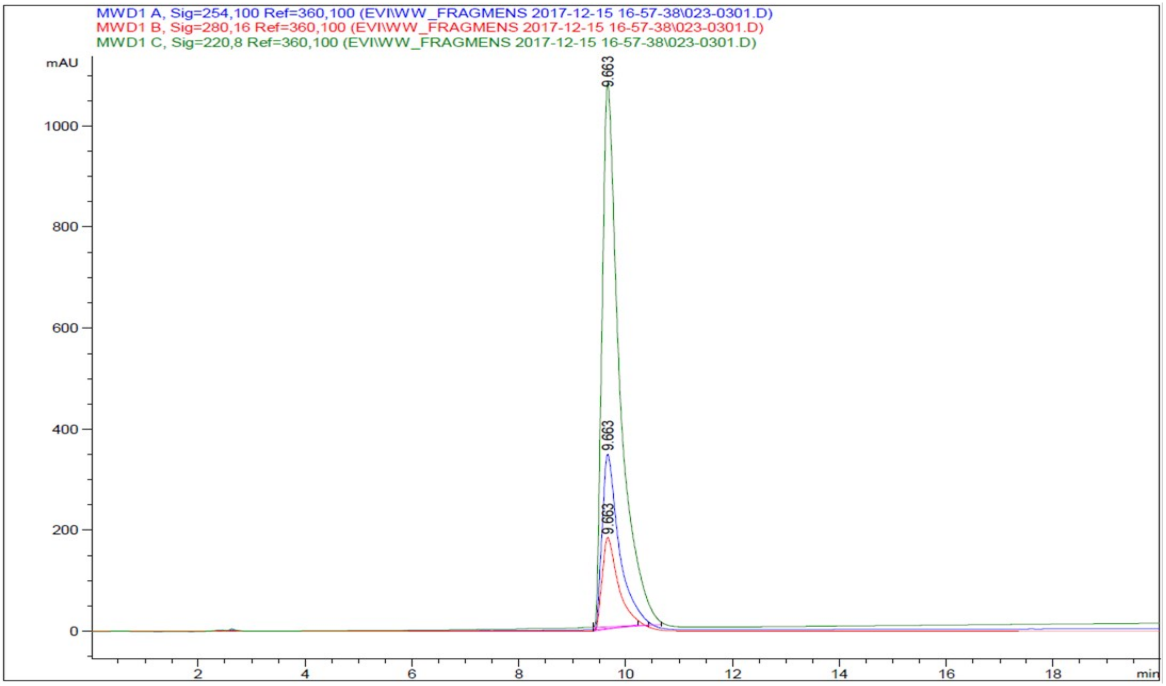

Column: Phenomenex Luna C18 (250 x 4.6 mm, particle size: 5 micron, pore size: 100Å); Gradient: 50-70% 20min 1.2 mL min^{-1}

Compound **2a**

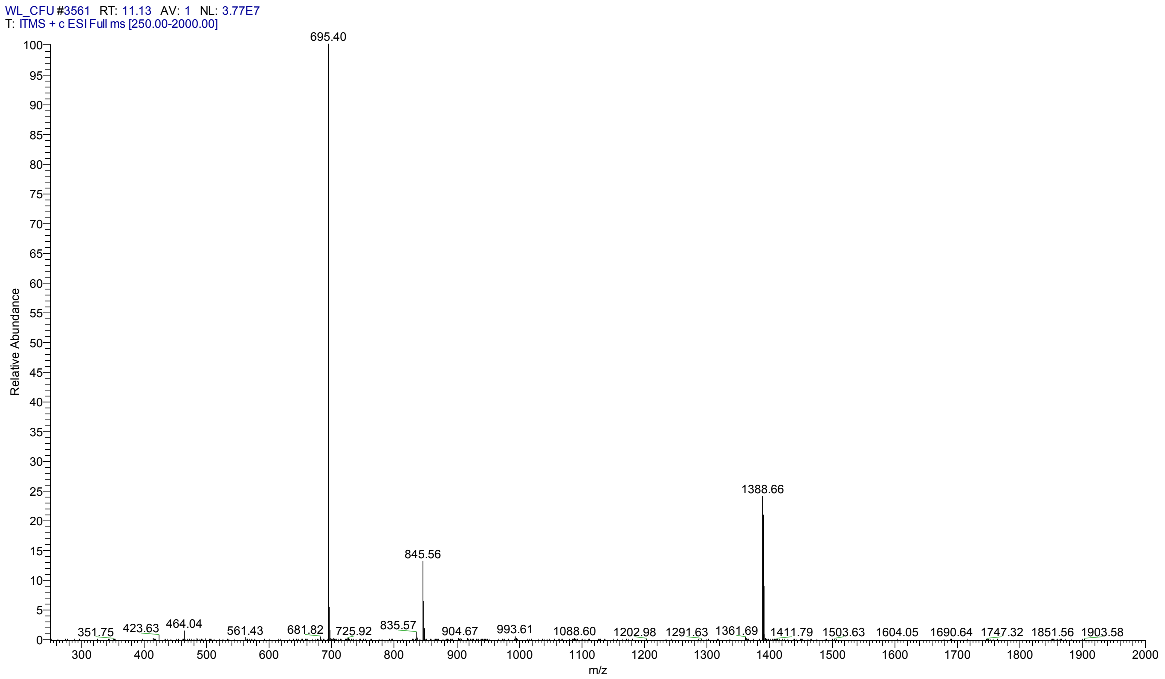

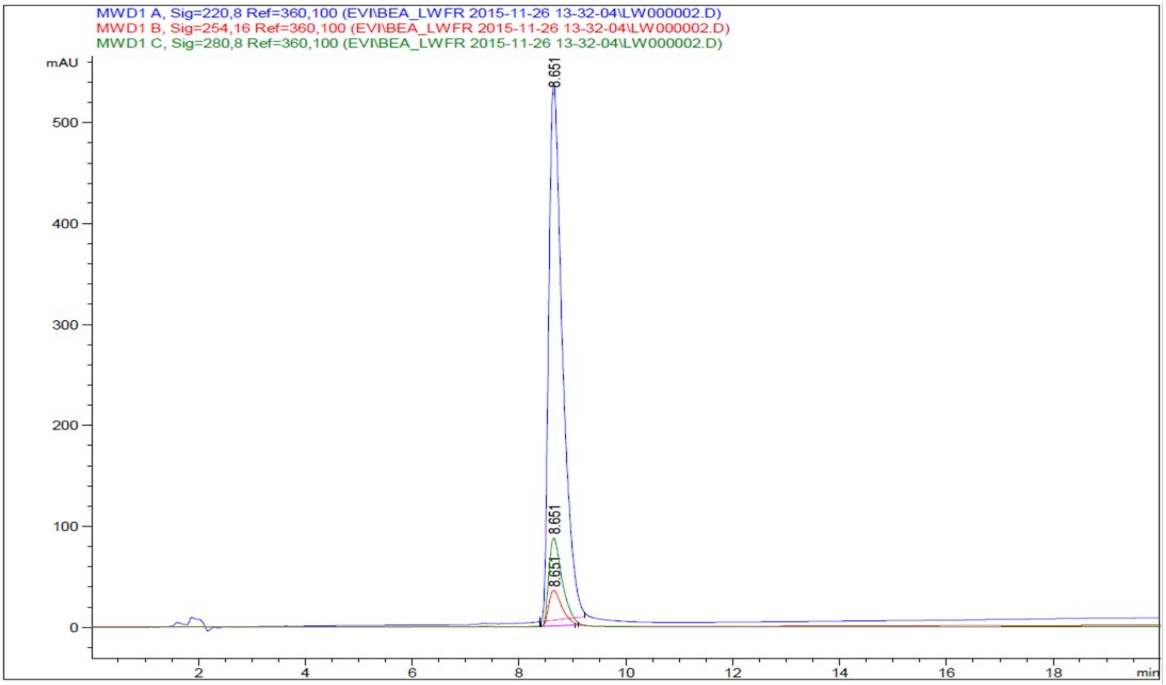

Column: Phenomenex Luna C18 (250 x 4.6 mm, particle size: 5 micron, pore size: 100Å); Gradient: 60-80% 20min 1.2 mL min^{-1}

Compound **3a**

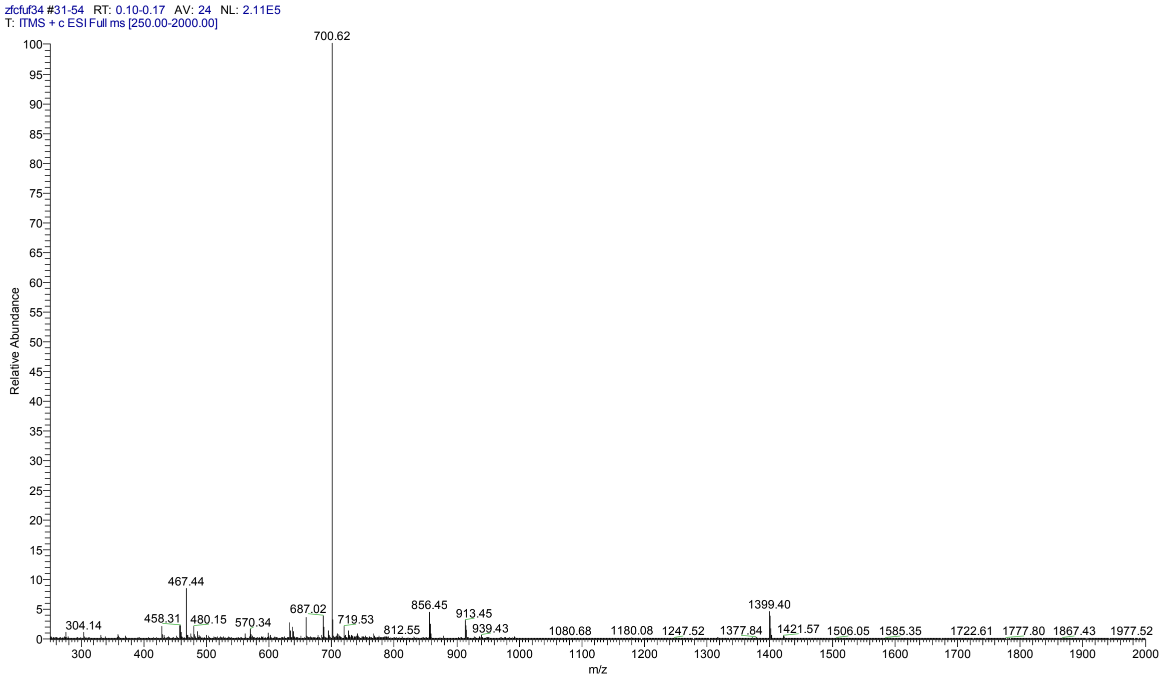

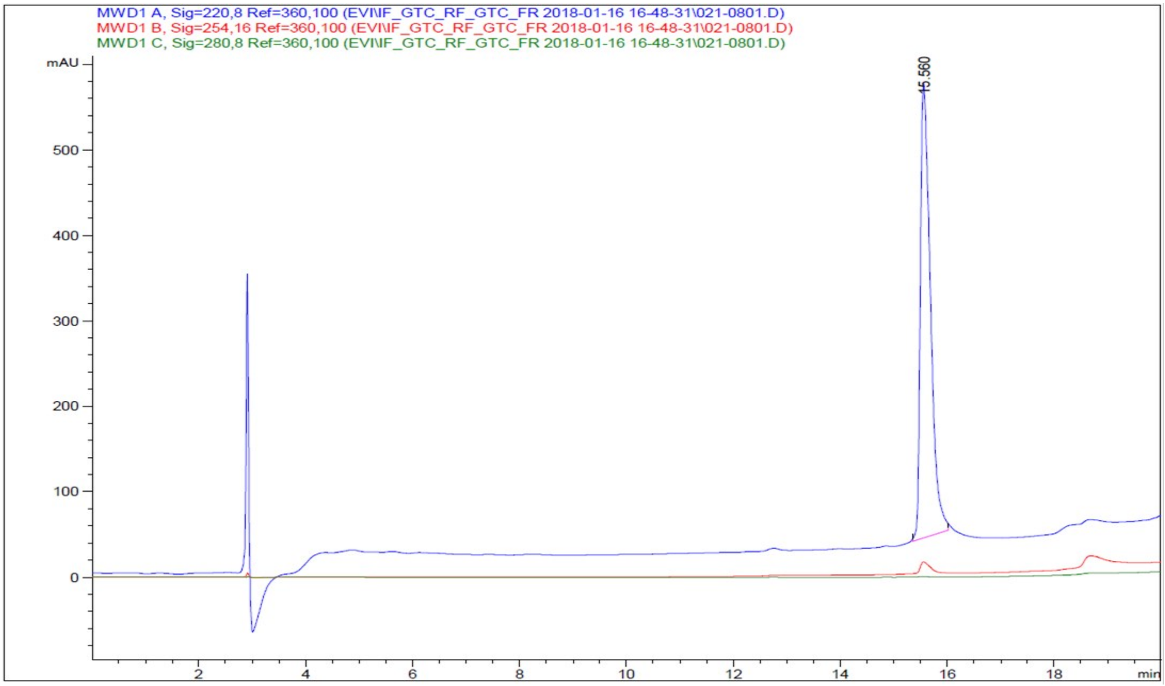

Column: Phenomenex Luna C18 (250 x 4.6 mm, particle size: 5 micron, pore size: 100Å); Gradient: 5-80% 20min 1.2 mL min^{-1}

Compound **4a**

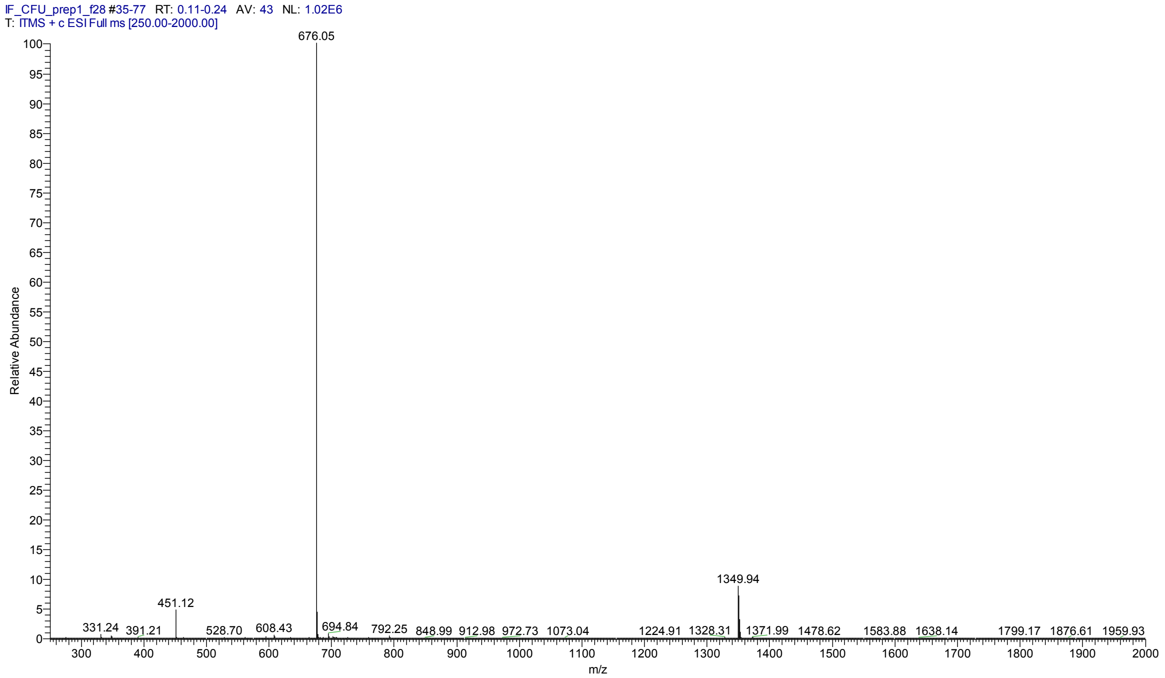

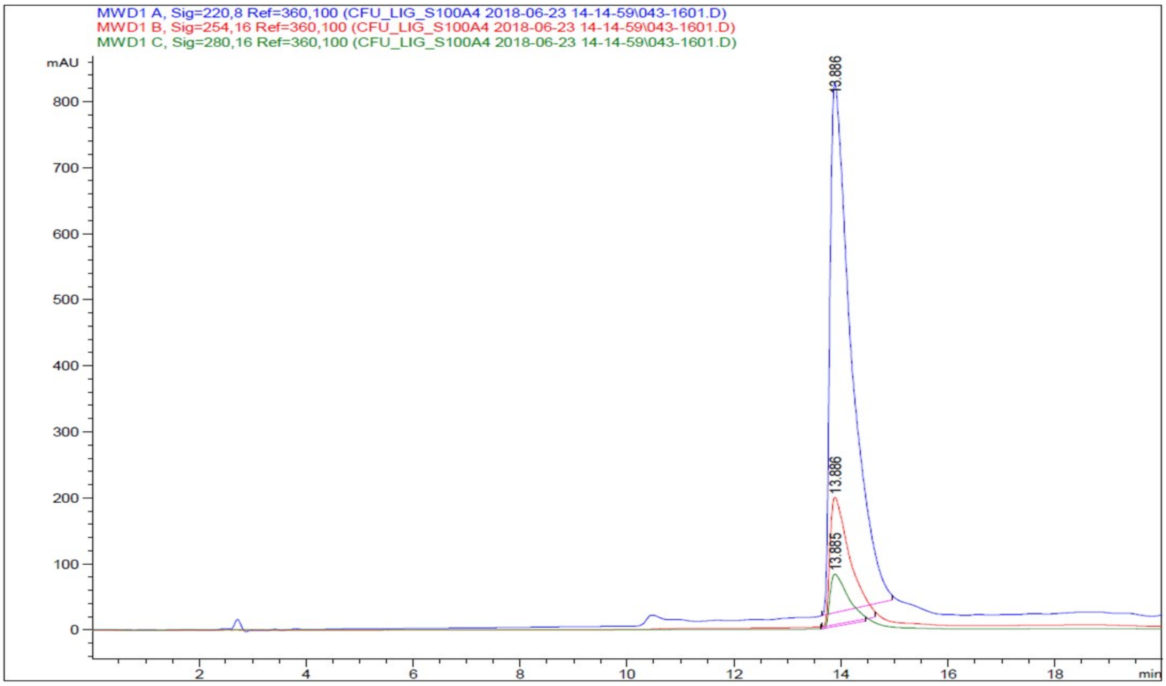

Column: Phenomenex Luna C18 (250 x 4.6 mm, particle size: 5 micron, pore size: 100Å); Gradient: 40-60% 20min 1.2 mL min^{-1}

Compound **5a**

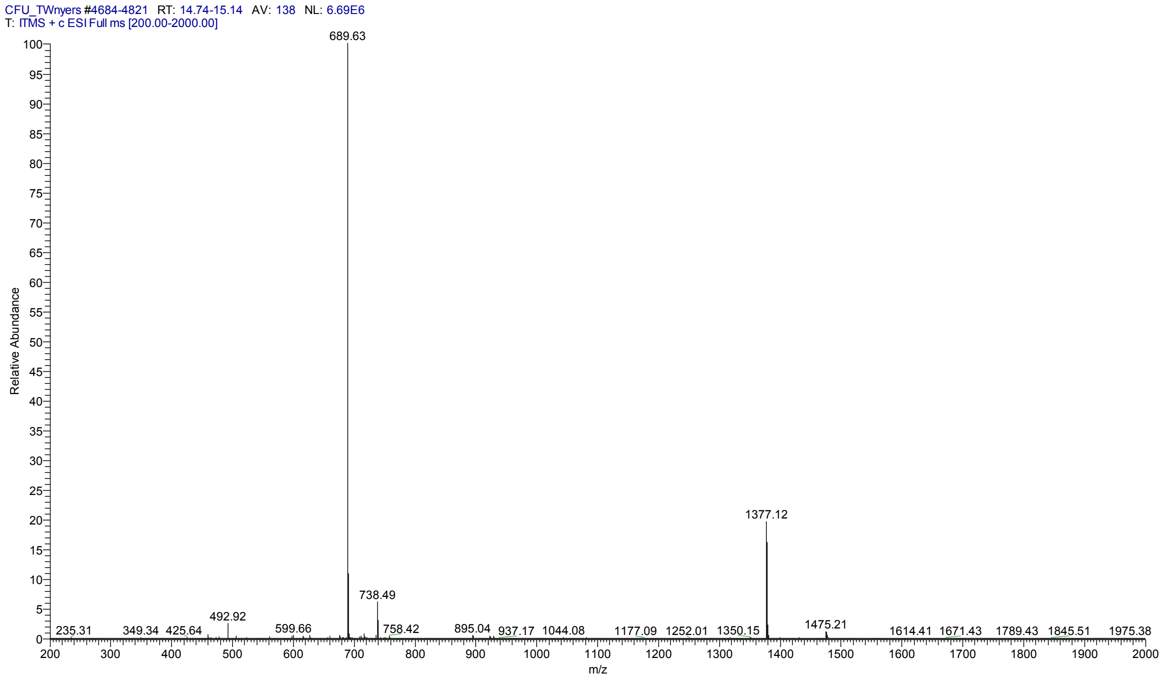

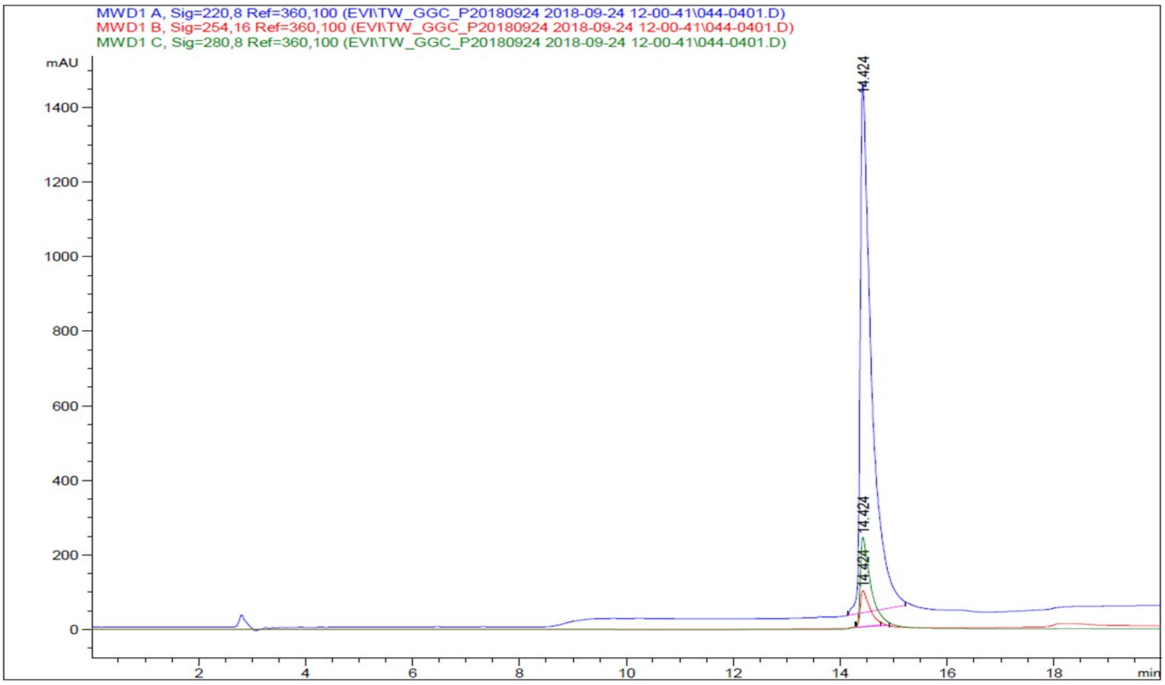

Column: Phenomenex Luna C18 (250 x 4.6 mm, particle size: 5 micron, pore size: 100Å); Gradient: 5-80% 20min 1.2 mL min^{-1}

Compound **6a**

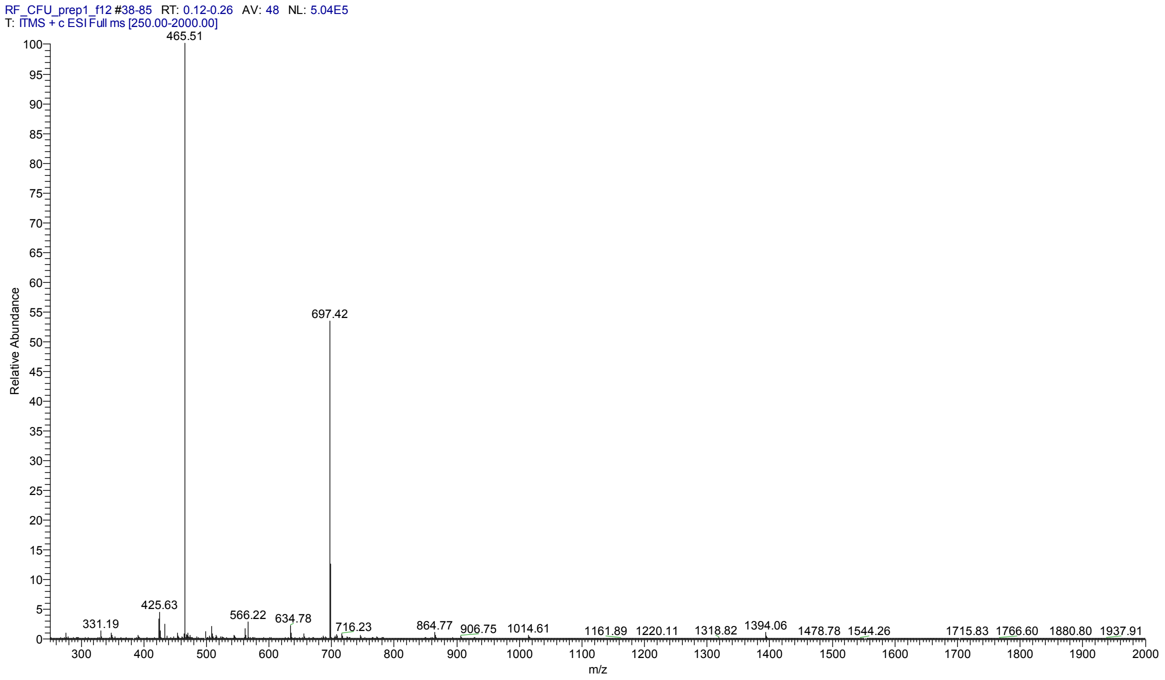

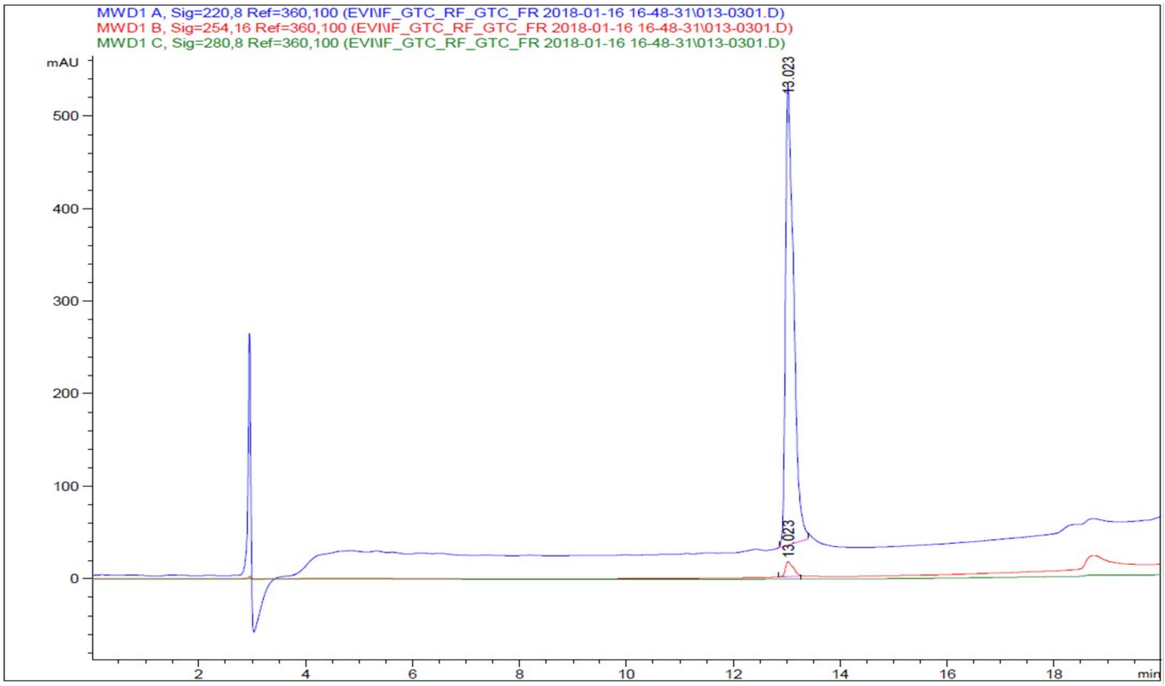

Column: Phenomenex Luna C18 (250 x 4.6 mm, particle size: 5 micron, pore size: 100Å); Gradient: 5-80% 20min 1.2 mL min^{-1}

Compound **7a**

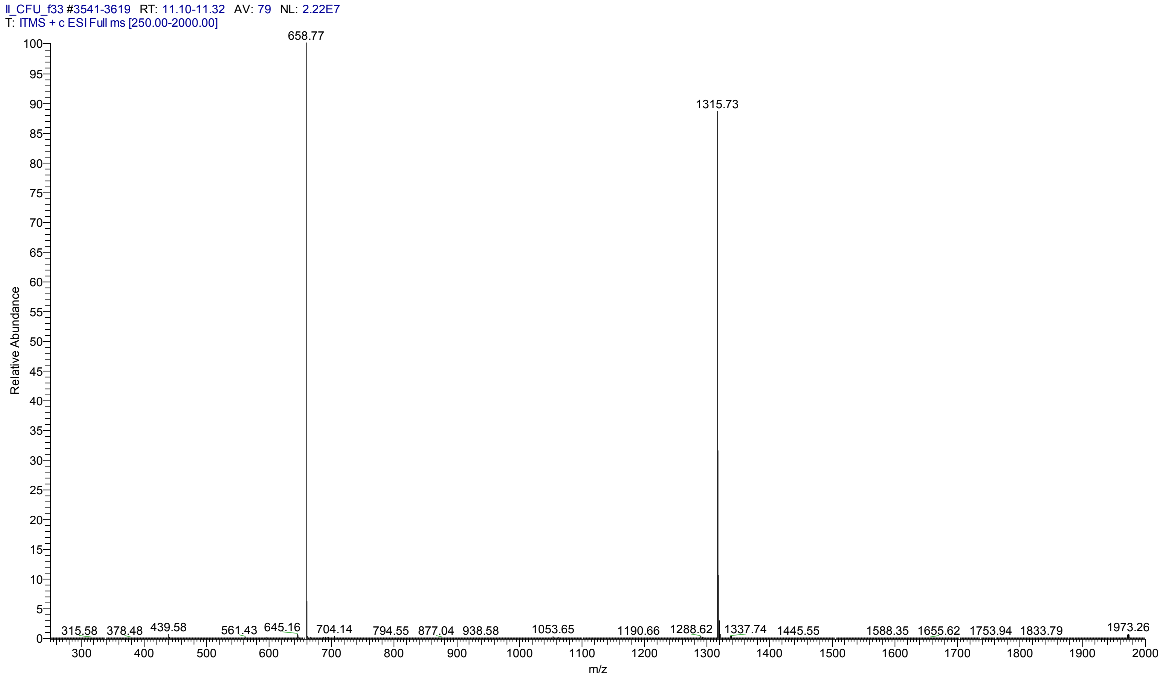

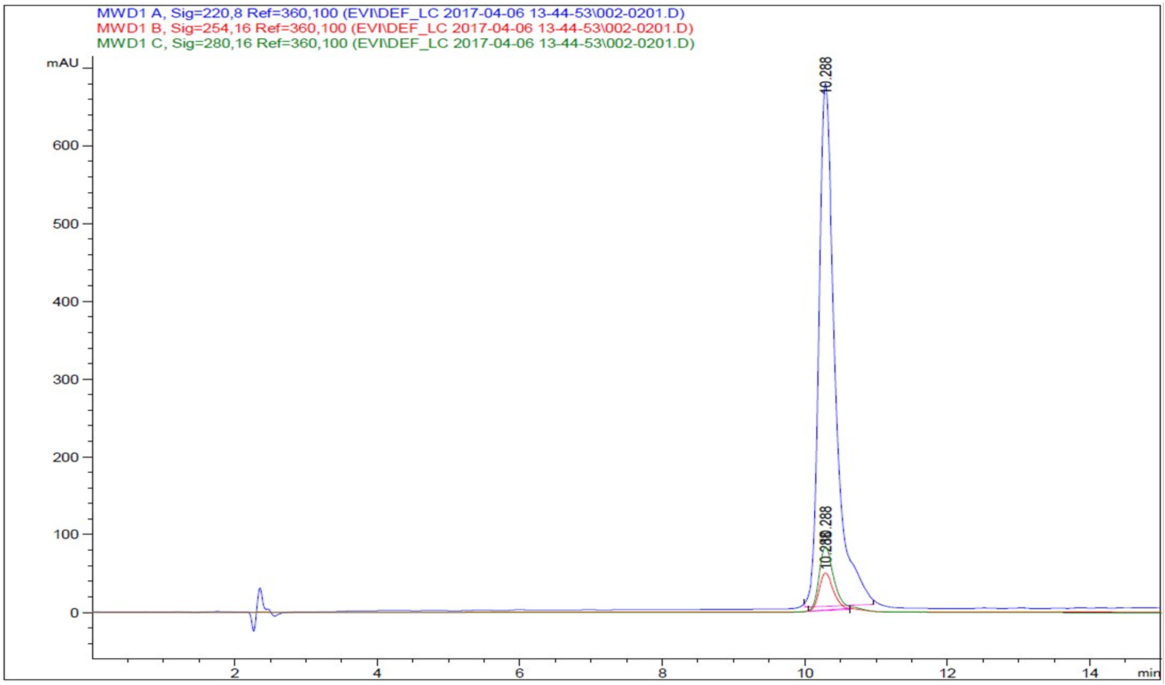

Column: Phenomenex Luna C18 (250 x 4.6 mm, particle size: 5 micron, pore size: 100Å); Gradient: 40-60% 20min 1.2 mL min^{-1}

Compound **8a**

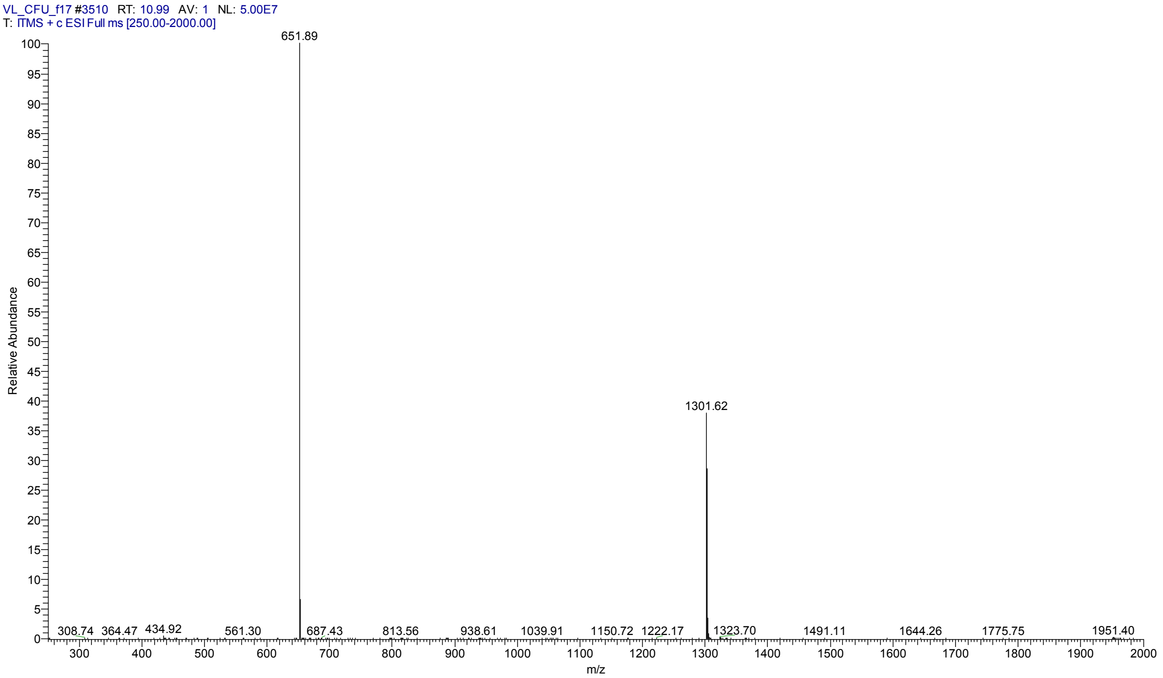

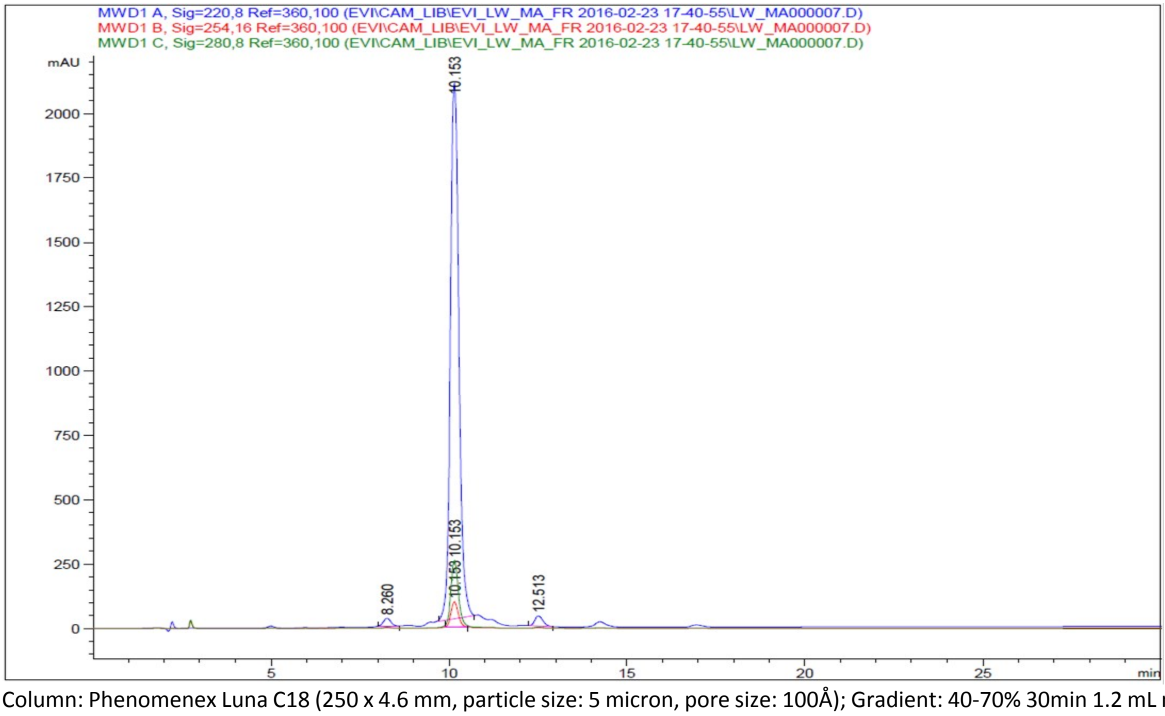

Column: Phenomenex Luna C18 (250 x 4.6 mm, particle size: 5 micron, pore size: 100Å); Gradient: 40-70% 30min 1.2 mL min^{-1}

Compound **9a**

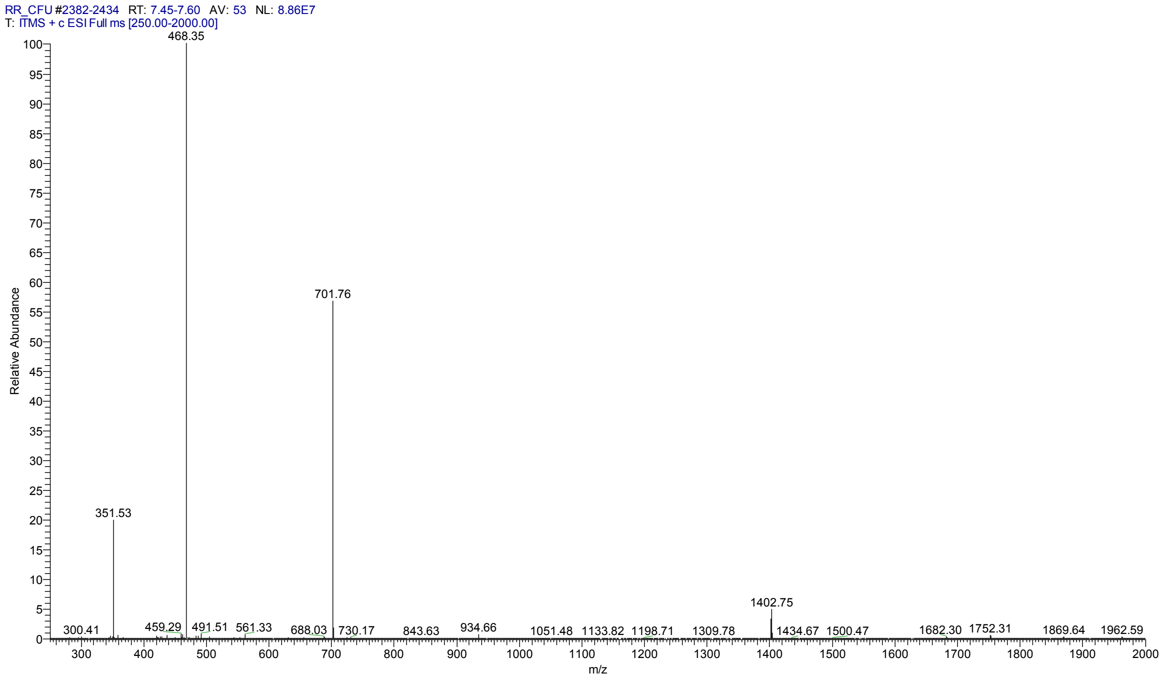

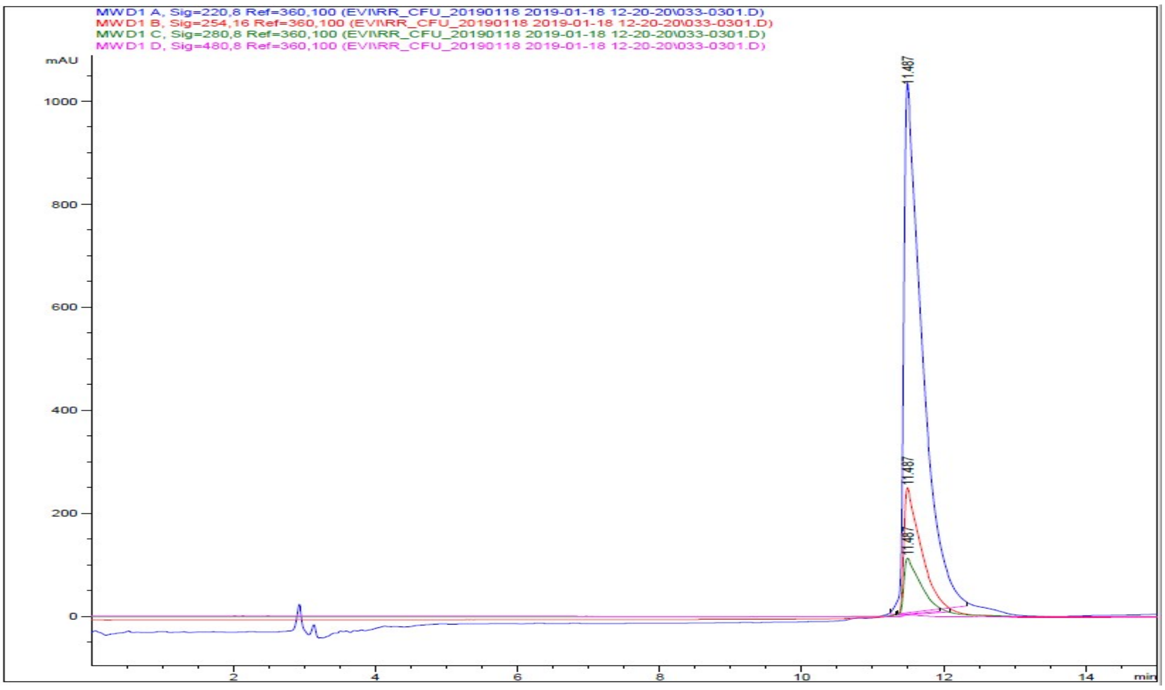

Column: Phenomenex Luna C18 (250 x 4.6 mm, particle size: 5 micron, pore size: 100Å); Gradient: 5-80% 20min 1.2 mL min^{-1}

Compound **10a**

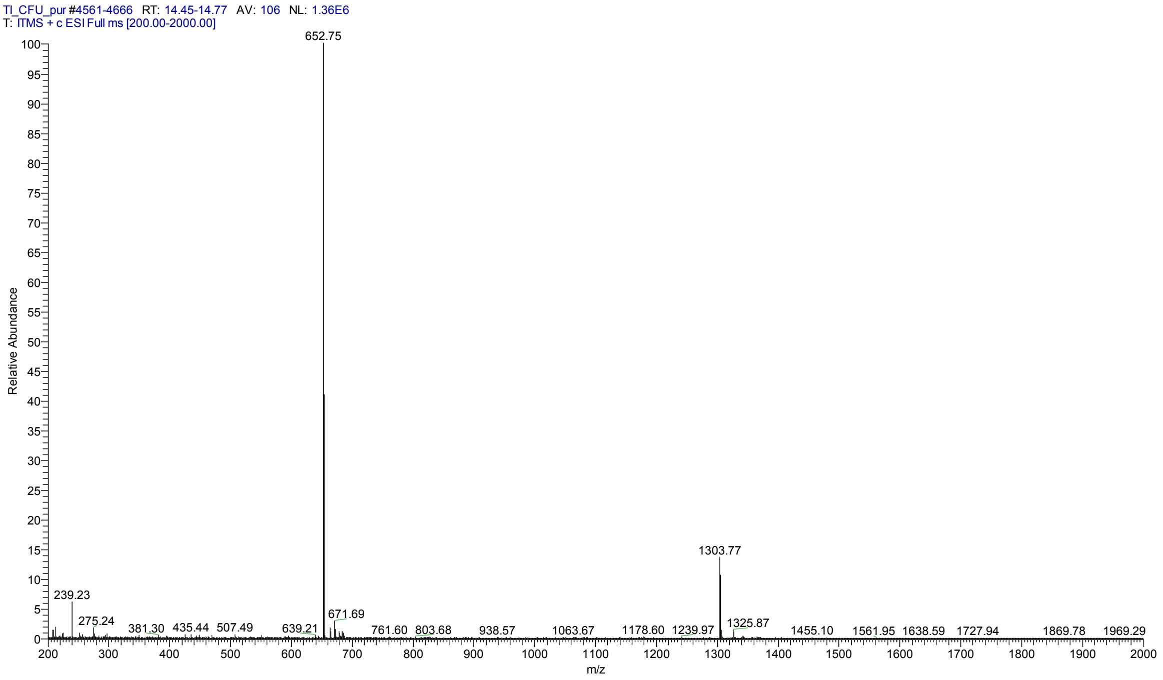

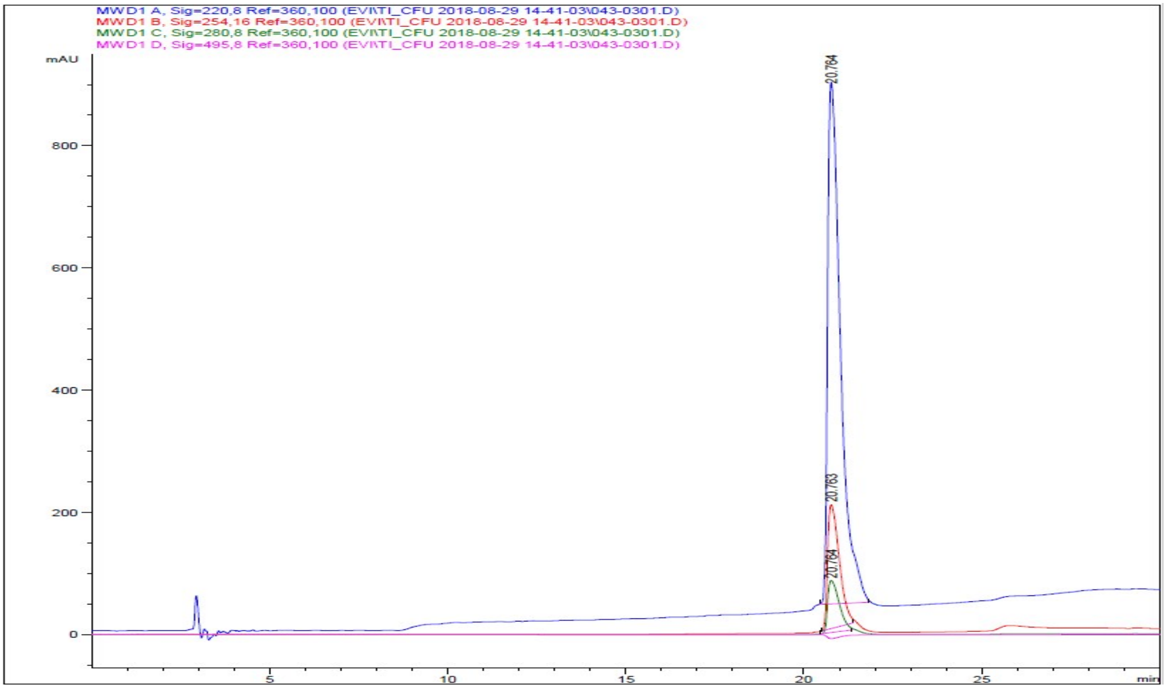

Column: Phenomenex Luna C18 (250 x 4.6 mm, particle size: 5 micron, pore size: 100Å); Gradient: 5-80% 30min 1.2 mL min^{-1}

Compound **10b**

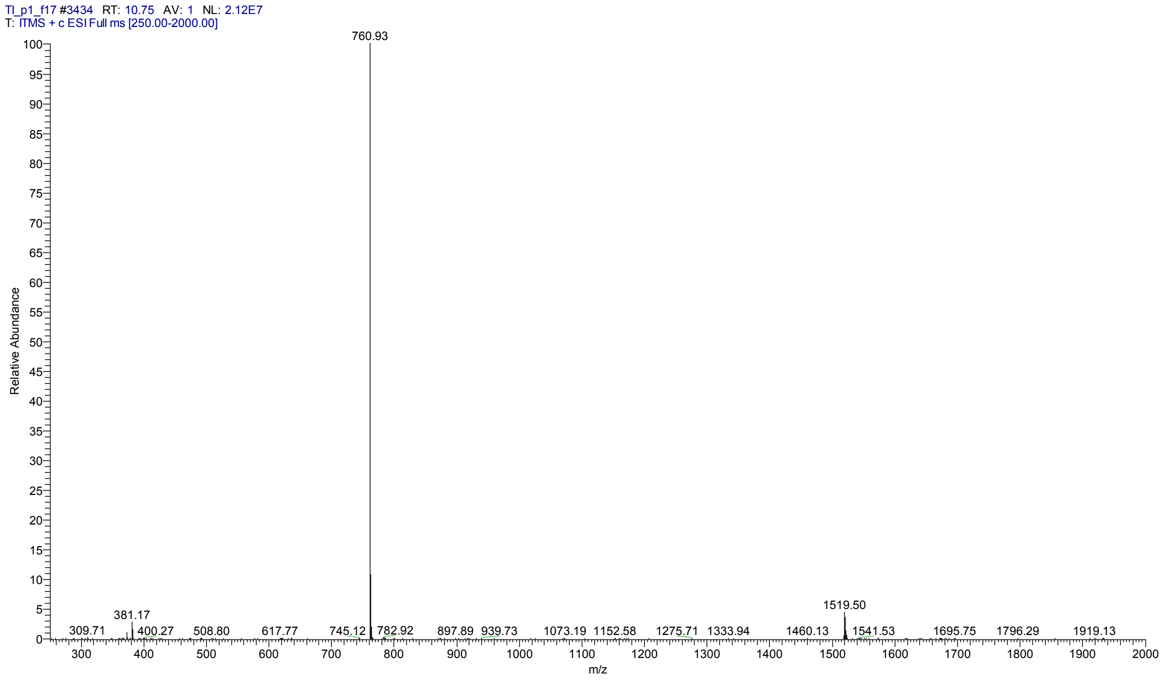

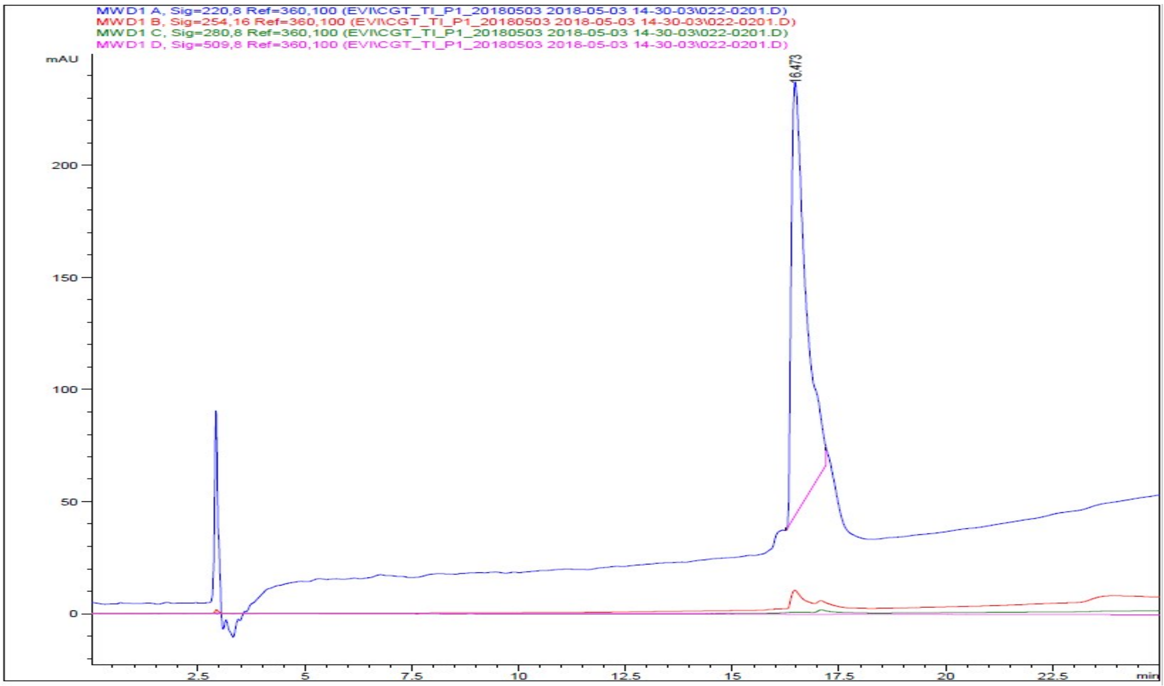

Column: Phenomenex Luna C18 (250 x 4.6 mm, particle size: 5 micron, pore size: 100Å); Gradient: 5-80% 25min 1.2 mL min^{-1}

Compound **11a**

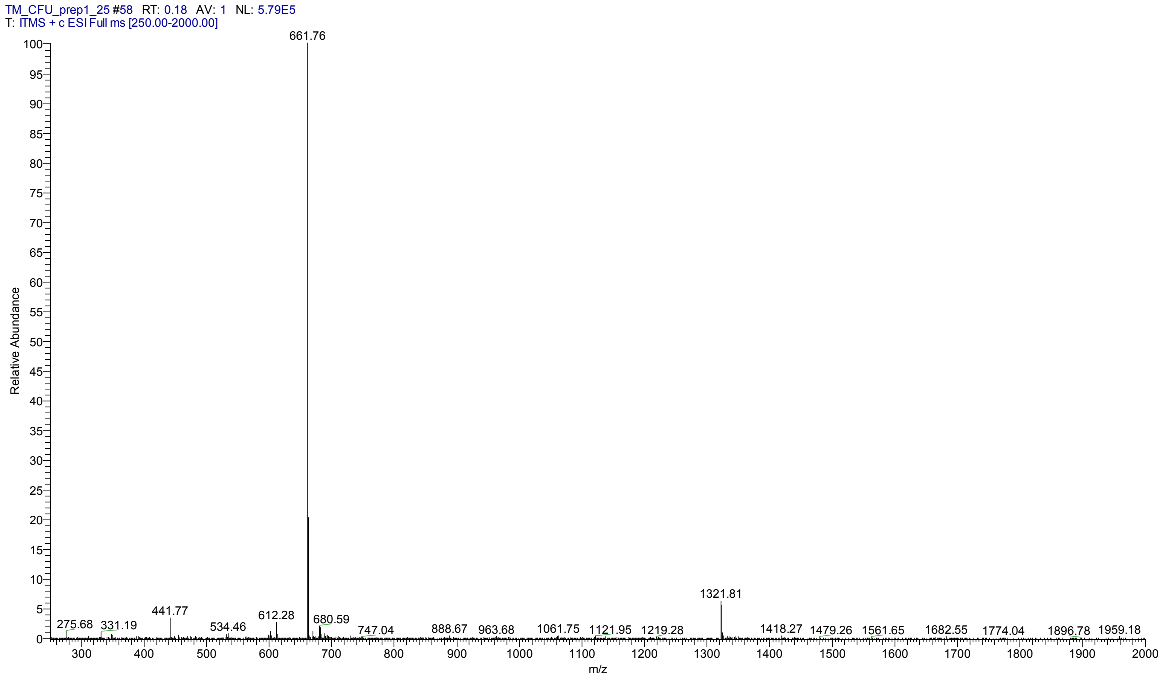

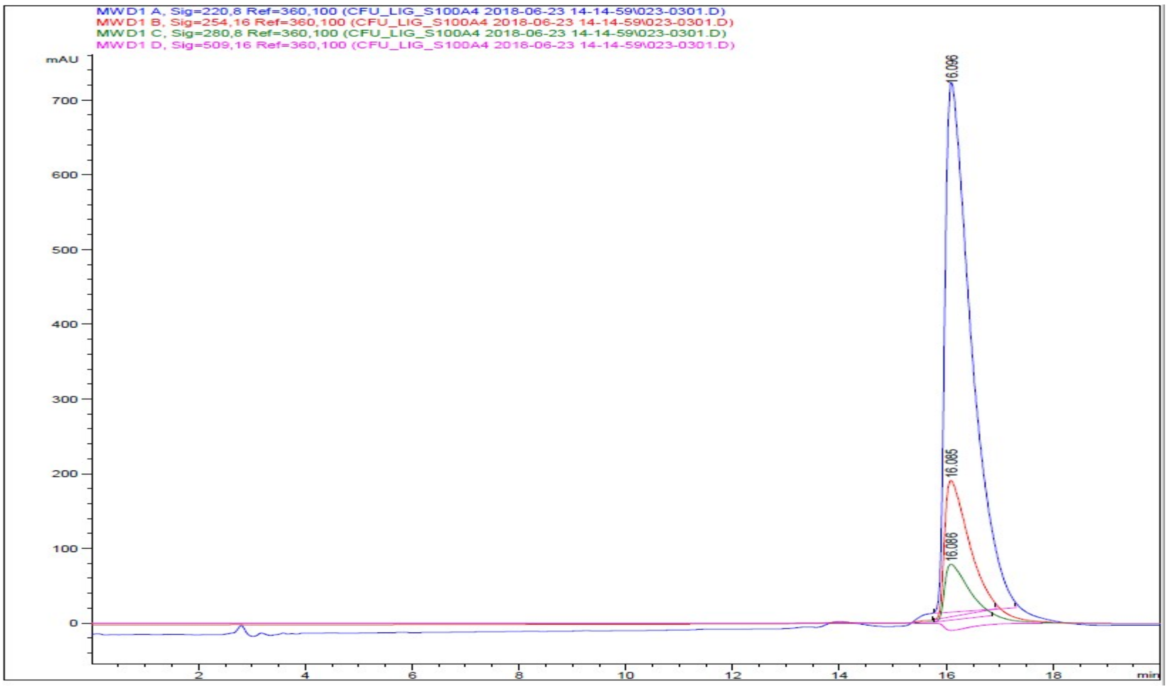

Column: Phenomenex Luna C18 (250 x 4.6 mm, particle size: 5 micron, pore size: 100Å); Gradient: 30-50% 20min 1.2 mL min^{-1}

Compound **11b**

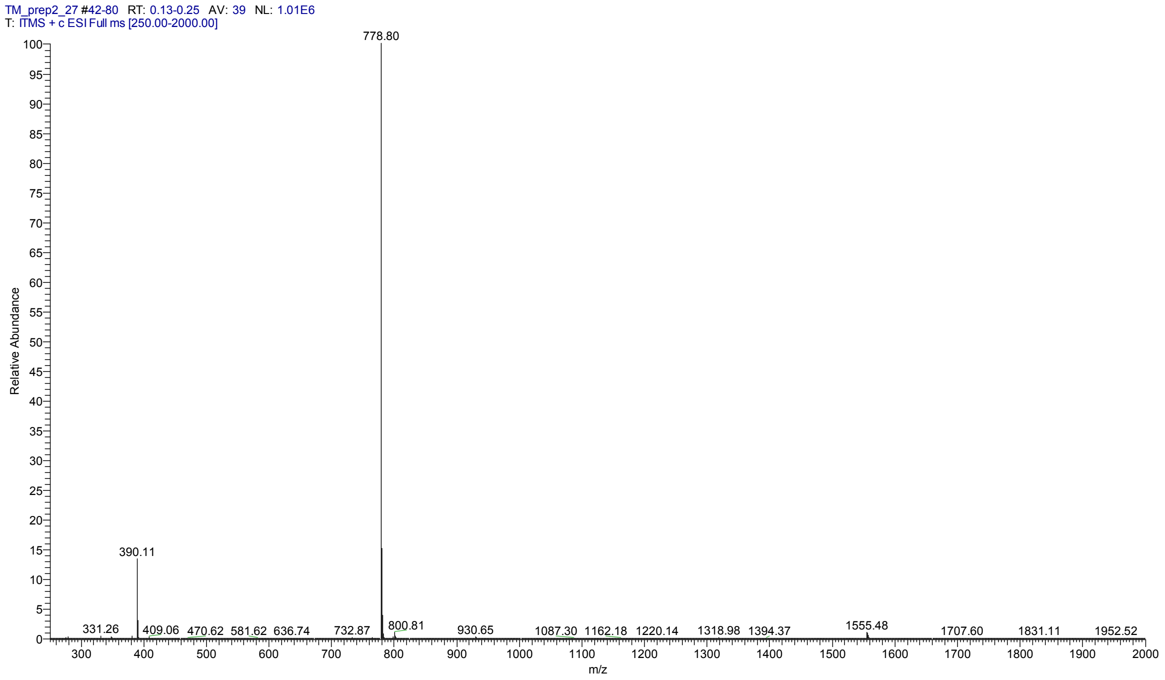

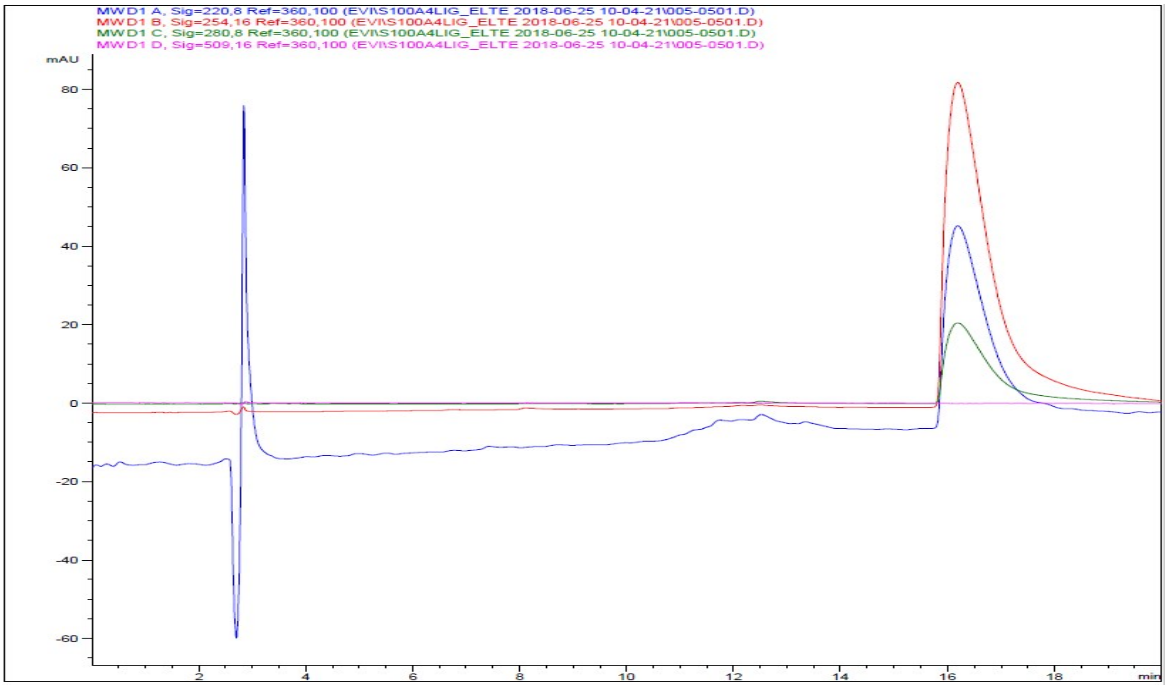

Column: Phenomenex Luna C18 (250 x 4.6 mm, particle size: 5 micron, pore size: 100Å); Gradient: 30-50% 20min 1.2 mL min^{-1}

Compound **12b**

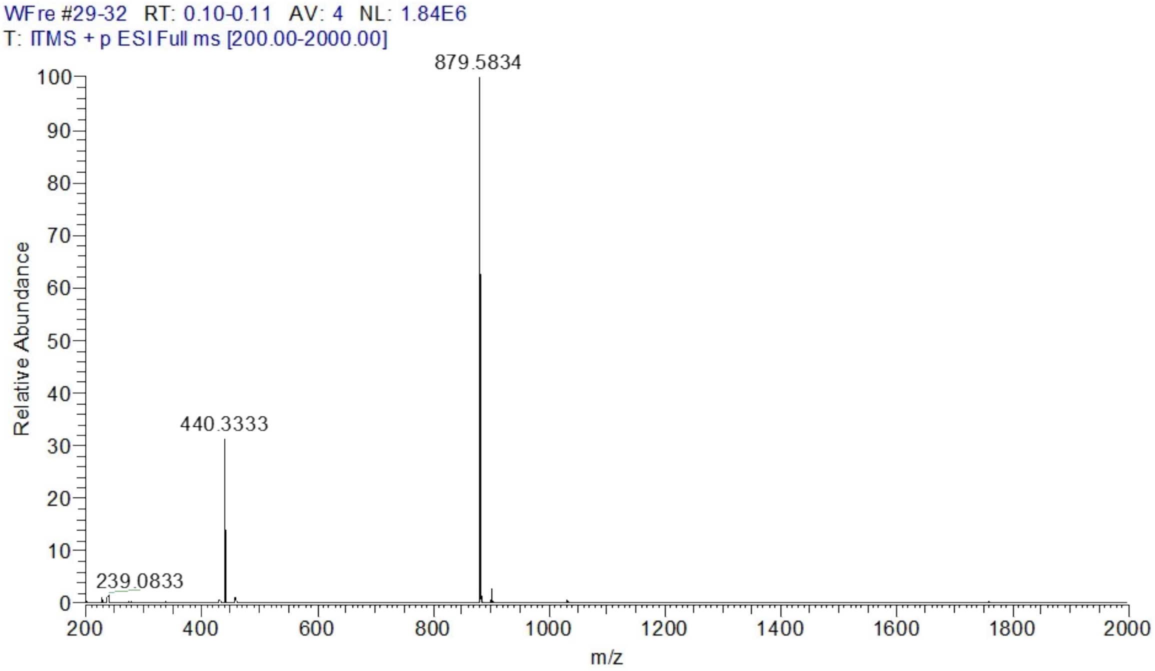

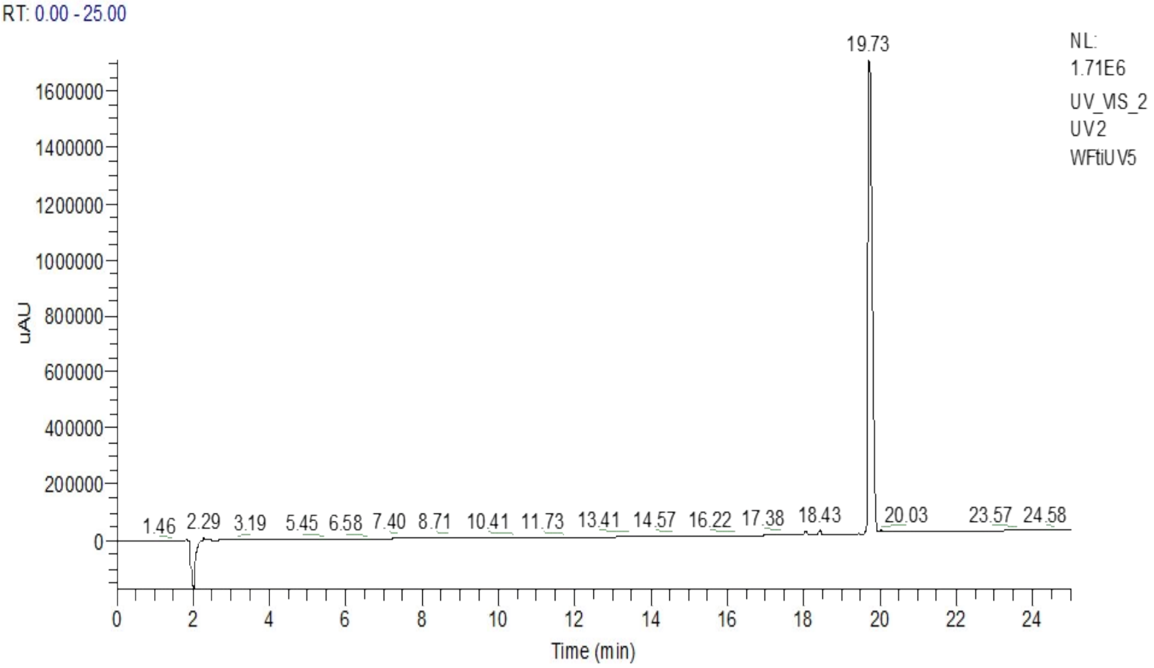

Column: Phenomenex Luna C18 (250 x 4.6 mm, particle size: 5 micron, pore size: 100Å); Gradient: 5-80% 25min 1.2 mL min^{-1}

SI References

1. B. Kiss, P. Ecsedi, M. Simon, L. Nyitray Isolation and Characterization of S100 Protein-Protein Complexes. *Methods in Mol. Biol. (Clifton, N.J.)* **1929,** 325-338 (2019).
2. É. Bartus, Z. Hegedüs, E. Wéber, B. Csipak, G. Szakonyi, T. A. Martinek De Novo Modular Development of a Foldameric Protein–Protein Interaction Inhibitor for Separate Hot Spots: A Dynamic Covalent Assembly Approach. *ChemistryOpen* **6,** 236-241 (2017).
3. C. Stark, B. J. Breitkreutz, T. Reguly, L. Boucher, A. Breitkreutz, M. Tyers BioGRID: a general repository for interaction datasets. *Nucl. Acids Res.* **34,** D535-539 (2006).
4. N. Orii,M. K. Ganapathiraju Wiki-pi: a web-server of annotated human protein-protein interactions to aid in discovery of protein function. *PloS One* **7,** e49029-e49029 (2012).
5. M. E. Fahey, M. J. Bennett, C. Mahon, S. Jäger, L. Pache, D. Kumar*, et al.* GPS-Prot: A web-based visualization platform for integrating host-pathogen interaction data. *BMC Bioinformatics* **12,** 298 (2011).
6. S. Orchard, M. Ammari, B. Aranda, L. Breuza, L. Briganti, F. Broackes-Carter*, et al.* The MIntAct project--IntAct as a common curation platform for 11 molecular interaction databases. *Nucl. Acids Res.* **42,** D358-363 (2014).
7. P. Shannon, A. Markiel, O. Ozier, N. S. Baliga, J. T. Wang, D. Ramage*, et al.* Cytoscape: a software environment for integrated models of biomolecular interaction networks. *Genome Res.* **13,** 2498-2504 (2003).
8. A. M. Watkins, R. Bonneau, P. S. Arora Side-Chain Conformational Preferences Govern Protein–Protein Interactions. *J. Am. Chem. Soc.* **138,** 10386-10389 (2016).
9. M. A. Simon, P. Ecsédi, G. M. Kovács, Á. L. Póti, A. Reményi, J. Kardos*, et al.* High-throughput competitive fluorescence polarization assay reveals functional redundancy in the S100 protein family. *The FEBS J. doi.org/10.1111/febs.15175*
